# Extracting, filtering and simulating cellular barcodes using CellBarcode tools

**DOI:** 10.1038/s43588-024-00595-7

**Published:** 2024-02-19

**Authors:** Wenjie Sun, Meghan Perkins, Mathilde Huyghe, Marisa M. Faraldo, Silvia Fre, Leïla Perié, Anne-Marie Lyne

**Affiliations:** 1 Institut Curie, Université PSL, Sorbonne Université, CNRS UMR168, Laboratoire Physico Chimie Curie, Paris, France; 2 Institut Curie, Laboratory of Genetics and Developmental Biology, PSL Research University, INSERM U934, CNRS UMR3215, Paris, France; 3 INSERM U900, Paris, France; 4 MINES ParisTech, CBIO-Centre for Computational Biology, PSL Research University, Paris, France

**Keywords:** Software, Bioinformatics, Next-generation sequencing, Development

## Abstract

Identifying true DNA cellular barcodes among polymerase chain reaction and sequencing errors is challenging. Current tools are restricted in the diversity of barcode types supported or the analysis strategies implemented. As such, there is a need for more versatile and efficient tools for barcode extraction, as well as for tools to investigate which factors impact barcode detection and which filtering strategies to best apply. Here we introduce the package CellBarcode and its barcode simulation kit, CellBarcodeSim, that allows efficient and versatile barcode extraction and filtering for a range of barcode types from bulk or single-cell sequencing data using a variety of filtering strategies. Using the barcode simulation kit and biological data, we explore the technical and biological factors influencing barcode identification and provide a decision tree on how to optimize barcode identification for different barcode settings. We believe that CellBarcode and CellBarcodeSim have the capability to enhance the reproducibility and interpretation of barcode results across studies.

## Abstract (web summary)

The authors introduce two cellular barcoding tools: CellBarcode, for extracting and filtering diverse DNA barcodes from bulk and single-cell sequencing data; and CellBarcodeSim, for simulating barcoding experiments, thus enabling the investigation of the impact of biological and technical factors on barcode detection.

## Main

DNA cellular barcoding is a high-throughput approach widely used to follow lineage^1,2^ in different fields such as hematopoiesis, development^3–5^, cancer^6–9^ and infection dynamics^10^. It uses a unique and heritable DNA sequence incorporated into the genome of an ancestor cell, which is then detected via sequencing in its progenies.

In the earliest approaches, progenitor cells were prospectively transduced ex vivo with libraries of fixed-length oligonucleotides^11^. More recently, to avoid extraction and reimplantation of progenitor cells, in vivo recombining genetic cassettes have been incorporated in transgenic organisms. Many innovative approaches have produced these in situ genetic labels^12–16^, with the majority detected via short-read sequencing. Barcodes are now detected with single-cell RNA sequencing (scRNA-seq)^14–17^, coupling lineage with fine-grained phenotyping.

DNA barcodes detected via next-generation sequencing (NGS) are subject to various sources of error, resulting in the identification of spurious barcodes. All barcode types are affected by PCR error/bias^18^ and sequencing error; in situ barcodes suffer additionally from the inability to control the distance between barcodes^19,20^. Biological factors such as the number of barcodes and clone size can impact barcode detection but have rarely been investigated^21^. To extract and identify true from spurious barcodes, many different bioinformatic filtering strategies have been proposed. However, little comparison of the various strategies has been published and most publications use their own ‘in house’ processing pipelines. This is problematic in terms of interpretation of results across studies and reproducibility. Both guidelines on how filtering strategies and their parameterization impact barcode quantification and broadly applicable tools are required^22^.

Beside tools for visualization and data exploration^23–25^ three tools have been developed to extract DNA barcodes from NGS data: genBaRcode^26^, Bartender^27^ and CellTagR^28^. While each has demonstrated utility, they are either restricted in the diversity of barcode types supported (CellTagR, genBaRcode) or the analysis strategies implemented (all of the above). No tools provide a framework to simulate barcode experiments and investigate the technical and biological factors impacting barcode detection. There is a need for more versatile tools to extract, identify and simulate barcodes.

To address these issues, we developed two tools: CellBarcode, an R Bioconductor package for barcode extraction and filtering, and CellBarcodeSim, a barcode simulation kit that faithfully reproduces barcoding experiments. We demonstrate, using simulated and experimental datasets, that CellBarcode allows users to implement various filtering strategies for bulk or single-cell datasets. Using CellBarcodeSim to simulate barcoding experiments, we investigated potential technical and biological factors impacting the reliability of barcode identification, confirmed with experimental datasets. We recapitulated our results into a decision tree to guide researchers on which filtering strategy is most appropriate for their setting. Overall, we present efficient and versatile tools to extract and identify barcodes from errors, and provide advice on how best to analyze barcoding experiments in a range of biological situations.

## Results

### CellBarcode package

**Fig. 1:**
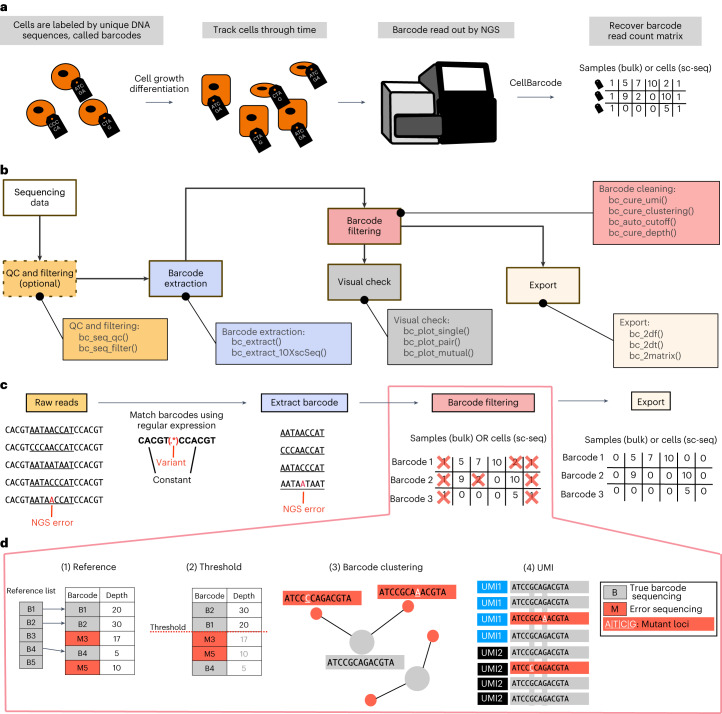
CellBarcode package to extract and identify lineage barcodes. **a**, Barcode experiment scheme. Cells are labeled with genetic barcodes, divide and differentiate, with progeny inheriting the barcode. Barcodes are read out by NGS in descendant cells. CellBarcode allows extraction, filtering and identification of barcodes from NGS data and returns a barcode count matrix for further analysis. sc-seq, single-cell sequencing. **b**, Diagram of barcode sequencing data processing with CellBarcode. CellBarcode reads the raw sequencing data (FASTQ, FASTA, BAM/SAM files or R object) and checks the quality control (QC and filtering functions) before extracting the barcode sequences (barcode extraction functions). Barcodes are then filtered to remove PCR and sequencing errors using different filtering strategies (barcode cleaning functions). After filtering, barcode data can be plotted with the visual check functions and exported as a barcode frequency matrix (export functions). **c**, Example of barcode processing workflow using CellBarcode. Barcodes (underlined) are extracted from raw sequences using a regular expression (sequence in bold) that depends on the barcode type. Barcodes are then filtered, as detailed in **d**, to eliminate spurious barcodes and exported. **d**, The four most commonly used barcode filtering strategies. Gray indicates true barcodes and red indicates spurious barcodes. (1) Reference library filtering: barcodes B1, B2 and B3 that match the reference list are considered true barcodes, M3 and M5 are removed. (2) Threshold filtering: barcodes that have a read number superior or equal to the threshold of 20 are kept (B1 and B2) and barcodes below the threshold are removed (M3, M5 and B3). (3) Cluster filtering: barcodes with an edit distance smaller than a threshold to a more abundant barcode are eliminated. Here, two barcodes have one substitution difference (mutant loci in white) from an abundant barcode and will be deleted. (4) UMI filtering: usually involves retaining the most abundant sequence per UMI followed by a UMI count threshold per barcode.

We developed the CellBarcode R package, which provides a toolkit for barcode pre-processing, including steps from generating the FASTQ quality control information to exporting the data into a read count matrix (Fig. 1a). Using the read quality control and filtering functions of CellBarcode, users can check sequencing quality, remove low-quality sequences and get an overview of read diversity. Barcodes can then be extracted from the FASTQ or BAM file by defining a regular expression matching the structure of the lineage barcode and its surrounding flanking sequence (see Supplementary Vignette 1 for examples and a detailed description of this process); both fixed-length and variable-length barcodes can be extracted, and mismatches in the flanking regions are allowed (bulk analysis only). Once the raw barcodes have been extracted, filtering functions can remove spurious barcode sequences using commonly applied strategies. In addition, the package provides functions for visualizing the barcode read count distribution per sample and across replicates (Fig. 1b,c).

The four main filtering strategies generally applied to barcoded data are implemented in CellBarcode (Fig. 1d). (1) Reference filtering: barcodes not matching with the reference list are eliminated. The reference list is either generated by sequencing the viral barcode libraries^5^ or enumerating all possible barcodes using knowledge of barcode structure^19^. (2) Threshold filtering: barcodes are retained if their read number (depth) surpasses a specified threshold^5^. CellBarcode has a manual or an automatic threshold option (see ‘Barcode filtering’ in Methods). (3) Cluster filtering: barcodes that have an edit distance smaller than a specified threshold to a more abundant barcode are eliminated^29^. (4) Unique molecular identifier (UMI) filtering: if UMIs are added to DNA molecules during library preparation, several optional filtering steps can be applied, including extracting the most abundant barcode per UMI and threshold filters on the read count per UMI or UMI count per barcode. These four filtering strategies can be used individually or in combination, and we later advise on when to apply each strategy using simulated data with CellBarcodeSim. See Supplementary Vignettes 1 and 2 for examples of all major use cases.

In summary, CellBarcode is a versatile and open-source tool that works on all major operating systems and is capable of analyzing a wide variety of DNA barcode types with commonly applied filtering strategies. The key assets of CellBarcode are its speed, the ability to deal with UMI data and the extraction of barcodes from scRNA-seq data (Supplementary Table 1). Efficient C++ code accelerates heavy tasks compared with other packages; barcode extraction and cluster filtering are 20 and 70 times faster than using genBaRcode (Supplementary Fig. 1).

### Comparing barcode filtering strategies using CellBarcodeSim

**Fig. 2:**
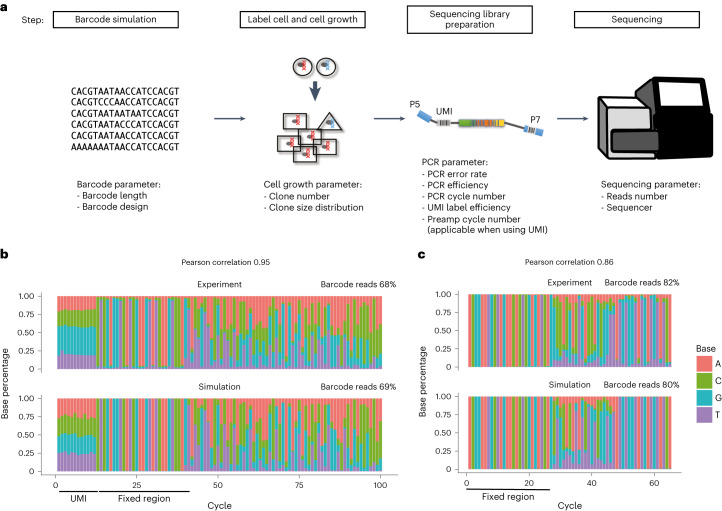
Cellular barcode sequencing simulation. **a**, Schematic of barcoding experiment simulation with CellBarcodeSim and the parameters that can be tuned at each step, starting with simulation of a barcode library, cell labeling and clonal expansion, PCR amplification, and finally sequencing. The round shape represents undifferentiated cells, the triangle and rectangles represent differentiated cell types. **b**,**c**, Stacked bar plots, created using CellBarcode, showing the percentage of bases for the VDJ barcode dataset with UMI (**b**) and a random barcode dataset (**c**) across each sequencing cycle. Each column represents a sequencing cycle, with color and height indicating the base and proportion, respectively. Both simulated and real experimental data are presented for each dataset. The percentage of total reads matching the regular expression is indicated, as well as the Pearson correlation between the most abundant base per sequencing cycle. Fixed and/or UMI regions are annotated below the heatmap. The VDJ barcode dataset is the MEF line experiment data with 12,500 cells from ref. ^20^; the random barcode dataset is from ref. ^30^. Simulation details for each dataset are provided in Methods. Source data

The CellBarcode package provides a variety of functions for barcode filtering, but choosing a filtering strategy and its parameterization in a given experimental setting is challenging. With this in mind, we developed a barcode simulation toolkit, called CellBarcodeSim, which produces in silico barcoding data mimicking bulk DNA-seq experimental situations by varying a number of technical and biological factors. CellBarcodeSim covers production of a barcode library, cell barcode labeling and clonal expansion, construction of full sequencing reads including flanking sequences and UMIs when desired, and finally PCR amplification and sequencing with the inclusion of error (Fig. 2a and ‘DNA cellular barcode sequencing simulations’ in Methods). In total, CellBarcodeSim provides 10 configurable parameters for non-UMI and 13 for UMI sequencing libraries (Fig. 2a). Tens of thousands of clones can be simulated on a standard laptop (16 Gb random-access memory), covering most experimental situations. Two types of barcode library can be simulated with CellBarcodeSim (see ‘DNA cellular barcode sequencing simulations’ in Methods) while other types of barcode can be uploaded as a list. Comparing the known barcodes from simulation with the output of CellBarcode can guide users in their choice of filtering strategy and its parameterization. Overall, CellBarcodeSim simulates barcoding experiments varying multiple technical and biological factors.

Before exploring how different parameters impact barcode identification across filtering strategies, we first checked that CellBarcodeSim could reproduce the expected output of a barcoding experiment. We simulated two experimental datasets: lentiviral fixed-length 20-bp barcodes recovered from myeloid cells^30^; and a variable, diversity, joining (VDJ)-barcoded dataset with UMIs recovered from mouse embryonic fibroblast (MEF) cells^20^ (see ‘Acquisition, analysis and simulation of experimental data’ in Methods). We showed that CellBarcodeSim outputs the same read structure and similar proportion of reads matching the regular expression as the experimental data (Fig. 2b,c), with high Pearson correlation between the proportion of the most abundant base at each sequencing cycle between the simulated and experimental data (Fig. 2b,c).

Next, to investigate the key factors impacting barcode identification for different filtering strategies, we first designed a default scenario for non-UMI barcode libraries (see ‘DNA cellular barcode sequencing simulations’ in Methods and Supplementary Table 2) and then 25 alternative scenarios varying key biological and experimental parameters (Supplementary Table 2). After randomly simulating each scenario 30 times, we applied 4 different filtering strategies (read count thresholding, reference library, clustering and UMI filtering). To evaluate the filtering performance, for each simulation we computed barcode recall (the proportion of true barcodes found in the output) and precision (the proportion of output barcodes that are true) using the known ground truth. We then computed the area under the precision–recall curve (PR AUC) across a range of thresholds (Supplementary Fig. 2) to indicate how well filtering methods separate true from spurious barcodes regardless of threshold.

**Fig. 3:**
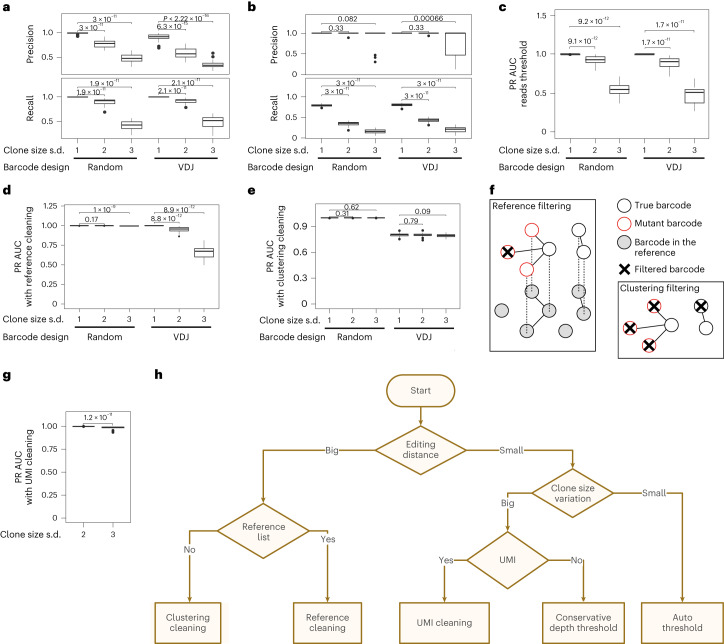
Benchmarking barcode filtering strategies with simulated data. **a**,**b**, Percentage precision and recall of true barcodes for different threshold filtering using read proportion thresholds of 0.0001 (**a**) and 0.001 (**b**). Several scenarios with two types of barcode (random and VDJ) and three different clone size variations across barcodes are compared. **c**, PR AUC using threshold filtering for two types of barcode (random and VDJ) and three different clone size variations across barcodes. **d**, The same as in **c** after reference filtering. **e**, The same as in **c** after cluster filtering. **f**, Diagrams depicting reference library filtering and cluster filtering advantages and drawbacks. Reference library filtering removes spurious barcodes that are not in the library but keeps spurious barcodes that match a barcode in the reference library. Cluster filtering removes low abundance barcodes that are similar to abundant barcodes. This can result in the removal of true barcodes that have sequence similarity to another true barcode, for example, if the barcode library has small edit distance. **g**, PR AUC after UMI filtering for variable-length VDJ barcodes for two higher clone size variations (log clone size s.d. of 2 and 3). An initial filtering based on UMI count greater than ten reads was performed before computing PR AUC. **h**, Barcode filtering decision tree. Except where otherwise specified, each simulated scenario has the reference parameters from Supplementary Table 2: 30 simulations, 300 induced barcodes with log clone size mean 1.2, PCR cycle 30, PCR efficiency 0.705, PCR error 1 × 10^−6^, reads per cell 50 and sequencing profile HiSeq 2000. Specifically for **h**, the number of PCR cycles before and after UMI tagging are 10 and 20, respectively, with 8-bp UMI and tagging efficiency 0.02. The median and IQR (the difference between the 75th and 25th percentiles of the data) are shown in the boxplot over 30 simulations, and the outliers (beyond the whiskers of Q3 + 1.5 × IQR or Q2 − 1.5 × IQR) plotted as dots. The two-sided Wilcoxon test is applied to compare the precision, recall or AUC of different simulation conditions. Source data

We first consider read count threshold filtering. In all scenarios, there is an overlap between the read count distributions of error and true barcodes combined across simulations (Supplementary Fig. 3); therefore, it is impossible to choose a read threshold to perfectly separate true from spurious barcodes. Using a read threshold involves a trade-off between the recall and precision of barcode detection, with a higher threshold removing more spurious barcodes but also more true barcodes (Fig. 3a,b). Surprisingly, the factor that had the largest impact on PR AUC was one of the biological factors: the standard deviation (s.d.) of the log clone size (where log denotes the natural logarithm), with smaller clone size variation showing larger PR AUC (Fig. 3c and Supplementary Figs. 4 and 5a). When log clone size s.d. was 1, the PR AUC reached 1 regardless of other factors, including barcode type or mean clone size (Fig. 3c and Supplementary Figs. 4 and 5a). Comparing precision and recall for different thresholds, we observed the expected trend of increased recall but decreased precision as the threshold became less stringent (Fig. 3a,b and Supplementary Figs. 5b and 6). When there is high variability in the number of cells labeled by each barcode (log clone size s.d. ≥ 2), recall needs to be compromised to avoid calling spurious barcodes. This leads to a significant loss of true barcodes, predominantly affecting barcodes of small clones that have similar read count to error barcodes derived from much larger clones (Fig. 3a,b and Supplementary Figs. 5b and 6). This loss of true barcodes can preclude robust statistical analysis downstream (Supplementary Fig. 5c).

To validate the finding about the impact of clone size s.d., we used an unpublished dataset in which Cas9-expressing mice intestinal organoids were infected with libraries of guide RNAs (gRNAs) designed to knock out specific genes (see ‘CRISPR gRNA dataset’ in Methods). While not a standard barcode, each specific knockout acts as a clonal label and can be extracted by CellBarcode using a regular expression targeting the constant primer region. Two time points were analyzed, 24 hours and 7 days, with clone size variation increasing over time due to fitness effects of the gRNAs. Using CellBarcodeSim to simulate the experiment, we successfully reproduced the percentage of barcode-containing reads, and observed a change in the read count distribution, from bimodal with true and spurious barcode counts mostly separated at low clone size s.d., to unimodal with more overlap in true and spurious barcode counts at higher clone size s.d. (Supplementary Fig. 7, top row). These same trends were observed in the experimental data (Supplementary Fig. 7, bottom row). To verify the finding that the number of PCR cycles has limited impact on barcode recall (Supplementary Fig. 8), we used published data of mixes of seven MEF cell lines that each contain a unique known VDJ barcode^20^. Across the mixes, the total number of initiating cells was reduced and the number of PCR cycles correspondingly increased to produce a constant PCR product concentration, with the clone size ratios kept constant. Irrespective of the number of PCR cycles, CellBarcode identified the 7 known barcodes in each mix (with 1 spurious barcode at +4 PCR cycles) (Supplementary Fig. 8). Using CellBarcodeSim with matched parameters and varying the number of PCR cycles, we reproduced the separation of true and spurious barcode counts and the lack of change in the sequence frequency distribution (Supplementary Fig. 8). Using two experimental datasets, we therefore demonstrated that CellBarcodeSim can simulate real scenarios. Our simulation results of the large impact of the clone size s.d. and the limited impact of PCR cycle number on barcode identification were supported by these experimental data. Regarding filtering, we showed that the read count thresholding strategy is suboptimal at best, except for systems in which the clones have a similar number of cells. Some biological systems have been shown to differ in their proliferation capacities^31^, but for most of them this information is unknown. CellBarcodeSim is therefore a useful tool to simulate different scenarios, guiding researchers on the impact of thresholds on barcode identification and aiding in the interpretation of results.

An alternative strategy for barcode filtering is to match the extracted barcodes to a reference library when available. Using this approach for fixed-length barcodes, the distributions of true barcode read counts overlap less with those of spurious barcodes (Supplementary Fig. 9), and true barcode PR AUC was substantially improved, with most scenarios having a PR AUC of 1 (Supplementary Fig. 10), as suggested before^5^. We applied read count thresholding here after reference filtering to compute the PR AUC, enabling scenario comparison, although its use is optional. We note that read count threshold filtering is used to call true barcodes in the generation of the reference library itself, and even though these plasmid libraries have more homogenous barcode abundances than most biological experiments, the reference library suffers from the threshold-related problems described above and by others^21^. For variable-length barcodes such as VDJ barcodes, a reference library can be generated by simulating all possible combinations. Using this list had limited improvement in PR AUC (Fig. 3d and Supplementary Fig. 10) due to the small edit distance between some barcodes (many with edit distance <3; Supplementary Fig. 11a). Spurious sequences created by PCR or sequencing error can have the same sequence as a barcode in the reference library (Fig. 3f) and are not filtered out, impacting the precision (Supplementary Fig. 12). Overall, these results show that a reference library is a useful approach for fixed-length barcodes designed to have edit distances larger than 3, but is not useful for variable-length barcodes such as VDJ barcodes where the edit distance cannot be controlled.

Several studies have advocated cluster filtering to identify true barcodes^21,26,27^. With clustering, true barcodes are identified by comparing barcode sequences, usually with the assumptions that barcodes separated by very short edit distances are the result of PCR/sequencing errors and that the most abundant barcode in the cluster is the true barcode^21,26,27^. We used CellBarcodeSim to evaluate how cluster filtering performs compared with other filtering strategies. Cluster filtering improved the PR AUC of random barcodes compared with threshold filtering alone (Fig. 3e) and performed as well as reference library filtering (Fig. 3d,e), implying that it is the method of choice for the generation of a reference library, as previously suggested^18,21^. For variable-length barcodes such as VDJ barcodes, clustering performed worse or similar to threshold or reference library filtering (Fig. 3e and Supplementary Fig. 13) due to low recall (Supplementary Fig. 14), although the true barcode read counts overlap less with those of the spurious ones (Supplementary Fig. 14). This is linked to the short edit distance of some in situ barcodes, which are not PCR/sequencing errors as assumed by cluster filtering (Fig. 3f and Supplementary Fig. 11a). We previously developed a sequencing library preparation protocol for VDJ barcodes with UMIs^20^. We hypothesized that the addition of UMIs will improve the identification of true barcodes using cluster filtering. To test this hypothesis, we simulated VDJ barcode sequencing with UMIs for high clone size variation samples, which we identified as the most difficult scenario in which to apply this filtering (Supplementary Table 3). We observed that incorporating UMI information significantly improved the PR AUC for samples with large clone size variation (Fig. 3g), supporting the hypothesis that the addition of UMIs helps true barcode identification by cluster filtering for barcodes with low edit distance, such as VDJ barcodes. Overall, these results show that cluster filtering is an efficient method to identify barcodes in systems with large edit distance such as viral barcodes^18,32^. It is the method of choice if one had no reference library or to make a reference library for such barcodes^18,21^.

We summarized the findings of our comprehensive comparison in a decision tree to guide researchers on which strategy to apply to their data (Fig. 3h). In summary, our advice is: use reference library or cluster filtering if the barcoding system has a large edit distance (approximately ≥3); otherwise, if the barcode clone size variation is small, a read threshold would work. If the barcode clone size variation is large and the barcode system has a small edit distance, either UMIs need to be used or a stringent read count threshold implemented sacrificing true barcodes with low read count.

### Reference and cluster filtering of lentiviral barcodes

**Fig. 4:**
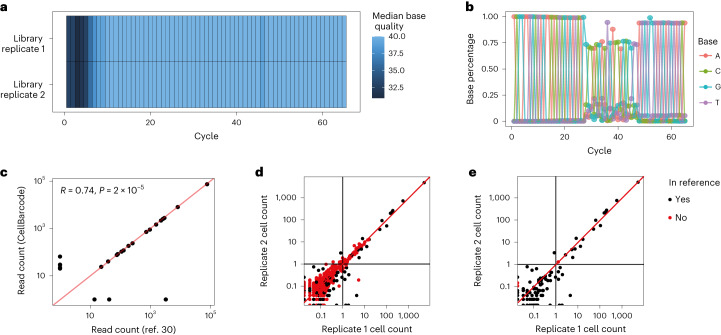
Lentiviral barcode sequencing analysis. **a**, Base quality heatmap made with CellBarcode. Each row is a sample, each column corresponds to a sequencing cycle and the color represents the median base Phred quality score. **b**, Base percentage plotted against the sequencing cycle number made with CellBarcode. The sequence shows a 20-bp barcode with fixed flanking regions either side. The color represents a base pair. **c**, Barcode normalized read count + 1 (by total 10^5^ reads) as filtered in the original paper^30^ versus using CellBarcode. Each dot is a barcode. The Spearman correlation and *P* value (two-sided) are shown in the top left corner. **d**, Barcode cell counts between the two technical replicates for the data without filtering. The read counts were normalized to cell counts. Each dot is a barcode with black indicating presence in the reference library provided in ref. ^30^. **e**, The same as in **d** but after cluster filtering. The filtering process involves removing barcodes that have a Hamming distance of less than 2 from a more abundant barcode. In **c**–**e**, the red line represents *y* = *x* and the black line indicates a threshold of one cell. Source data

To compare cluster and reference library filtering on biological data, we used CellBarcode to analyze paired technical replicates of 13,564 myeloid cells labeled with a random fixed-length barcode library^30^. Consistent with simulated random barcodes (Supplementary Fig. 11b), it showed a high edit distance (Supplementary Fig. 11c). First, we used CellBarcode to check the quality of the FASTQ file, plotting the base percentage and quality in each sequencing cycle (Fig. 4a,b). We successfully extracted and quantified the barcodes using CellBarcode as shown by the correlation with those in the original paper (Fig. 4c). Our results are also consistent with genBaRcode (Supplementary Fig. 15a) and Bartender analysis (Supplementary Fig. 15b), although we observe considerably more noise in the Bartender data, because it has fewer filtering steps implemented.

According to our decision tree, the methods to use for high-edit-distance barcodes are reference library or cluster filtering. We therefore extracted barcodes using either no filtering, reference library or cluster filtering and compared barcode cell count detected in technical repeats after normalizing read counts by total cell number (Fig. 4d). In biological data, as the identity of the true barcodes is unknown, we used the reference library provided in ref. ^30^. Without filtering, many barcodes not present in the reference library overlapped in read count distribution with those in the reference library, agreeing with our simulation results that read threshold filtering decreases the recall to ensure precision (Fig. 4d). Cluster filtering removed most of the barcodes absent from the reference library, leaving only one spurious sequence present in one cell, while keeping all the true barcodes with more than one cell (Fig. 4e). This confirms our simulation finding that cluster filtering can have the same efficacy as reference library filtering using barcodes with high edit distances.

### Read threshold filtering of in situ barcodes

**Fig. 5:**
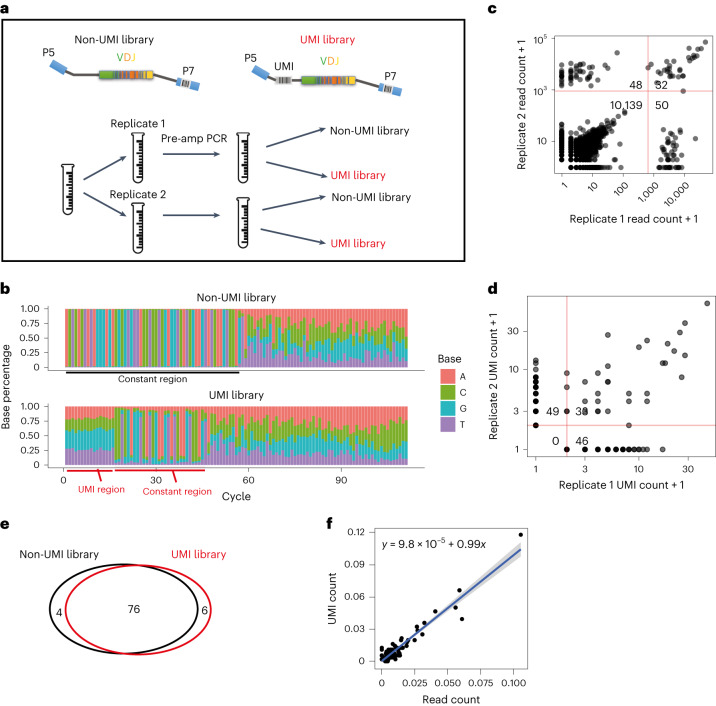
In vitro VDJ barcode analysis. **a**, Sequencing library design and sequencing scheme. A sample was divided into two technical replicates. After a first PCR amplification, each technical replicate was further divided into two for sequencing library preparation with and without UMIs. **b**, Stacked bar plot made with CellBarcode showing the base percentage for each sequencing cycle. Each column corresponds to a sequencing cycle; the color and height indicate the base and proportion, respectively. Both rows depict the same biological sample, with or without UMI for sequencing. The position of the regular expression (constant region) and the UMI are annotated. **c**, Barcode read counts between technical replicates for the non-UMI library without filtering. Automatic thresholds (marked by red lines) were applied to remove the errors in each technical replicate separately. The numbers show the barcode count in each of the four categories as divided by the threshold lines. Each dot represents a barcode. Plot made with CellBarcode; the dots are semi-transparent to show overlap. **d**, Barcode UMI count between technical replicates with UMI library. The data were first filtered, retaining UMI with at least ten reads. The red lines indicate a UMI count threshold of 1. The number of barcodes in each of the four categories as divided by the threshold lines is annotated. Each dot represents a barcode. Plot made with CellBarcode; the dots are semi-transparent to show overlap. **e**, Comparing the number of barcodes identified in the non-UMI library and the UMI library in one technical replicate. For the non-UMI library, the automatic threshold was applied as shown in **c**. For the UMI library, the same filtering steps were applied as in **d** with the addition of a UMI count threshold of 1. **f**, Barcode read count after filtering between the non-UMI library and the UMI library for one of the technical replicates. The read counts were renormalized to 1. A linear regression was fitted, and the fitted line (and shaded area of 95% confidence interval) and its parameters are written on the plot. Each dot represents a barcode. Source data

Variable-length barcodes such as VDJ barcodes are the most challenging to identify in noisy data due to the short-edit-distance barcodes generated. To explore whether our CellBarcode simulation results would hold in experimental variable-length barcode data, we made use of our unpublished in vivo VDJ barcode data from mouse mammary glands, for which we have both UMI and non-UMI data from the same sample (Fig. 5a,b). Using the known read structures of the two sequencing libraries (Fig. 5b), we extracted the barcodes and applied automatic read threshold filtering and UMI filtering to the non-UMI and UMI samples, respectively (Fig. 5c,d), illustrating the versatility of CellBarcode to extract barcodes from a variety of structures (see ‘VDJ barcode mammary gland dataset’ in Methods). For different UMI read count thresholds, we observed that the number of barcodes reached a plateau (Supplementary Fig. 16a). At this plateau, in one duplicate sample, we identified 80 barcodes in the non-UMI library, and 82 barcodes in the UMI library with 76 barcodes overlapping (87%; Fig. 5e).

In these data, the biggest clones had about 100 times higher read/UMI count compared with the smallest clones, corresponding to a log clone size s.d. of 1, the lowest considered in our simulations (Fig. 5c,d). The clone sizes in the UMI and non-UMI libraries after threshold filtering (normalized reads or UMI count) correlated very well (Fig. 5f), with most of the inconsistent barcodes being small clones. This result supports our simulation conclusion that automatic read thresholding performs well in experimental settings with small clone size variation. We observed more spurious barcodes in both UMI and non-UMI results from Bartender (Supplementary Fig. 16b,c), indicating the importance of read or UMI read count thresholds that are not implemented in Bartender.

### Using CellBarcode to analyze scRNA-seq data

**Fig. 6:**
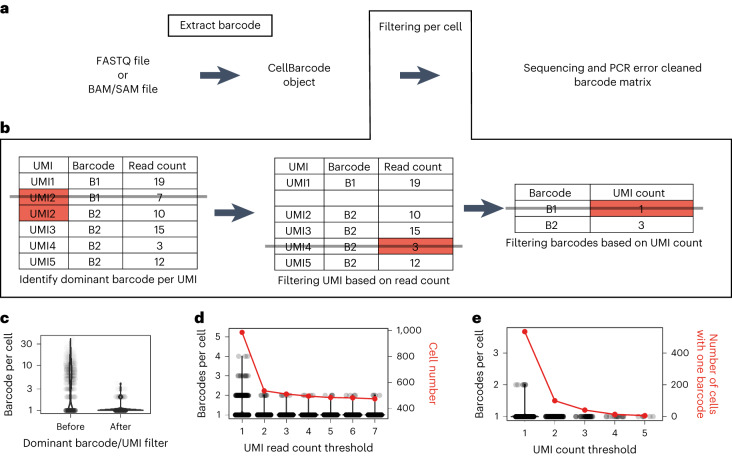
scRNA-seq cellular DNA barcode analysis. **a**, Diagram of how lineage-barcoded single-cell sequencing data are processed with CellBarcode. Input files can be FASTQ or BAM/SAM files. The lineage barcodes are extracted, filtered and exported for subsequent analysis. **b**, Filtering steps for single-cell sequencing lineage barcode data implemented in CellBarcode. First, for each UMI the dominant barcode is identified and other barcodes are removed; then UMIs with a read count below a threshold are removed. For each barcode, the number of UMIs is counted and the barcodes are filtered based on a UMI count threshold. **c**, The number of lineage barcodes found per cell before and after filtering barcodes based on the dominant barcode per UMI using the VDJ scRNA-seq data from ref. ^35^. The *y* axis is the barcode number in a cell, each dot represents a cell and the distribution is shown by the violin plot. **d**, The number of lineage barcodes per cell (corresponding to the left *y* axis, black) and the cell number (corresponding to the right *y* axis, red) for different thresholds of read per UMI. The data were first processed with the dominant barcode per UMI filter. Each black dot represents a cell and the violin plot shows the distribution of the barcode number per cell. **e**, The number of lineage barcodes per cell (corresponding to the left *y* axis, black) and the number of cells with a unique barcode (corresponding to the right *y* axis, red) for different thresholds of UMI count per barcode. The data were first processed with the dominant barcode per UMI filter and the UMI read threshold ≥2. Each dot represents a cell and the distribution is shown by the violin plot. The red line plot represents the number of retained, unique barcoded cells after applying different UMI count filters described in the *x* axis. Source data

Finally, we designed CellBarcode to extract and identify lineage barcodes from single-cell omics data. To this end, CellBarcode is equipped with functions to process barcodes from the most popular technologies such as 10x Genomics or Smart-seq (Fig. 6a). In this section, we use the term ‘cell barcode’ to refer to the unique barcode labeling each cell from the single-cell sequencing protocol, and ‘lineage barcode’ to refer to the barcode added during a lineage tracing experiment. Input to CellBarcode is flexible, allowing FASTQ and BAM/SAM files, either one file for all cells (as for 10x Genomics scRNA-seq) or one file per cell (as for Smart-seq2), and BAM/SAM files pre-tagged with cell barcodes and UMIs, such as those output by the 10x Genomics software CellRanger. We illustrate the use of CellBarcode on scRNA-seq data but it applies to many types of lineage-barcoded single-cell omics data, such as single cell ATAC-sequencing^33,34^. The potential (but optional) filters include (1) extract dominant barcode per UMI, (2) filter UMIs using a read count threshold and (3) filter lineage barcodes using a UMI count threshold (Fig. 6b). The user must choose various thresholds, and here we distinguish two experimental scenarios from published data: (1) a unique lineage barcode per cell, such as low-concentration lentivirus infection^17^ or heterozygous inducible VDJ barcode^35^, and (2) multi-barcodes per cell, for example, high-concentration lentiviral infection such as the CellTag barcode system^36^.

To compare the performance of CellBarcode to that of CellTagR, a dedicated package for analysis of barcoded scRNA-seq data, we replicated the CellTagR demo analysis pipeline (https://github.com/morris-lab/CellTagR) with CellBarcode on the multi-barcode per cell data from ref. ^36^. Applying the same steps and parameters (see ‘CellTag barcode scRNA-seq dataset’ in Methods), CellBarcode obtained similar results to CellTagR (Supplementary Fig. 17a,b) with 20% less runtime (Supplementary Fig. 17c,d). CellTagR supports only the extraction of CellTag barcodes, whereas, to illustrate the versatility of CellBarcode, we extracted variable-length VDJ barcodes from scRNA-seq data from ref. ^35^ and obtained similar barcodes and quantification to the original paper (Supplementary Fig. 18).

To illustrate how CellBarcode can help users select the different filtering thresholds, we counted the number of lineage barcodes retrieved per cell for various types of filtering in VDJ barcoding data from ref. ^35^. Due to the introduction of one VDJ cassette in one allele of the mouse genome, each cell in this dataset has only one lineage barcode. We observed a trade-off between the accuracy of lineage barcode retrieval (that is, the proportion of cells with one unique lineage barcode) and the total number of lineage-barcoded cells retained for analysis. We first filtered to take the dominant lineage barcode per UMI, as the combination of high-diversity cell and UMI barcodes for each read can be assumed unique, which dramatically reduced the number of barcodes per cell compared with the raw data (Fig. 6c). Using different minimum read-count-per-UMI thresholds, we found that the number of barcodes per cell was easily restricted to a maximum of 2 with a threshold of 2 (Fig. 6d). Increasing the read-count-per-UMI threshold further resulted in the loss of many cells for analysis (Fig. 6d). Complementing the read-count-per-UMI filtering with a UMI-count-per-barcode filter of 2, we obtained 1 identifiable lineage barcode per cell (Fig. 6e). These thresholds will depend on each specific dataset, for example, with low sequencing depth, even without read-count-per-UMI or UMI-count-per-cell filtering, most cells have one unique lineage barcode as observed in the ref. ^17^ dataset (Supplementary Fig. 19).

To conclude, in addition to an improvement in runtime, CellBarcode can extract and identify lineage barcodes in scRNA-seq data from many different barcode designs due to its flexible use of regular expressions. Moreover, CellBarcode implements several filtering strategies to identify true from spurious lineage barcodes in single-cell data, and produces figures helping the user choose a strategy and its parameterization.

## Discussion

In this paper, we presented CellBarcode, a versatile R package for analysis of barcoding data, and CellBarcodeSim, a pipeline to simulate barcoding experiments. While we designed the simulation tool to test and parameterize filtering approaches for barcode identification, it can be employed in a similar vein for experimental design; for example, users can investigate the impact of different barcode lengths, UMI or non-UMI libraries and sequencing depths in their biological scenario. We highlight, however, that this is complicated by the combination of unknown biological factors and final filtering approach.

A previous study^21^ suggested not to use cluster filtering as it can result in the removal of true barcodes. However, both our simulations and tests on real data show that cluster filtering performs well when the barcode edit distance is large enough (≥3 in our simulations) compared with realistic low levels of PCR/sequencing error. We would therefore refine the statement from ref. ^21^ to add that cluster filtering can be successfully used when the edit distance is sufficiently high, even in the case of high clone size variation.

We modeled clone size using a log-normal distribution based on our analysis of T-cell receptor clones (Supplementary Fig. 20), and while users of CellBarcodeSim can also opt for a power-law distribution, we hope to add more detailed models in future versions of the tool (such as one based on ref. ^37^). Indeed, in most systems, the clone size distribution is unknown; in this case CellBarcodeSim can be used to investigate the impact of filtering strategies on barcode identification under different assumptions and can aid users in their biological interpretation. Further simulation work is also required to test the impact of filtering on barcode quantification.

CellBarcodeSim makes many other assumptions about the processes involved to simulate barcoding data. Barcode library production is modeled with simple distributions rather than separately modeling the stages of transfection, growth and sampling. The fixed-length Hamming^38^ barcodes simulated using the DNABarcodes package are filtered to remove many sources of error problematic for PCR, such as barcodes containing triplets or with GC bias. The PCR simulation assumes that the amount of starting material is large enough to ignore contamination and does not model factors such as non-specific hybridizations. Indeed, we do not expect our simulation to quantitatively model all possible effects of the complex PCR process. Researchers interested in specific sources of error, such as those introduced during barcode library preparation, or using a non-standard protocol where the PCR primer does not target the constant flanking region, would need to adapt the simulation.

CellBarcodeSim calls external tools such as the ART NGS read simulator^39^, the DNABarcodes R package to simulate fixed-length barcodes^40^ and IGoR (Inference and Generation of Repertoires) to simulate VDJ barcodes^41^, which could be a concern in terms of longevity. ART is a mature and heavily used tool with no updates required and containing pre-built error profiles for all the major sequencers. The packages simulating barcodes are less mature and barcode-type specific, but CellBarcodeSim can be easily updated allowing other tools to feed in.

## Methods

### Ethics statement

All studies and procedures involving animals were in accordance with the recommendations of the European Community (2010/63/UE) for the Protection of Vertebrate Animals used for Experimental and other Scientific Purposes. Approval was provided by the ethics committee of the French Ministry of Research (reference APAFIS 34364-202112151422480). We comply with internationally established principles of replacement, reduction and refinement in accordance with the Guide for the Care and Use of Laboratory Animals (NRC 2011). Husbandry, supply of animals, as well as maintenance and care in the Animal Facility of Institut Curie (facility license C75–05–18) before and during experiments fully satisfied the animal’s needs and welfare. Mouse breeding was in a specific pathogen-free animal facility and animals were co-housed with housing conditions using a 12 h light/12 h dark cycle, temperature between 20 °C and 24 °C, and average humidity between 40% and 70%.

### DNA cellular barcode sequencing simulations

We simulated the DNA cellular barcode sequencing data using CellBarcodeSim (version 1.0) with 5 steps: (1) lineage barcode simulation, (2) barcode labeling, (3) clonal expansion, (4) PCR amplification, and (5) sequencing.

#### Lineage barcode simulation

Two types of barcode library can be simulated with CellBarcodeSim (‘random barcodes’ with uniform probability and fixed length, and ‘Hamming barcodes’ with uniform probability, fixed-length and a minimum Hamming distance between sequences) while other types of barcode can be uploaded as a list. In addition, three libraries were simulated and uploaded in the package: 14-bp random barcodes, 14-bp Hamming barcodes with minimum distance 3 simulated using DNABarcodes^40^, and variable-length VDJ barcodes^20^ simulated using an external package IGoR.

For the simulation study, a list of possible barcodes was simulated for three types of barcode and barcodes were randomly sampled from this list to label cells. The fixed-length uniform-probability ‘random barcodes’ were generated with stri_rand_strings from stringi package. To generate ‘Hamming barcodes’ with a minimum Hamming distance of 3, we used the create.dnabarcodes function from the DNABarcodes package^40^. The barcode length can be defined by the user. In this simulation study, we tested 14 or 10 base pairs. Lastly, for the variable-length ‘VDJ barcodes’^20^, a list of 1 × 10^7^ VDJ barcodes to sample from was generated using IGoR^41^. To ensure the simulated VDJ barcodes resemble those produced in vivo, the parameters of the Bayesian network model used to generate the barcode space were inferred using IGoR from the VDJ barcode sequencing data in mammary gland tissue (Supplementary Data 1 and 2). Among the simulated sequences, there are 1.4 × 10^5^ unique barcode sequences with different frequencies. To simulate the noise during library preparation for random or Hamming barcodes, CellBarcodeSim can simulate normal, log-normal or exponential distributions, or the user can simulate according to their own uploaded empirical distribution.

#### Barcode labeling simulation

We randomly sampled the barcode lists simulated in the previous step for the corresponding barcode type. We simulated different samples with different total barcode numbers. Each barcode labels one initial cell in the simulation, and those barcode sequences were used as the true barcodes in later precision and recall analysis. We tested scenarios with 300–30,000 initiating cells, but as we found the sequence count distributions to be very similar, as well as the impact of various factors on the precision and recall, we chose values of 150, 300, 600 and 1,200 for the repeat simulations, corresponding to the number of barcodes in most published work.

#### Clonal expansion simulation

We used a log-normal distribution to simulate the final clone sizes of the initially labeled cells. The parameters of the reference distribution are log-mean 1.2 and log s.d. 2, which were chosen based on the experimentally derived mouse naive CD8 T-cell receptor beta-chain sequence clone size distribution described in ref. ^42^ (Supplementary Fig. 20). Observing a log clone size s.d. of ~1 in our VDJ-barcoded mammary gland data, ~2.5 in ref. ^30^ and ~2.5–3 in ref. ^43^, we define alternative scenarios of log clone size s.d. 1 and 3. We used the rlnorm function in R 4.2.1 (ref. ^44^) to generate random numbers and the clone size of each barcode clone was defined by rounding up the nearest integer of the corresponding random number. The CellBarcodeSim tool also offers the power-law clone size distribution.

We note that when the clone size follows a log-normal distribution, the ratio of the 99th quantile, *Q*(0.99), divided by the 1st quantile, *Q*(0.01), depends on only the log s.d. and not on the log-mean (Supplementary Fig. 21), which is explained by the following equations:

1 $$Q\left(q\right)={{\rm{e}}}^{\mu +\sigma \times {\varPhi }^{-1}\left(q\right)}$$

where ${\varPhi }^{-1}\left(q\right)$ is the *q*th quantile of the standard normal distribution with mean, *μ*, and standard deviation, *σ*.

The ratio of the 99th quantile to the 1st quantile:

2 $$Q\left(0.99\right)/Q\left(0.01\right)={{\rm{e}}}^{\mu +\sigma \times {\varPhi }^{-1}\left(0.99\right)}/{{\rm{e}}}^{\mu +\sigma \times {\varPhi }^{-1}\left(0.01\right)}={{\rm{e}}}^{\left\{\sigma \times \left({\varPhi }^{-1}\left(0.99\right)-{\varPhi }^{-1}\left(0.01\right)\right)\right)}$$

Therefore, we can use the range of empirical clone sizes as a quick estimation of log s.d.

#### PCR expansion simulation

The PCR simulation was written in C++ and assumes exponential amplification with an efficiency of 0.703 (ref. ^45^) and an error rate of 1 × 10^−5^ for Taq enzyme, 1 × 10^−6^ for Phusion enzyme and 1 × 10^−7^ for Q5 enzyme. As PCR mutations are rare events, it is unlikely to have more than one mutation per sequence molecule per PCR cycle, and substitution errors are the dominant PCR error type^46^. We therefore allow only a maximum of one base substitution per PCR cycle. In the simulation, we replicated the barcode DNA sequence in silico with the probability of the amplification efficiency, rounding to the nearest natural number, and randomly mutated the base of the newly synthesized sequence with the PCR error rate. To reduce the memory usage, as most of the barcodes have the same sequence due to the low PCR error, we stored barcode sequences in a frequency table of barcode sequences and frequencies. For the new PCR products, the mutant molecular abundance was estimated by multiplying each sequence frequency by the error ratio, considering the sequence length. The value was rounded to the nearest integer. Then uniform random numbers were generated to decide the mutation position and substitution base pair. The sequence frequency table was updated by integrating the mutant sequence. If using UMIs, investigators can select the number of pre-UMI PCR cycles (in which the UMI sequence will not accumulate PCR errors) and the number of post-UMI PCR cycles (when the UMI sequence will accumulate PCR errors). As the PCR primer region is unlikely to have a PCR mutation and this generally corresponds to the barcode flanking regions, by default, the flanking sequence is added after the PCR simulation, matching the sequence to the experimental case when applicable. However, investigators have the option to include the flanking region in the PCR simulation by appending the fixed flanking regions to the barcodes when simulating the barcode library (see Supplementary Vignette 1 for more detail).

#### Sequencing simulation

Sequencing simulation was conducted using the ART (version 2016-06-05) command line tool (an NGS reads simulator), which supports base substitution, insertions and deletions^39^. The ART-integrated MiSeq V1 and HiSeq 2000 read error profiles (learnt empirically from relevant training data^39^) were used to generate single-end sequencing with 100 base pairs, with other parameters as default. We describe the sequencing profiles used in Supplementary Fig. 22a,b, together with PCR error in Supplementary Fig. 22c. When comparing the barcode clone size distributions between different simulated datasets, we sample 10^5^ sequencing reads to make the distributions easier to compare.

### Simulating VDJ-barcoded data with high clone size variation and UMIs

We simulated VDJ barcode sequencing with UMIs for high clone size variation samples (details of the parameters in Supplementary Table 3). With an expected sequencing depth of 50 reads per UMI, we filtered out UMIs that have read <10 (based on sensitivity analysis to identify when the number of barcodes detected plateaus) and then varied the UMI count threshold to compute the PR AUC.

### DNA cellular barcode pre-processing strategy evaluation

#### Evaluation of filtering strategies precision, recall and AUC

In the simulation study, we evaluated filtering strategies using precision and recall. The precision and recall are defined as:

3 $${\rm{Precision}}={n}_{{\rm{true}}}/{n}_{{\rm{output}}}$$

4 $${\rm{Recall}}={n}_{{\rm{true}}}/{n}_{{\rm{input}}}$$

where *n*_input_ is the number of barcodes used for labeling, *n*_output_ is the total number of barcodes in the pre-processing output, and *n*_true_ is the number of barcodes shared between the pre-processing output and the barcodes used for labeling.

The precision and recall depend on the threshold used for barcode filtering. PR curves were drawn using a range of read count thresholds (or UMI count in the UMI cleaning case), and the AUC was calculated to evaluate the overall goodness of a filtering strategy. The AUC is a way to evaluate the goodness of a method regardless of threshold and was computed using the ROCR R package^47^.

All boxplots depict 25th, 50th and 75th percentiles in the box, 25th or 75th percentile minus or plus 1.5 × interquartile range (IQR), respectively, for the whiskers, and points show outliers beyond the whiskers.

### Barcode filtering

We enabled four barcode filtering strategies in the CellBarcode package with bc_cure_umi, bc_cure_clustering, bc_cure_depth and bc_auto_cutoff functions. They are (1) read count thresholding filtering with bc_cure_depth function, (2) reference library filtering, (3) cluster filtering and (4) UMI filtering.

Read count threshold filtering excludes the barcodes with read counts under the threshold. The automatic threshold function determines the threshold by applying one-dimensional weighted *k*-means clustering to the barcode read count distribution. It involves the following steps. (1) Remove barcodes with count below the median (as there are generally many more spurious than true barcodes). (2) Transform counts by log2(*x* + 1). (3) Apply one-dimensional *k*-means clustering^48^ to the transformed read counts with cluster number fixed at 2 and with weights of the transformed count. (4) Use the boundary between the two clusters as the read count threshold.

In reference library filtering, only barcodes appearing in the barcode reference list are retained in the final output, and all others are filtered out. In the simulations, the barcode reference library was the barcode list generated in ‘Lineage barcode simulation’.

For cluster filtering, we assumed that with a low-error-rate, spurious error barcodes should have a much lower read number compared with their true ‘mother’ sequences. We clustered barcodes with similar sequences to identify potential ‘mother’ and ‘daughter’ sequence pairs. Then we removed the ‘daughter’ sequences, thus making it easier to identify true barcodes with small clone size. We used the following clustering process for each sample. (1) Identify the most abundant barcode based on read counts. (2) Compute the distance (Hamming distance or Levenshtein distance) between the most abundant barcode and the other barcodes, starting from the least abundant barcode. (3) If the distance between two barcodes is below a set threshold, and the reads count fold change between them is above a set threshold, the less abundant barcode is removed. (4) Iterate for each of the other barcodes in order of abundance. The process is described by the pseudo code in Supplementary Algorithm 1.

UMI filtering takes advantage of the UMI sequence. The default in CellBarcode is to assume that UMIs are not unique in line with the findings of ref. ^49^ (although the reader has the option to assume the converse if they wish). We first counted the number of reads for each UMI–barcode combination and then applied a read count threshold. The remaining barcode abundances were quantified by summing the UMI count. We assume that the probability of an error in both the UMI and its associated barcode sequence is very low, and so we do not cluster similar UMIs. This may result in a slight overestimation of clone size if a UMI sequence results from an error, but should not affect barcode identification.

### Benchmarking CellBarcode and genBaRcode

To compare the output and runtime of CellBarcode (version 1.7.1) and genBaRcode (with version 1.2.6), we simulated a random barcode dataset using the method described above with parameters (1) 300 cells induced, (2) log-normal clone size distribution with log clone size s.d. of 2 and log clone size mean 1.2, (3) 30 PCR cycles, 1 × 10^−6^ PCR mutation rate, PCR efficiency 0.705, and (4) HiSeq 2000 100-bp sequencing error profile.

For barcode extraction, the regular expression AAAAAAAAAAGGGGG([ATCG]{14})ATCGATCGTTTTTTT was used in CellBarcode to extract the 14-bp random barcode, and the pattern AAAAAAAAAAGGGGGNNNNNNNNNNNNNNATCGATCGTTTTTTT was used in genBaRcode. Then at the barcode filtering step, the clustering strategy was used, which removed the minority barcodes with a Hamming distance of 1 to the majority ones. We note that CellBarcode discards error reads, whereas genBaRcode adds them to the majority one. We chose this strategy as we found that the resulting underestimation of clone size due to discarding clustered reads was very slight (see comparison of genBaRcode and CellBarcode, Supplementary Figs. 1 and 16), whereas if a clustered barcode is actually a real barcode, for example, when library edit distance is small, the result could be a substantial overestimation of some clone sizes. For further information on how this clustering process was carried out, please refer to the ‘Barcode filtering’ section. The runtime of above analysis was evaluated by Sys.time function in R 4.2.1 (ref. ^44^). We used CellBarcode version 1.7.1 and genBaRcode version 1.2.6 here and throughout.

### Acquisition, analysis and simulation of experimental data

Several datasets are analyzed in this paper; below, for each, we describe first the experimental dataset, then the barcode analysis and finally the simulation parameters (for bulk data).

### Lentiviral barcode dataset

#### Experimental data

We used a lentiviral barcode dataset from our previous publication^30^. Briefly, it consists of 13,564 myeloid cells recovered from mice 4 weeks after transplantation of barcoded erythropoietin (EPO)-treated haematopoietic stem and progenitor cells (HSPCs). The HSPCs were labeled by the LG2.2 barcode library, which has a 20-bp fixed-length barcode region, a diversity of >10,000 barcodes and has a reference library. The myeloid cell DNA was divided into two technical replicates before PCR amplification and sequencing.

#### Barcode analysis

The output FASTQ file from ref. ^30^ was analyzed with the CellBarcode package using the regular expression ACGGAATGCTAGAACACTCGAGATCAG(.{20})ATGTGGTATGATGTATC to extract the 20-bp barcode sequence between constant regions. In the regular expression, the first bases ACGGAATG are the plate index used to demultiplex samples with the same P7 index. The extracted barcodes were cleaned by reference library or cluster filtering separately. For the cluster filtering, we remove the minority barcodes with Hamming distance 1 to the majority ones as the barcode library has a minimum edit distance of 5 (Supplementary Fig. 11c). Then we normalized the read number (${n}_{i}^{{{\mathrm{reads}}}}$) by the total cell count (${n}_{{{\mathrm{total}}}}^{{{\mathrm{cell}}}}$) to estimate the clone size (${n}_{i}^{{{\mathrm{cell}}}}$) for each barcode clone (*i*) with following formula:

5 $${n}_{i}^{{{\mathrm{cell}}}}={n}_{i}^{{{\mathrm{reads}}}}/{\sum }_{i}{n}_{i}^{{{\mathrm{reads}}}}\times {n}_{{{\mathrm{total}}}}^{{{\mathrm{cell}}}}$$

For comparing CellBarcode and genBarcode on the fixed-length barcode dataset from ref. ^30^, both methods use the same criteria to extract and filter barcodes, which involves defining a barcode as a 20-bp random sequence between fixed sequences ACGGAATGCTAGAACACTCGAGATCAG and ATGTGGTATGATGTATC. In addition, cluster filtering is performed to remove minority barcodes with a Hamming distance of 1 and the runtime was measured by the ‘Sys.time()’ function in R. The Spearman correlation was performed using all barcodes.

Bartender can define only a fixed region of 5 bp. Therefore, the barcode definition is set as a 20-bp random sequence between ATCAG and ATGTG. The default Bartender clustering filtering has been applied. The runtime was measured by the ‘time’ function in the shell. The Bartender version used here (and following references) is https://github.com/LaoZZZZZ/bartender-1.1/commit/9683af760cc33f31185140957d503af7f3e230be.

#### Simulation

To simulate the barcodes, we used a lentiviral barcode reference library to label 15 cells. The labeled cells were then subjected to clonal expansion, following a log-normal distribution with a mean log clone size of 1.2 and s.d. of 3. After performing 30 PCR cycles with an error rate of 1 × 10^−6^, we concatenated the constant regions: 5′ ACGGAATGCTAGAACACTCGAGATCAG and 3′ ATGTGGTATGATGTATCA. Finally, we simulated the sequencing using the HiSeq 2000 profile, aiming for 50 reads per cell.

### CRISPR gRNA dataset

#### Experimental data

Tumor organoids were derived from Apc1638N mice^50^ and transduced with lentiviral particles expressing the Cas9 enzyme along with blasticidin resistance (Addgene plasmid 52962) as described previously^51^. Selection of infected organoids was achieved by adding 10 g ml^−1^ blasticidin (A1113903 Thermo Fisher) to the medium.

Cas9-expressing tumor organoids were then transduced with lentiviral particles each containing a single guide RNA sequence derived from a bank of 1,796 single guide RNAs that target Notch1-related genes, as found in ref. ^52^. Transduced organoids were collected either at 48 hours or at 7 days post infection. At 7 days, organoids were dissociated and Tomato-expressing live cells (based on DAPI exclusion) were fluorescence-activated cell sorted (FACS) (Supplementary Fig. 24). DNA was extracted using a standard phenol:chloroform:isoamyl alcohol protocol. Briefly, cells were resuspended in 500 µl PBS and 1 ml phenol:chloroform:isoamyl alcohol (25:24:1) solution (Sigma P2069) was added. After centrifugation at 16,000*g* for 5 min, the aqueous phase was collected and one volume of chloroform (Sigma 32211) was added. Following a vortex homogenization step, the samples were centrifuged at 16,000*g* for 5 min and the aqueous phase was recovered. Precipitation of the DNA was then performed by adding 1 µl glycogen at 20 µg µl^−1^ (Thermo Fisher 10814010), 0.5 volume of the sample of 5.5 M sodium acetate and 2.5 volumes of the sample of cold 100% ethanol. After overnight at −20 °C and 30 min of centrifugation at 16,000*g* at 4 °C, the precipitated DNA pellet was recovered in 30 µl water and quantified by nanodrop. Ten microlitres of DNA were then amplified by PCR in triplicates for each sample to add P5-staggers and P7-index oligos to perform NGS DNA sequencing. The PCR was performed with Taq polymerase (Promega M7406) for 22 cycles (30 s at 95 °C, 30 s at 53 °C, 30 s at 72 °C).

The sequences of the primers are the following:

P5 staggers:

5′AATGATACGGCGACCACCGAGATCTACACTCTTTCCCTACACGACGCTCTTCCGATCT[s]TTGTGGAAAGGACGAAACACCG)

P7 index:

(5′CAAGCAGAAGACGGCATACGAGATNNNNNNNNGTGACTGGAGTTCAGACGTGTGCTCTTCCGATCTTCTACTATTCTTTCCCCTGCACTGT)

Bead purification of the PCR product using a ratio of 1.2 was performed following the manufacturer’s protocol (Beckman Coulter B23318). Quality and concentration of the samples were assessed on a Tapestation. Then it was sequenced by MiSeq SE110 with 10% PhiX.

#### Barcode analysis

The gRNA sequencing results are processed by CellBarcode with regular expression ‘AAGGACGAAACACCG(.{20})’. After reference library-based filtering, the log clone size s.d. was calculated.

#### Simulation

We simulated the gRNA sequencing data using a barcode library consisting of 1,796 gRNA sequences. The simulated cells were labeled with a clone size log-mean of 1, but varying log clone size s.d. values ranging from 0.5 to 2.5. To mimic the error rate of Taq polymerase, we performed 20 PCR cycles with a PCR error rate of 10^−4^. Finally, the sequencing was simulated using the built-in ART MiSeq profile. We analyzed the simulated results in the same manner as the experimental dataset.

### VDJ barcode MEF cell line dataset

#### Experimental data

The VDJ barcodes are produced by an inducible mouse in situ barcode system based on VDJ recombination^20^. In this system, the V, D and J sequences are separated by the signal cassettes, which are recognized and cut out by the Rag1 (recombination activating gene-1) and Rag2 (recombination activating gene-2) enzymes and repaired by non-homologous end joining repair, which is error prone, creating the diversity of the final barcode sequences. A cassette with reversed *Rag1*, *Rag2* and *TdT* (terminal deoxynucleotidyl transferase) genes are surrounded by LoxP sequences, which can be activated by Cre floxing. The TdT adds de novo nucleotides to the end joins, which increases the diversity of the final barcode sequence.

In ref. ^20^, MEF cell lines were created from individual cells of a VDJ barcode-induced mouse with known unique barcode sequences. There are a total of 7 MEF cell lines with barcode sequences: CTCGAGGTCATCGAAGTATCAAGTCCAGTTCTACTATCGTAGCTACTA, CTCGAGGTCATCGAAGTATCAAGTCCAGTACTATCGTACTA, CTCGAGGTCATCGAAGTATCAAGTCCAGTCTACTATCGTTACGACAGCTACTA, CTCGAGGTCATCGAAGTATCAAGTCCAGTTCTACTATCGTTACGAGCTACTA, CTCGAGGTCATCGAAGTATCAAGTCCATCGTAGCTACTA, CTCGAGGTCATCGAAGTATCAAGTCCAGTACTGTAGCTACTA and CTCGAGGTCATCGAAGTATCAAGTCCAGTATCGTTACGCTACTA.

These cell lines were mixed in specific ratios, in ascending order of powers of 2 from 1 to 7. Sequencing data were then generated with different numbers of initiating cells^20^.

#### Barcode analysis

We re-analyzed one of the technical replicates of +0, +2, +4 and +6 PCR cycles with CellBarcode using the regular expression ([ACGT]{12})CTCGAGGTCATCGAAGTATC([ACGT]+)CCGTAGCAAGCTCGAGAGTAGACCTACT to capture the variable-length barcode between the fixed regions of CTCGAGGTCATCGAAGTATC and CCGTAGCAAGCTCGAGAGTAGACCTACT, after a 12-bp random UMI.

#### Simulation

For Fig. 2b, to simulate a MEF cell line experiment, we simulated 6,250 cells (half of the 12,500 cells to mimic the technical replicates) with barcode sequences and clone sizes that match the experimental set-up. After two cycles of preamplification, a 12-bp random UMI is added with a tagging efficiency of 2%. This is followed by 30 cycles of PCR amplification, with a PCR efficiency of 0.705 and a PCR error rate of 1 × 10^−5^.

For Supplementary Fig. 7, we simulated the full dataset to mimic the experiment described above with different numbers of PCR cycles. We used the same barcode sequences, cell number and type of sequencing while incorporating variable total PCR cycles of +0, +2, +4 and +6. The same fixed 3′ sequence as the experimental dataset was added (CCGTAGCAAGCTCGAGAGTAGACCTACTGGAATCAGACCGCCACCATGGTGAGCA), and the simulated data were analyzed in the same manner as the experimental dataset.

### In vivo VDJ barcode mammary gland dataset

#### Experimental data

The VDJ barcode mouse was crossed with a Notch1Cre^ERT2^ mouse^53^. Lactating mothers were injected with tamoxifen (0.1 mg per g of mouse body mice, MP Biomedicals, 156738) as described^54^ to induce Cre recombination in the progeny at stage P0. Mammary tissue of a DRAG^+/−^ Notch1Cre^ERT2+/−^ female was then collected at 6 weeks of age and mammary single-cell dissociation was performed as previously described^55^. Briefly, mammary fat pads were mechanically minced with scissors and scalpel and digested for 90 min at 37 °C in CO_2_-independent medium (Invitrogen, 18045-054) supplemented with 5% fetal bovine serum, 3 mg ml^−1^ collagenase A (Roche, 0103586001) and 100 U ml^−1^ hyaluronidase (Sigma, H3884). The resulting suspension was sequentially resuspended in 0.25% trypsin–EDTA for 1 min, and then 5 min in 5 mg ml^−1^ dispase (Roche, 04942078001) with 0.1 mg ml^−1^ DNase I (Sigma, D4527) followed by filtration through a 40 μm mesh. Red blood cells were lysed in NH_4_Cl. The obtained single-cell suspension was then stained with the following Biolegend antibodies, at a 1/100 dilution: APC anti-mouse CD31 (102510), APC anti-mouse Ter119 (116212), APC anti-mouse CD45 (103112), APC/Cy7 anti-mouse CD49f (313628) and PE anti-mouse EpCAM (118206). Dead cells (DAPI^+^) and CD45^+^/CD31^+^/Ter119^+^ (Lin^+^) non-epithelial cells were excluded before analysis using a FACS ARIA flow cytometer (Becton Dickinson) (Supplementary Fig. 25). In total, 20,589 barcoded GFP^+^, Lin^−^, EpCAM^high^ and CD49f^low^ luminal cells were sorted into the lysis buffer (Viagen, 301-C).

For these data, we have access to both UMI and non-UMI sequencing libraries as each technical replicate was split in two and processed in parallel with UMI and non-UMI protocols.

For the UMI barcode sequencing library, we follow the protocol described in ref. ^20^. In brief, the lysed cells were sheared by sonication then divided into two technical replicates, and the target region captured by beads. The DNA in beads was used as a template to do the preamp PCR to amplify the target region with 11 cycles. Next, the UMI was introduced by a second PCR, then the third PCR to add the M1 sequences, and finally the fourth PCR to add the adapter sequence to get the sequencing library. The library was sequenced by MiSeq SE110 with 10% PhiX.

For the non-UMI barcode sequencing library, the preamp PCR product from the UMI barcode library was used to generate a non-UMI sequencing library. We took 100 μl preamp PCR product, cleaned it with 1.8× SPRI beads and eluted in 30 μl DNAse-free water. The first PCR used 28 μl of the eluted DNA as template with 50 μl PCR reaction (10 μl 5× Q5 buffer, 0.5 μl 2 U μl^−1^ Q5 DNA polymerase, 1 μl 10 mM dNTP, 0.25 μl 100 μM preamp Fwd primer and preamp Rev primer, 10 μl DNAse-free water) for 19 cycles (98 °C 2 min; 19 cycles of 98 °C 10 s, 67 °C 30 s, 72 °C 30 s; then 72 °C 5 min). Then the products were cleaned by 1.8× SPRI beads and eluted into 30 μl DNAse-free water. The second PCR used 15 μl of the eluted DNA from last step as the template with 50 μl reaction (10 μl 5× Q5 buffer, 1 μl 2 U μl^−1^ Q5 DNA polymerase, 1 μl 10 mM dNTP, 0.25 μl 100 μM preamp Fwd primer and M1 Rev primer, and 22.5 μl DNAse-free water) for 5 PCR cycles (98 °C 2 min; 5 cycles of 98 °C 10 s, 67 °C 30 s, 72 °C 30 s; then 72 °C 5 min). After that, the PCR products were cleaned by 1.8× SPRI beads and eluted into 30 μl DNAse-free water. The third PCR used 10 μl DNA from last step as template to add the NGS adapters by 20 μl PCR reaction (4 μl 5× Q5 buffer, 0.4 μl 2 U μl^−1^ Q5 DNA polymerase, 0.4 μl 10 mM dNTP, 0.1 μl 100 μM P5 tagging primer, 4 μl 2.5 μM P7 tagging primer with index and 1.1 μl DNAse-free water) by 5 PCR cycles (98 °C 2 min; 5 cycles of 98 °C 10 s, 67 °C 30 s, 72 °C 30 s; then 72 °C 5 min). The final DNA was cleaned by 1× SPRI beads, and eluted into 30 μl DNAse-free water. The library with 10% PhiX was sequenced by MiSeq in SE110 mode with a 25M sequencing chip aimed for 20M reads output. This library was sequenced together with other samples but independent to the UMI barcode library.

preamp Fwd primer:

ACTCACTATAGGGAGACGCGTGTTACC

preamp Rev primer:

GACACGCTGAACTTGTGGCCGTTTA

M1 Rev primer:

AGTTCAGACGTGTGCTCTTCCGATCCAGCTCGACCAGGATGGG

P5 tagging primer:

AATGATACGGCGACCACCGAGATCTACACTCTTTCCCTACACGACGCTCTTCCGATCTACTCACTATAGGGAGACGCGTGTT

P7 tagging primer:

CAAGCAGAAGACGGCATACGAGATTGACTGAGTGACTGGAGTTCAGACGTGTGCTCTTCCGATC

#### Barcode analysis

For the VDJ barcode UMI library analysis using CellBarcode, we extract the barcode and UMI using regular expression ‘(.{16})CCTCGAGGTCATCGAAGTATCAAG(.*)CCGTAGCAAGCTCGAGAGTAGACCTACT’, which defines the 16-bp UMI sequence before the constant region and the variable-length VDJ barcode sequence followed by another constant region. Then we removed the UMI barcode tags with fewer than 100 reads and counted the UMIs per barcode in the remaining tags, which is a robust threshold as the final barcode is very stable when we increase the threshold (Supplementary Fig. 18a), and used the remaining barcodes. Investigators can use a similar approach to determine a read count threshold, in conjunction with knowledge about their targeted sequencing depth per cell.

For the non-UMI barcode library sequencing, we used the regular expression ‘CCTCGAGGTCATCGAAGTATCAAG(.*)CCGTAGCAAGCTCGAGAGTAGACCTACT’ to match the variable-length VDJ barcode between the constant regions. The automatic read count threshold was used to identify true barcodes.

We compared CellBarcode and Bartender using both UMI and non-UMI sequencing described above. We extracted the barcodes with CellBarcode as described above, with the UMI tag requiring a minimum of 100 reads to be counted. In non-UMI libraries, an automatic threshold is applied in CellBarcode. For Bartender, it only allows a maximum of a 5-bp match in the fixed region. Therefore, the barcode is defined between the fixed regions TCAAG and CCGTA. The UMI is defined by the first 16-bp random sequence in both cases. Then the clustering with one mismatch is used for both UMI and non-UMI sequencing. The runtime of Bartender is measured by shell command ‘time’, and for CellBarcode by the ‘Sys.time()’ function in R. The shared barcodes were counted and visualized using a Venn plot. Linear regression was performed on the shared barcodes.

#### Simulation

We used CellBarcodeSim to simulate the above VDJ sequencing data. The simulation included a VDJ barcode library with 100 cells, which were expanded using a log-normal distribution (log clone size mean 1.2, s.d. 1). We used a random UMI of length 16 bp, and sequenced 100 reads per UMI using the ART built-in MiSeq profile, resulting in sequences of length 111 bp. In addition, we added fixed regions at the 5′ end (CCTCGAGGTCATCGAAGTATCAAG) and the 3′ end (CCGTAGCAAGCTCGAGAGTAGACCTACTGGAATCAGACCGCCACCATGGTGAGCACACGTCTGAACTCCAGTCACTCAGTCAATCTCGTATGCCGTCTTCTGCTTG). Other parameters were kept default.

### CellTag barcode scRNA-seq dataset

#### Experimental data

The scRNA-seq CellTag BAM file^36^ was downloaded from the Sequence Read Archive with accession number SRR7347033. This file corresponds to the MEF cell line that was infected with CellTag barcodes, underwent fate reprogramming through overexpression of transcription factors FOXA1 and HNF4α, and was sequenced after 15 days.

#### Barcode analysis

For the CellTagR analysis, we followed its demo described here https://github.com/morris-lab/CellTagR. First, we filtered the BAM file in bash by (1) filtering unmapped reads and (2) filtering transgene reads. The filtered BAM file was used as input to both the CellTagR and CellBarcode pipelines. After first creating a CellTag object, the V1 barcode was extracted from the BAM file, by matching 5′ constant GGT and 3′ constant GAATTC. After that, barcode filtering was applied including: (1) filter cells (a list of cells passing quality control was downloaded from the Gene Expression Omnibus (GEO) with dataset ID GSE99915), (2) barcode sequence error correction with clustering using Starcode, (3) keep UMIs with at least 2 reads and (4) barcode reference library filtering (whitelist filtering). The barcode reference library (whitelist) can be found with the demo datasets of the CellTagR package. Barcode clustering error correction was done by starcode-1.4 (ref. ^56^).

We applied the CellTagR pipeline described above as closely as possible using CellBarcode. Using CellBarcode, we extracted the V1 barcode using the regular expression ‘GT([ATCG]{8})GAATTC’, which matches the 8-bp DNA sequence surrounded by two fixed constant regions. Then, we carried out the four filtering steps using the CellBarcode package, which are (1) filter cells using the quality control passed list described above, (2) barcode sequencing correction by removing minority barcodes with a Hamming distance of 1 to the majority one, (3) keep UMI with at least 2 reads and (4) barcode reference library filtering.

### VDJ barcode scRNA-seq dataset

#### Barcode analysis

In this section, we describe VDJ barcode extraction with CellBarcode, the barcode filtering was described in ‘Results’.

In single-cell sequencing data analysis, each cell is stored as an individual sample in the BarcodeObj, and this object has the same data structure as that of bulk analysis.

The FASTQ file was acquired from the authors. Their read 1 and read 2 were concatenated. In the sequence, we defined the cellular 10x barcode as the first 16 bases, and the UMI as 12 bases followed, according to the 10 × 3′ scRNA-seq reads structure. The lineage barcode sequence was extracted using the 3′ and 5′ constant sequences: ‘CGAAGTATCAAG’ and ‘CCGTAGCAAG’.

The result in original paper was accessed from GitHub: https://github.com/TeamPerie/Cosgrove-et-al-2022/blob/main/Figure1/RNA_BC_PREPROCESSING/input_output_m534/agrep_10xbc_and_vbc_m534_both.txt.gz. A brief description of the barcode filtering of the original is as follows: UMIs were filtered to keep only those with 3 or more reads and one dominant VDJ barcode (defined as ≥0.45 reads). The dominant barcode for each UMI was extracted, and finally they assigned one VDJ barcode to a 10x cell if there is good agreement across UMIs, defined as ≥0.75 agreement across all remaining UMIs. If there is only one UMI retained, they further ensured that the VDJ barcode for this UMI was the dominant barcode across all the reads for that cell and has ≥0.45 of reads.

### Statistics and reproducibility

No statistical method was used to predetermine sample size. No data were excluded from the analyses. The experiments were not randomized. The investigators were not blinded to allocation during experiments and outcome assessment.

### Reporting summary

Further information on research design is available in the Nature Portfolio Reporting Summary linked to this article.

### Supplementary information

Supplementary Information Supplementary Tables 1–3, Figs. 1–25, Vignettes 1 and 2, and Algorithm 1.

Reporting Summary

Supplementary Data 1 Parameters of the Bayesian network model inferred by IGoR from VDJ barcode data in mouse mammary gland tissue, then used to generate VDJ barcodes for the simulation study.

Supplementary Data 2 Marginals of the Bayesian network model inferred by IGoR from VDJ barcode data in mouse mammary gland tissue, then used to generate VDJ barcodes for the simulation study.

### Source data

Source Data Fig. 2 Statistical source data.

Source Data Fig. 3 Statistical source data.

Source Data Fig. 4 Statistical source data.

Source Data Fig. 5 Statistical source data.

Source Data Fig. 6 Statistical source data.

## Data Availability

The lentiviral barcodes dataset from ref. ^30^ was obtained from ref. ^57^; the corresponding pre-analysed data are available on GitHub (https://github.com/TeamPerie/Eisele-et-al.). The CellTag barcode sequencing data from ref. ^36^ are on GEO with dataset ID GSE99915. The ref. ^17^ barcoded scRNA-seq dataset is on GEO with dataset ID GSE164716. The mammary gland VDJ barcode dataset and gRNA sequencing data are available on Zenodo (10.5281/zenodo.8124948)^58^. The MEF cell line mixes VDJ barcode dataset is available on Zenodo (10.5281/zenodo.10027001)^59^. The VDJ-barcoded scRNA-seq data from ref. ^35^ belongs to the authors of that paper and was given to us for the purposes of this paper; to obtain this data, please contact L.P. Source data are provided with this paper.

## Code availability

Code for all analysis in this study is available on GitHub (https://github.com/TeamPerie/CellBarcode_paper_Sun_et_al) and on Zenodo (10.5281/zenodo.10492761)^60^. The CellBarcode package is available on Bioconductor (https://bioconductor.org/packages/release/bioc/html/CellBarcode.html and 10.18129/B9.bioc.CellBarcode)^61^. The Barcode sequencing simulation kit is available on GitHub (https://github.com/TeamPerie/CellBarcodeSim) and on Zenodo (10.5281/zenodo.10492831)^62^.
