# Supplementary material for: Extracting, filtering and simulating cellular barcodes using CellBarcode tools
Source: Nat Comput Sci. 2024 Feb 19;4(2):128–43. doi: 10.1038/s43588-024-00595-7 (PMC10899113; doi:10.1038/s43588-024-00595-7)
Supplement: Supplementary file 1 — Supplementary Tables 1–3, Figs. 1–25, Vignettes 1 and 2, and Algorithm 1. [file 43588_2024_595_MOESM1_ESM.pdf]

nature computational science

Resource

https://doi.org/10.1038/s43588-024-00595-7

Extracting, filtering and simulating cellular barcodes using CellBarcode tools

In the format provided by the authors and unedited

Page 2—Supplementary Tables 1-3 and Figures 1-25

Page 31—Supplementary Vignettes 1

Page 60—Supplementary Vignettes 2

Page 70—Supplementary Algorithm 1

Supplementary Table1. Cellular barcode pre-processing tools comparison

Supplementary table2. Non-UMI barcode sequencing simulation parameters

Supplementary table3. UMI barcode sequencing simulation parameters

Supplementary Figure 1. Comparing the performance of CellBarcode and genBaRcode on a simulated dataset.

Supplementary Figure 2. Example of a Precision-Recall curve.

Supplementary Figure 3. Read numbers for spurious sequences and true barcodes.

Supplementary Figure 4. AUC of precision-recall curve after reads count threshold filtering.

Supplementary Figure 5. Precision and recall for different values of threshold.

Supplementary Figure 6. Precision and recall for different values of threshold.

Supplementary Figure 7. Read frequency distributions for CRISPR gRNA experiments

Supplementary Figure 8. Sequencing of VDJ-barcoded MEF data with different PCR cycle numbers.

Supplementary Figure 9. Reads number of spurious sequences and true barcodes after reference library filtering.

Supplementary Figure 10. AUC of precision-recall curve after reference library filtering.

Supplementary Figure 11. Edit distance distribution for simulated VDJ and random barcodes.

Supplementary Figure 12. Precision and recall for different threshold values after reference library filtering.

Supplementary Figure 13. AUC of precision-recall curve after cluster filtering.

Supplementary Figure 14. Read numbers of spurious sequences and true barcodes after cluster filtering.

Supplementary Figure 15. Comparing CellBarcode with genBaRcode and Bartender using lentiviral barcoding data.

Supplementary Figure 16. UMI read count threshold optimization and comparing CellBarcode with Bartender using the in vivo VDJ barcode sequencing.

Supplementary Figure 17. Processing CellTag barcoded scRNA-seq data using CellBarcode.

Supplementary Figure 18. Comparing scRNA-seq barcode extraction using CellBarcode with the original results in Cosgrove et al.

Figure Supplementary 19. Analysis of Marsolier et al 2022 barcoded scRNA-seq data using CellBarcode.

Figure Supplementary 20. Clone size distribution simulation.

Supplementary Figure 21. Log clone size SD and clone size range.

Supplementary Figure 22. Profiling NGS Sequencing error and PCR error.

Supplementary Figure 23. Precision and recall for different threshold values after cluster filtering.

Supplementary 24. Cell sorting strategy used to obtain gRNA infected cells.

Supplementary 25. Cell sorting strategy used to obtain Luminal barcoded cells.

Features	genBaRcode	Bartender	CellBarcode	CellTagR
**Barcode Type**	Fixed length barcode	Variable length Max 5bp for fix region	Variable length	CellTag System (Fix length barcode)
**Automatic reads count threshold**	NO	NO	YES	-
**Handle UMI**	NO	YES	YES	YES
**Subset operation**	Partial	NO	YES	-
**scRNASeq barcodes detection**	NO	NO	YES	YES
**Fastq QC and Filtering**	YES	NO	YES	-
**Fastq process Speed**	1 X	5 X Faster	20 X Faster	-
**Barcode clustering**	YES	YES	YES	-
**Barcode clustering Speed**	1X	20 X Faster	70 X Faster	-
**Package distribution**	YES	NO	YES	YES
**Barcode Reference library**	YES	NO	YES	YES

Supplementary Table1. Cellular barcode pre-processing tools comparison

Comparison of the features of four DNA cellular barcode analysis tools: genBaRcode, Bartender, CellTagR, and the CellBarcode tool introduced by us. The Fastq processing speed and Barcode clustering speed were evaluated using a simulated dataset on a personal computer. The run time was measured and the speed was estimated, then rounded to the nearest multiple of 5 compared to genBarCode.

Parameter Name	Default value	Variant value	Note
Barcode Type	Random	Hamming distance, VDJ	Hamming distance barcode is generated by R package DNABarcodes, and VDJ barcode is simulated by IGoR
Clone Number	300	150, 600, 1200	The number of cells labeled by barcode
Log clone size sd	2	1, 3	Different tissue have different clone size variation, the default 2 was derived from TCR clone size
Log clone size Mean	1.2	0.6, 3	The default value 1.2 is from TCR clone size
PCR cycle	30	20, 40	Normally, PCR products reache plateau by 40
PCR efficiency	0.705	0.5, 0.9	PCR efficiency 0.703 is from Nucleic Acids Research, 1997, Vol. 25, No. 15
PCR error	1E-06	1E-5, 1E-7	PCR error: 1e-6 for Phusion;1e-5 for Taq; 1e-7 for Q5
Barcode length	14	10, (0-70 for VDJ barcode)	VDJ barcode has flexible length
Reads per cell	50	25, 100, 1000	In our previous project, we used 50-100 reads per cells
NGS profile	HiSeq2000	MiSeq V1	We used the built in profile of the ART simulator

Supplementary table2. Non-UMI barcode sequencing simulation parameters

We simulated non-UMI barcode sequencing data using the parameter values in the table. We used the default condition as a reference and changed one parameter at a time to assess the sensitivity of the results to each parameter.

Parameter Name	Default value	Variant value
Barcode Type	VDJ	-
Clone Number	300	-
Log clone size SD	2	3
Log clone size Mean	1.2	-
Total PCR cycle	30	-
Preamp PCR cycle	10	-
PCR efficiency	0.705	-
PCR error	1E-06	-
Barcode length	0-70	-
UMI length	8	-
UMI tagging efficiency	0.02	-
Reads per UMI	50	-
NGS profile	HiSeq2000	-

Supplementary table3. UMI barcode sequencing simulation parameters

The table displays the parameter values used to simulate UMI barcode sequencing data. The default values, available in Supplementary Table 2, were kept unchanged except for the new parameters: preamp PCR cycle, UMI length, UMI tagging efficiency, and Reads per UMI. To examine the situation of significant clone size variation, we employed a log clone size standard deviation of 3 (natural logarithm).

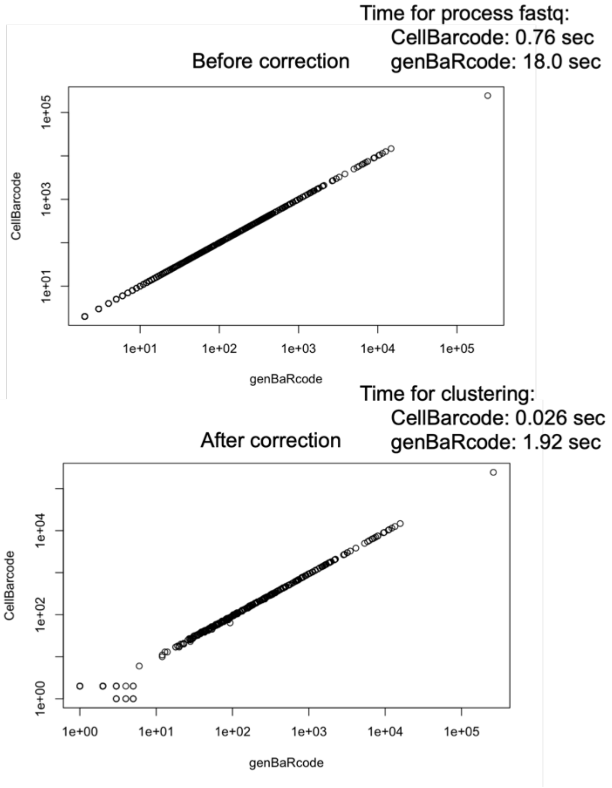

Supplementary Figure 1. Comparing the performance of CellBarcode and genBaRcode on a simulated dataset.

A simulated barcode sequencing sample was analyzed by CellBarcode and genBaRcode separately. The reads per barcode before and after filtering obtained with genBaRcode were plotted against the ones obtained with CellBarcode. In this plot, each dot is a barcode and the axes were log10-transformed. The run time (evaluated by Sys.time) to read the FASTQ file and cluster filter the data for both packages are noted in the top right corner of the figure. For the simulated barcode dataset, we generated a random barcode dataset with the following parameters: 1) 300 induced barcode clones; 2) Log-normal distribution of clone sizes, with a mean of 1.2 and standard deviation of 2; 3) PCR amplification consisting of 30 cycles, with a mutation rate of 1e-6 and an efficiency of 0.705; and finally, sequencing was performed using HiSeq2000 technology with a profile that introduced errors in reads up to lengths of 100bp. Cluster filtering was applied with both packages, barcodes with a Hamming distance value of 1 to more abundant barcodes were removed.

Supp Figure4. Precision-Recall curve by the reads count cutoff point

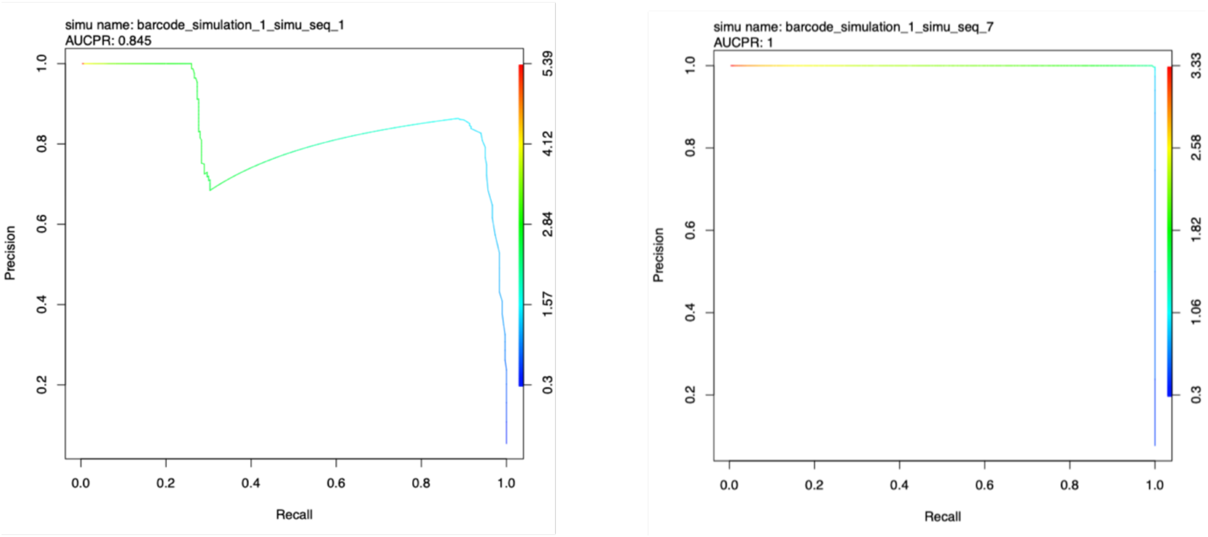

Supplementary Figure 2. Example of a Precision-Recall curve.

The Precision-Recall curve generated on a non-UMI barcode simulation sample with default parameter values with CellBarcodeSim. The recall was plotted against precision. The recall is defined as the proportion of all true barcodes found in the output and the precision is the proportion of output barcodes which are true. The colors correspond to the log10 read count threshold filter applied. The area under the curve is 0.845 in this case.

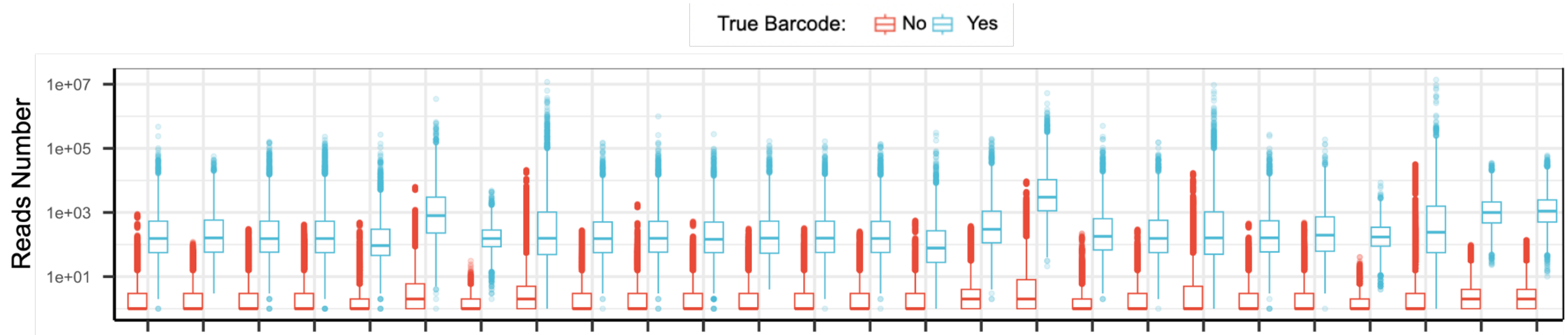

Barcode Type	Random	-	-	-	-	-	-	-	-	-	-	-	-	-	-	-	-	-	Hammin	Hammin	-	VDJ	VDJ	VDJ	VDJ	VDJ
Clone Number	300	150	600	1200	-	-	-	-	-	-	-	-	-	-	-	-	-	-	-	-	-	-	-	-	600	1200
Log clone size SD	2	-	-	-	-	-	1	3	-	-	-	-	-	-	-	-	-	-	-	3	-	-	1	3	1	1
Log clone size Mean	1.2	-	-	-	0.6	3	-	-	-	-	-	-	-	-	-	-	-	-	-	-	-	-	-	-	3	3
PCR cycle	30	-	-	-	-	-	-	-	20	40	-	-	-	-	-	-	-	-	-	-	-	-	-	-	-	-
PCR efficiency	0.705	-	-	-	-	-	-	-	-	-	0.5	0.9	-	-	-	-	-	-	-	-	-	-	-	-	-	-
PCR error	1.00E-06	-	-	-	-	-	-	-	-	-	-	-	1.00E-05	1.00E-07	-	-	-	-	-	-	-	-	-	-	-	-
Barcode length	14	-	-	-	-	-	-	-	-	-	-	-	-	-	-	-	-	-	-	-	10	0-70	0-70	0-70	0-70	0-70
Reads per cell	50	-	-	-	-	-	-	-	-	-	-	-	-	-	25	100	1000	-	-	-	-	-	-	-	-	-
NGS profile	HS20	-	-	-	-	-	-	-	-	-	-	-	-	-	-	-	-	MSv1	-	-	-	-	-	-	-	-

Random barcode Hamming barcode VDJ barcode

Supplementary Figure 3. Read numbers for spurious sequences and true barcodes.

Boxplot represents the distribution of barcode read count in simulated sequencing data across various scenarios using CellBarcodeSim. The barcode read counts were log10-transformed. True barcodes are represented by blue boxes, while spurious sequences are shown in red. For each group, the data from 30 independent simulations were pooled together to create the box plots in which the median, interquartile range (IQR, the difference between the Q3 75th and Q2 25th percentiles of the data) are shown, and the outliers (beyond the whiskers of Q3 + 1.5IQR or Q2 - 1.5IQR) are plotted as dots. The table beneath the x-axis details the specific parameters used in each simulation scenario. Each row in the table corresponds to a unique parameter. The default simulation parameters are outlined in the first scenario located on the far left of the table (see Supplementary Table 2 for further details). Any variations from these default parameters in subsequent simulations are specifically noted and highlighted with a yellow background.

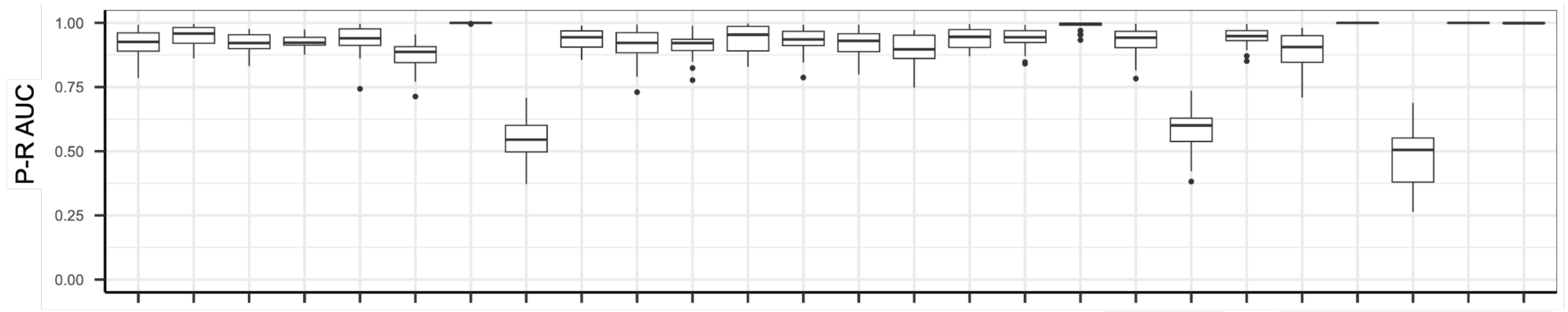

Barcode Type	Random	-	-	-	-	-	-	-	-	-	-	-	-	-	-	-	-	-	Hammin	Hammin	-	VDJ	VDJ	VDJ	VDJ	VDJ
Clone Number	300	150	600	1200	-	-	-	-	-	-	-	-	-	-	-	-	-	-	-	-	-	-	-	-	600	1200
Log clone size SD	2	-	-	-	-	-	1	3	-	-	-	-	-	-	-	-	-	-	-	3	-	-	1	3	1	1
Log clone size Mean	1.2	-	-	-	0.6	3	-	-	-	-	-	-	-	-	-	-	-	-	-	-	-	-	-	-	3	3
PCR cycle	30	-	-	-	-	-	-	-	20	40	-	-	-	-	-	-	-	-	-	-	-	-	-	-	-	-
PCR efficiency	0.705	-	-	-	-	-	-	-	-	-	0.5	0.9	-	-	-	-	-	-	-	-	-	-	-	-	-	-
PCR error	1.00E-06	-	-	-	-	-	-	-	-	-	-	-	1.00E-05	1.00E-07	-	-	-	-	-	-	-	-	-	-	-	-
Barcode length	14	-	-	-	-	-	-	-	-	-	-	-	-	-	-	-	-	-	-	-	10	0-70	0-70	0-70	0-70	0-70
Reads per cell	50	-	-	-	-	-	-	-	-	-	-	-	-	-	25	100	1000	-	-	-	-	-	-	-	-	-
NGS profile	HS20	-	-	-	-	-	-	-	-	-	-	-	-	-	-	-	-	MSv1	-	-	-	-	-	-	-	-

Random barcode Hamming barcode VDJ barcode

Supplementary Figure 4. AUC of precision-recall curve after reads count threshold filtering.

Boxplot shows the value of area under the curve (AUC) of precision-recall curve (P-R AUC) across various scenarios using CellBarcodeSim. The specific simulation scenario is indicated in the table below on the x-axis. The table beneath the x-axis details the specific parameters used in each simulation scenario. Each row in the table corresponds to a unique parameter. The default simulation parameters are outlined in the first scenario located on the far left of the table (also see Supplementary Table 2 for further details). Any variations from these default parameters in subsequent simulations are specifically noted and highlighted with a yellow background. For each scenario, there are 30 independent simulations. For each scenario, the data from 30 independent simulations were pooled together to create the box plots in which the median, interquartile range (IQR, the difference between the Q3 75th and Q2 25th percentiles of the data) are shown, and the outliers (beyond the whiskers of Q3 + 1.5IQR or Q2 - 1.5IQR) are plotted as dots.

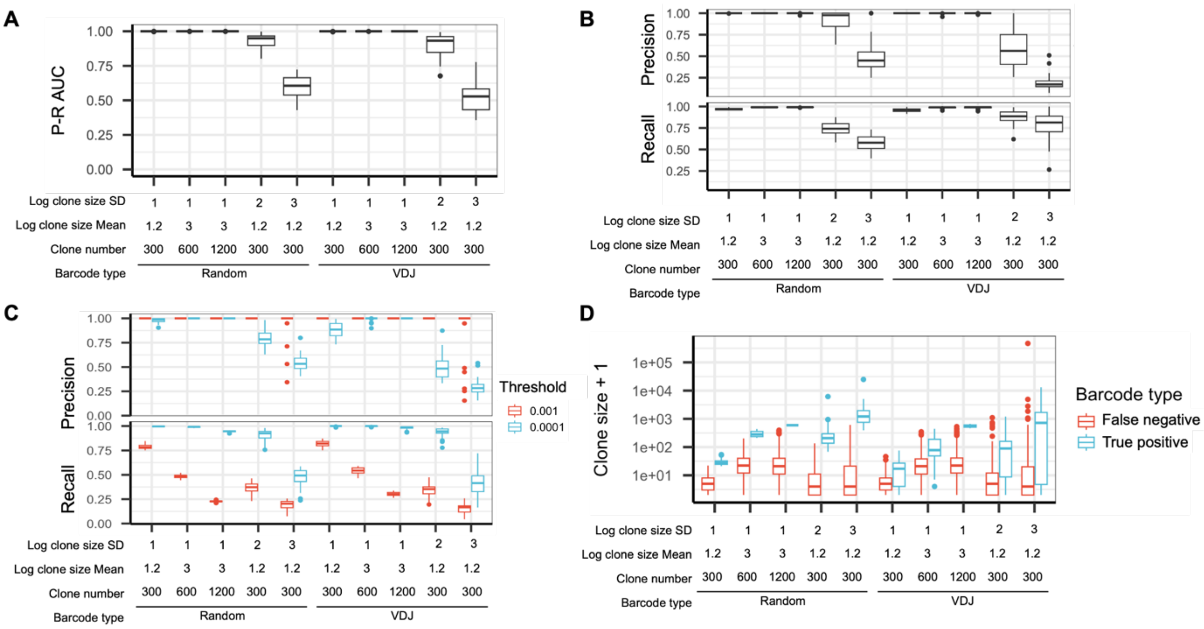

Supplementary Figure 5. Precision and recall for different values of threshold.

A. the value of area under the curve (AUC) of precision-recall curve (P-R AUC) and B. the precision and recall value for different thresholds, for simulated scenarios with different log clone size SD, clone size log-mean, clone numbers and barcode types. For precision and recall value in B, we used 4 thresholds: automatic, or 0.01, 0.001 and 0.0001 of total reads which are color-coded. C. the clone size of true positive barcodes and false negative barcodes when using a relative read count threshold of 0.01 for filtering. For each scenario, the data from 30 independent simulations were pooled together to create the box plots in which the median, interquartile range (IQR, the difference between the Q3 75th and Q2 25th percentiles of the data) are shown, and the outliers (beyond the whiskers of Q3 + 1.5IQR or Q2 - 1.5IQR) are plotted as dots.

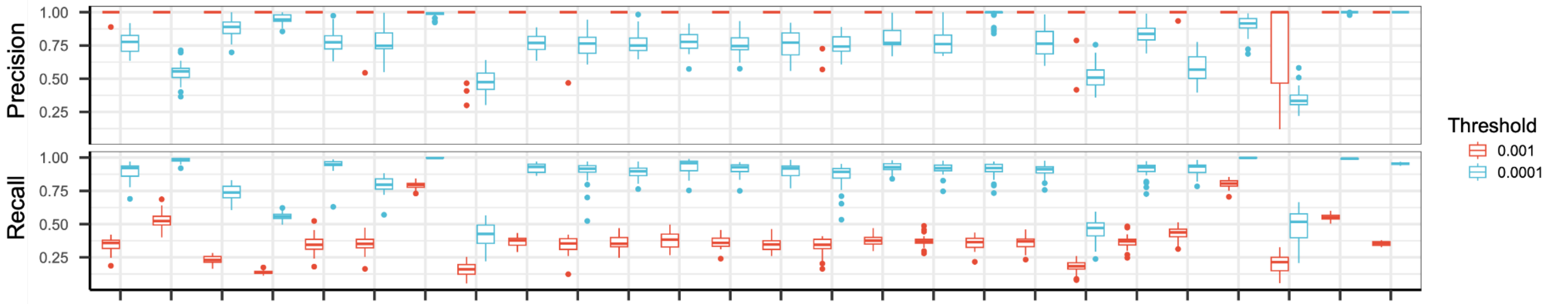

Barcode Type	Random	-	-	-	-	-	-	-	-	-	-	-	-	-	-	-	-	-	Hammin	Hammin	-	VDJ	VDJ	VDJ	VDJ	VDJ
Clone Number	300	150	600	1200	-	-	-	-	-	-	-	-	-	-	-	-	-	-	-	-	-	-	-	-	600	1200
Log clone size SD	2	-	-	-	-	-	1	3	-	-	-	-	-	-	-	-	-	-	-	3	-	-	1	3	1	1
Log clone size Mean	1.2	-	-	-	0.6	3	-	-	-	-	-	-	-	-	-	-	-	-	-	-	-	-	-	-	3	3
PCR cycle	30	-	-	-	-	-	-	-	20	40	-	-	-	-	-	-	-	-	-	-	-	-	-	-	-	-
PCR efficiency	0.705	-	-	-	-	-	-	-	-	-	0.5	0.9	-	-	-	-	-	-	-	-	-	-	-	-	-	-
PCR error	1.00E-06	-	-	-	-	-	-	-	-	-	-	-	1.00E-05	1.00E-07	-	-	-	-	-	-	-	-	-	-	-	-
Barcode length	14	-	-	-	-	-	-	-	-	-	-	-	-	-	-	-	-	-	-	-	10	0-70	0-70	0-70	0-70	0-70
Reads per cell	50	-	-	-	-	-	-	-	-	-	-	-	-	-	25	100	1000	-	-	-	-	-	-	-	-	-
NGS profile	HS20	-	-	-	-	-	-	-	-	-	-	-	-	-	-	-	-	MSv1	-	-	-	-	-	-	-	-

Random barcode   Hamming barcode   VDJ barcode

Supplementary Figure 6. Precision and recall for different values of threshold.

Boxplot shows precision and recall of different thresholds for various simulated scenarios. The threshold used is color-coded with the automatic threshold based on k-means clustering in red and read proportion thresholds of 0.01, 0.001, and 0.0001 in different types of blue. The table beneath the x-axis details the specific parameters used in each simulation scenario. Each row in the table corresponds to a unique parameter. The default simulation parameters are outlined in the first scenario located on the far left of the table (also see Supplementary Table 2 for further details). Any variations from these default parameters in subsequent simulations are specifically noted and highlighted with a yellow background. For each group, the data from 30 independent simulations were pooled together to create the box plots in which the median, interquartile range (IQR, the difference between the Q3 75th and Q2 25th percentiles of the data) are shown, and the outliers (beyond the whiskers of Q3 + 1.5IQR or Q2 - 1.5IQR) are plotted as dots.

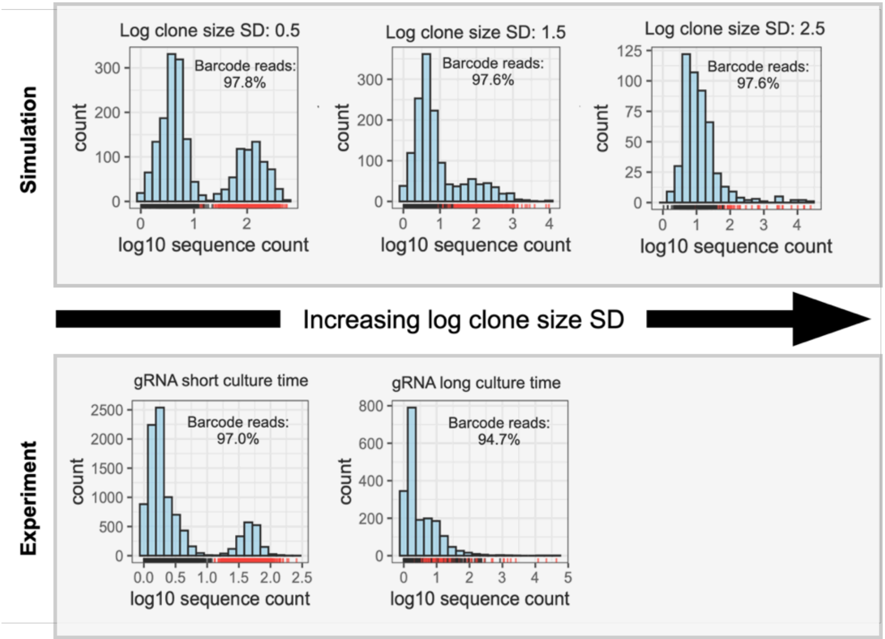

Supplementary Figure 7. Read frequency distributions for CRISPR gRNA experiments

The simulations (top row) depict a series of increasing log clone size SD (0.5, 1.5, 2.5). Experimental CRISPR gRNA sequencing is shown (bottom row) with short or long culture representing small and big clone size-SD respectively. For comparability, the histograms represent sequence read frequency distribution of 10^5 sampled reads. The rug plot beneath the histogram visualizes each sequence, with random jitter between [0,1] added to aid visualization of individual sequences. Barcodes in the reference library are colored in red while others are shown in black. The percentage of barcode-containing reads is indicated in the top right corner.

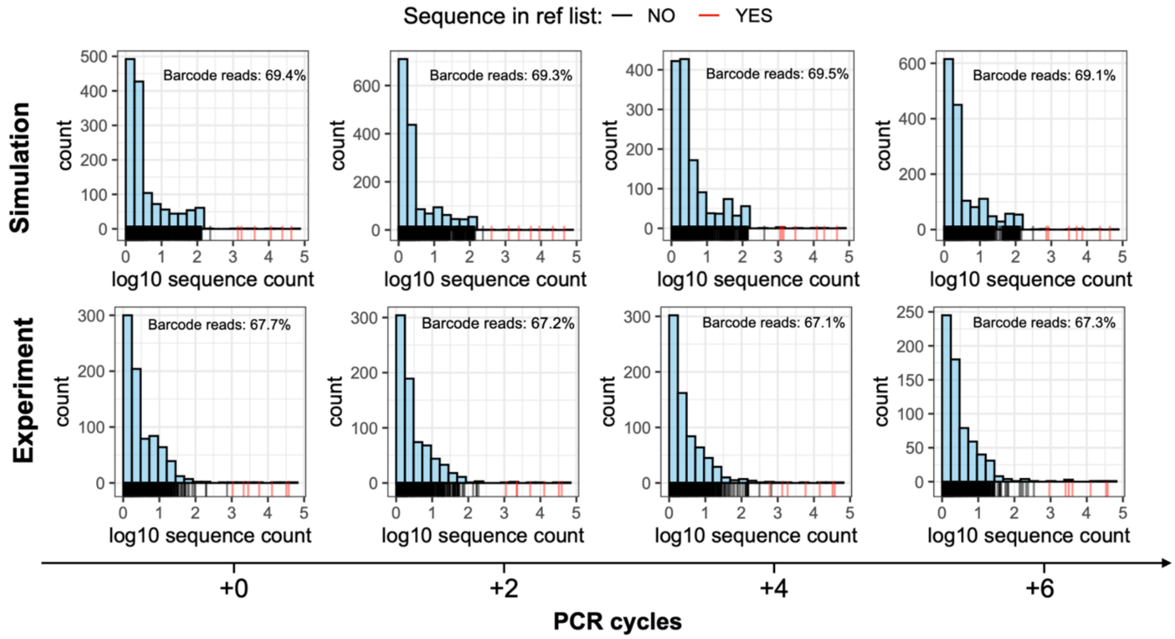

Supplementary Figure 8. Sequencing of VDJ-barcoded MEF data with different PCR cycle numbers.

A. Histograms of sequence frequencies, with lines below indicating whether each sequence is in the reference (red) or not (black). The simulated (first row) and experimental (second row) barcode sequencing results are organized based on a gradient of PCR cycles, starting from the reference cycle and then increasing by 2, 4, or 6 cycles. The percentage displayed at the top of each panel represents the percentage of total reads containing specific sequence patterns.

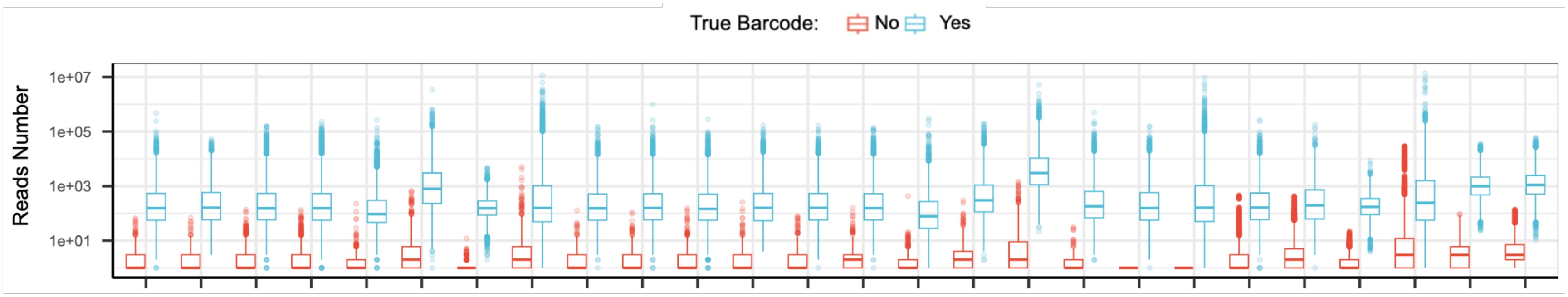

Barcode Type	Random	-	-	-	-	-	-	-	-	-	-	-	-	-	-	-	-	Hammin	Hammin	-	VDJ	VDJ	VDJ	VDJ	VDJ
Clone Number	300	150	600	1200	-	-	-	-	-	-	-	-	-	-	-	-	-	-	-	-	-	-	-	600	1200
Log clone size SD	2	-	-	-	-	-	1	3	-	-	-	-	-	-	-	-	-	-	3	-	-	1	3	1	1
Log clone size Mean	1.2	-	-	-	0.6	3	-	-	-	-	-	-	-	-	-	-	-	-	-	-	-	-	-	3	3
PCR cycle	30	-	-	-	-	-	-	-	20	40	-	-	-	-	-	-	-	-	-	-	-	-	-	-	-
PCR efficiency	0.705	-	-	-	-	-	-	-	-	-	0.5	0.9	-	-	-	-	-	-	-	-	-	-	-	-	-
PCR error	1.00E-06	-	-	-	-	-	-	-	-	-	-	-	1.00E-05	1.00E-07	-	-	-	-	-	-	-	-	-	-	-
Barcode length	14	-	-	-	-	-	-	-	-	-	-	-	-	-	-	-	-	-	-	10	0-70	0-70	0-70	0-70	0-70
Reads per cell	50	-	-	-	-	-	-	-	-	-	-	-	-	-	25	100	1000	-	-	-	-	-	-	-	-
NGS profile	HS20	-	-	-	-	-	-	-	-	-	-	-	-	-	-	-	-	MSv1	-	-	-	-	-	-	-

Random barcode Hamming barcode VDJ barcode

Supplementary Figure 9. Reads number of spurious sequences and true barcodes after reference library filtering.

Boxplot depicts the distribution of barcode read counts in simulated sequencing data across various scenarios using CellBarcodeSim after reference library filtering. The barcode read counts were log10-transformed. True barcodes are represented by blue boxes, while spurious sequences are shown in red. For each scenario, the data from 30 independent simulations were pooled together to create the box plots in which the median, interquartile range (IQR, the difference between the Q3 75th and Q2 25th percentiles of the data) are shown, and the outliers (beyond the whiskers of Q3 + 1.5IQR or Q2 - 1.5IQR) are plotted as dots. The table beneath the x-axis details the specific parameters used in each simulation scenario. Each row in the table corresponds to a unique parameter. The default simulation parameters are outlined in the first scenario located on the far left of the table (see Supplementary Table 2 for further details). Any variations from these default parameters in subsequent simulations are specifically noted and highlighted with a yellow background.

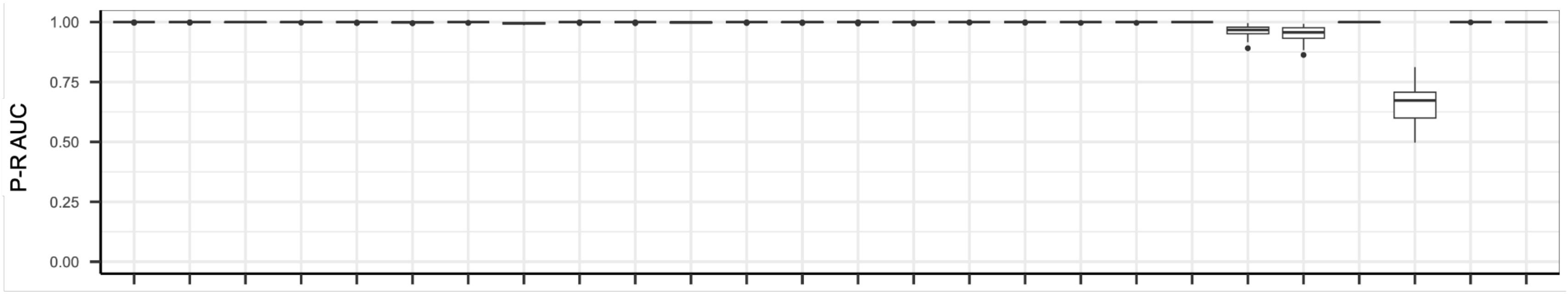

Barcode Type	Random	-	-	-	-	-	-	-	-	-	-	-	-	-	-	-	-	-	Hammin	Hammin	-	VDJ	VDJ	VDJ	VDJ	VDJ
Clone Number	300	150	600	1200	-	-	-	-	-	-	-	-	-	-	-	-	-	-	-	-	-	-	-	-	600	1200
Log clone size SD	2	-	-	-	-	-	1	3	-	-	-	-	-	-	-	-	-	-	-	3	-	-	1	3	1	1
Log clone size Mean	1.2	-	-	-	0.6	3	-	-	-	-	-	-	-	-	-	-	-	-	-	-	-	-	-	-	3	3
PCR cycle	30	-	-	-	-	-	-	-	20	40	-	-	-	-	-	-	-	-	-	-	-	-	-	-	-	-
PCR efficiency	0.705	-	-	-	-	-	-	-	-	-	0.5	0.9	-	-	-	-	-	-	-	-	-	-	-	-	-	-
PCR error	1.00E-06	-	-	-	-	-	-	-	-	-	-	-	1.00E-05	1.00E-07	-	-	-	-	-	-	-	-	-	-	-	-
Barcode length	14	-	-	-	-	-	-	-	-	-	-	-	-	-	-	-	-	-	-	-	10	0-70	0-70	0-70	0-70	0-70
Reads per cell	50	-	-	-	-	-	-	-	-	-	-	-	-	-	25	100	1000	-	-	-	-	-	-	-	-	-
NGS profile	HS20	-	-	-	-	-	-	-	-	-	-	-	-	-	-	-	-	MSv1	-	-	-	-	-	-	-	-

Random barcode Hamming barcode VDJ barcode

Supplementary Figure 10. AUC of precision-recall curve after reference library filtering.

Boxplot shows the value of area under the curve (AUC) of precision-recall curve (P-R AUC) across various scenarios using CellBarcodeSim after reference library filtering. The specific simulation scenario is indicated in the table below on the x-axis. The table beneath the x-axis details the specific parameters used in each simulation scenario. Each row in the table corresponds to a unique parameter. The default simulation parameters are outlined in the first scenario located on the far left of the table (also see Supplementary Table 2 for further details). Any variations from these default parameters in subsequent simulations are specifically noted and highlighted with a yellow background. For each scenario, the data from 30 independent simulations were pooled together to create the box plots in which the median, interquartile range (IQR, the difference between the Q3 75th and Q2 25th percentiles of the data) are shown, and the outliers (beyond the whiskers of Q3 + 1.5IQR or Q2 - 1.5IQR) are plotted as dots.

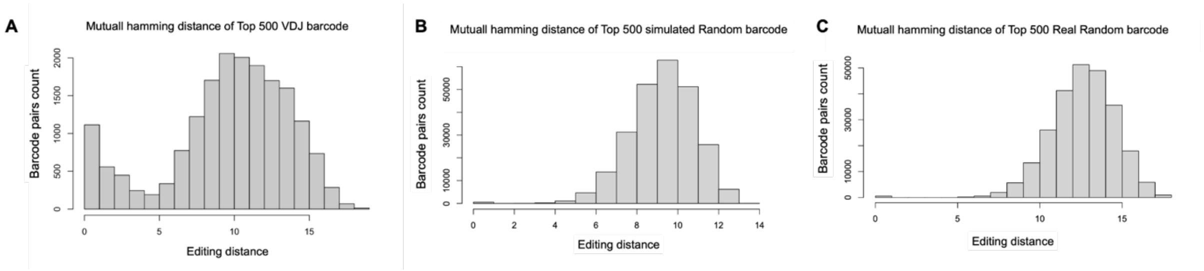

Supplementary Figure 11. Edit distance distribution for simulated VDJ and random barcodes.

The pairwise Hamming distance distribution histogram for A. simulated VDJ barcode. B. simulated 14bp fixed length random barcodes, and C. barcode library sequencing of 20bp fixed length random barcodes using the most frequent 500 barcodes for each type of barcode.

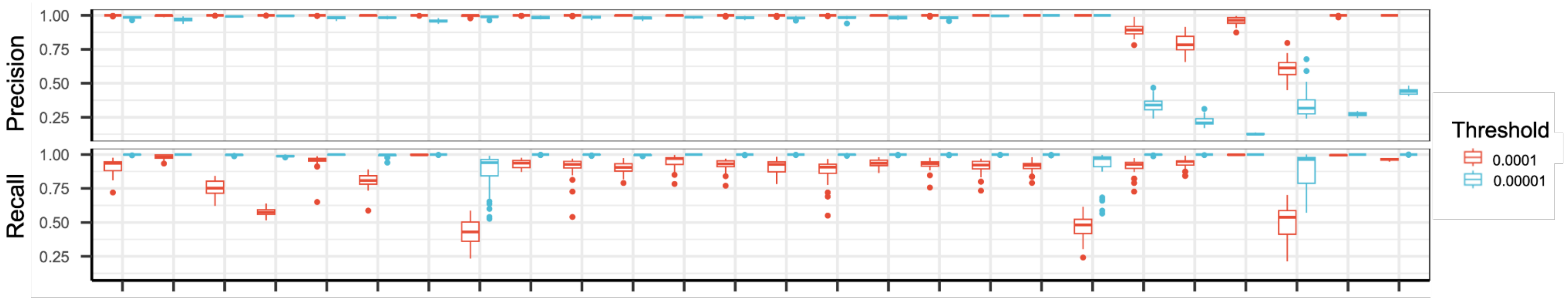

Barcode Type	Random	-	-	-	-	-	-	-	-	-	-	-	-	-	-	-	-	Hammin	Hammin	-	VDJ	VDJ	VDJ	VDJ	VDJ
Clone Number	300	150	600	1200	-	-	-	-	-	-	-	-	-	-	-	-	-	-	-	-	-	-	-	600	1200
Log clone size SD	2	-	-	-	-	-	1	3	-	-	-	-	-	-	-	-	-	-	3	-	-	1	3	1	1
Log clone size Mean	1.2	-	-	-	0.6	3	-	-	-	-	-	-	-	-	-	-	-	-	-	-	-	-	-	3	3
PCR cycle	30	-	-	-	-	-	-	-	20	40	-	-	-	-	-	-	-	-	-	-	-	-	-	-	-
PCR efficiency	0.705	-	-	-	-	-	-	-	-	-	0.5	0.9	-	-	-	-	-	-	-	-	-	-	-	-	-
PCR error	1.00E-06	-	-	-	-	-	-	-	-	-	-	-	1.00E-05	1.00E-07	-	-	-	-	-	-	-	-	-	-	-
Barcode length	14	-	-	-	-	-	-	-	-	-	-	-	-	-	-	-	-	-	-	10	0-70	0-70	0-70	0-70	0-70
Reads per cell	50	-	-	-	-	-	-	-	-	-	-	-	-	-	25	100	1000	-	-	-	-	-	-	-	-
NGS profile	HS20	-	-	-	-	-	-	-	-	-	-	-	-	-	-	-	-	MSv1	-	-	-	-	-	-	-

Random barcode   Hamming barcode   VDJ barcode

Supplementary Figure 12. Precision and recall for different threshold values after reference library filtering.

Boxplot shows precision and recall of different thresholds for various simulated scenarios after reference library filtering. The threshold used is color-coded with the automatic threshold based on k-means clustering in red and read proportion thresholds of 0.01, 0.001, and 0.0001 in different types of blue. The table beneath the x-axis details the specific parameters used in each simulation scenario. Each row in the table corresponds to a unique parameter. The default simulation parameters are outlined in the first scenario located on the far left of the table (also see Supplementary Table 2 for further details). Any variations from these default parameters in subsequent simulations are specifically noted and highlighted with a yellow background. For each scenario, the data from 30 independent simulations were pooled together to create the box plots in which the median, interquartile range (IQR, the difference between the Q3 75th and Q2 25th percentiles of the data) are shown, and the outliers (beyond the whiskers of Q3 + 1.5IQR or Q2 - 1.5IQR) are plotted as dots.

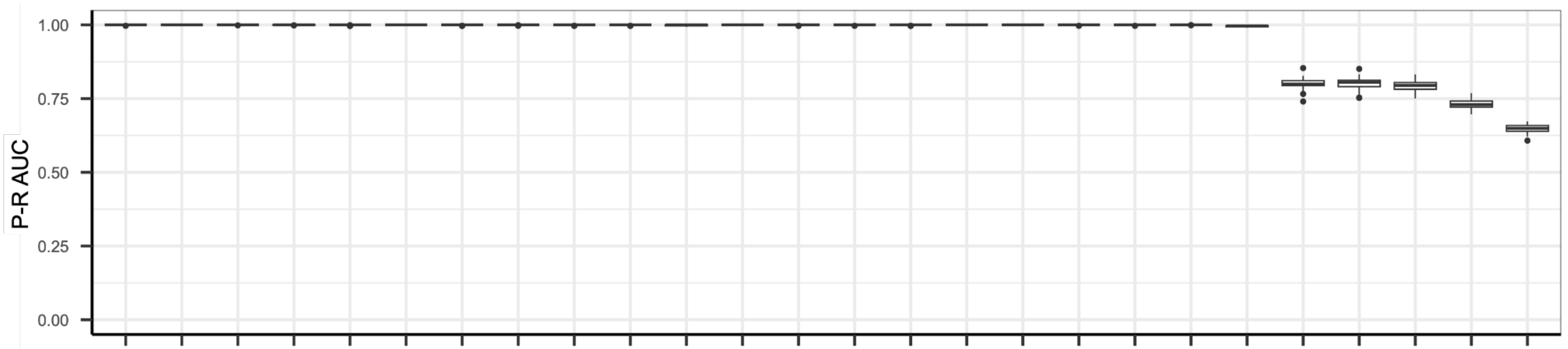

Barcode Type	Random	-	-	-	-	-	-	-	-	-	-	-	-	-	-	-	-	-	Hammin	Hammin	-	VDJ	VDJ	VDJ	VDJ	VDJ
Clone Number	300	150	600	1200	-	-	-	-	-	-	-	-	-	-	-	-	-	-	-	-	-	-	-	-	600	1200
Log clone size SD	2	-	-	-	-	-	1	3	-	-	-	-	-	-	-	-	-	-	-	3	-	-	1	3	1	1
Log clone size Mean	1.2	-	-	-	0.6	3	-	-	-	-	-	-	-	-	-	-	-	-	-	-	-	-	-	-	3	3
PCR cycle	30	-	-	-	-	-	-	-	20	40	-	-	-	-	-	-	-	-	-	-	-	-	-	-	-	-
PCR efficiency	0.705	-	-	-	-	-	-	-	-	-	0.5	0.9	-	-	-	-	-	-	-	-	-	-	-	-	-	-
PCR error	1.00E-06	-	-	-	-	-	-	-	-	-	-	-	1.00E-05	1.00E-07	-	-	-	-	-	-	-	-	-	-	-	-
Barcode length	14	-	-	-	-	-	-	-	-	-	-	-	-	-	-	-	-	-	-	-	10	0-70	0-70	0-70	0-70	0-70
Reads per cell	50	-	-	-	-	-	-	-	-	-	-	-	-	-	25	100	1000	-	-	-	-	-	-	-	-	-
NGS profile	HS20	-	-	-	-	-	-	-	-	-	-	-	-	-	-	-	-	MSv1	-	-	-	-	-	-	-	-

Random barcode Hamming barcode VDJ barcode

Supplementary Figure 13. AUC of precision-recall curve after cluster filtering.

Boxplot shows the value of area under the curve (AUC) of precision-recall curve (P-R AUC) across various scenarios using CellBarcodeSim after cluster filtering. Barcodes that had an edit distance below 2 from a more abundant barcode were removed. The specific simulation scenario is indicated in the table below on the x-axis. The table beneath the x-axis details the specific parameters used in each simulation scenario. Each row in the table corresponds to a unique parameter. The default simulation parameters are outlined in the first scenario located on the far left of the table (also see Supplementary Table 2 for further details). Any variations from these default parameters in subsequent simulations are specifically noted and highlighted with a yellow background. For each scenario, the data from 30 independent simulations were pooled together to create the box plots in which the median, interquartile range (IQR, the difference between the Q3 75th and Q2 25th percentiles of the data) are shown, and the outliers (beyond the whiskers of Q3 + 1.5IQR or Q2 - 1.5IQR) are plotted as dots.

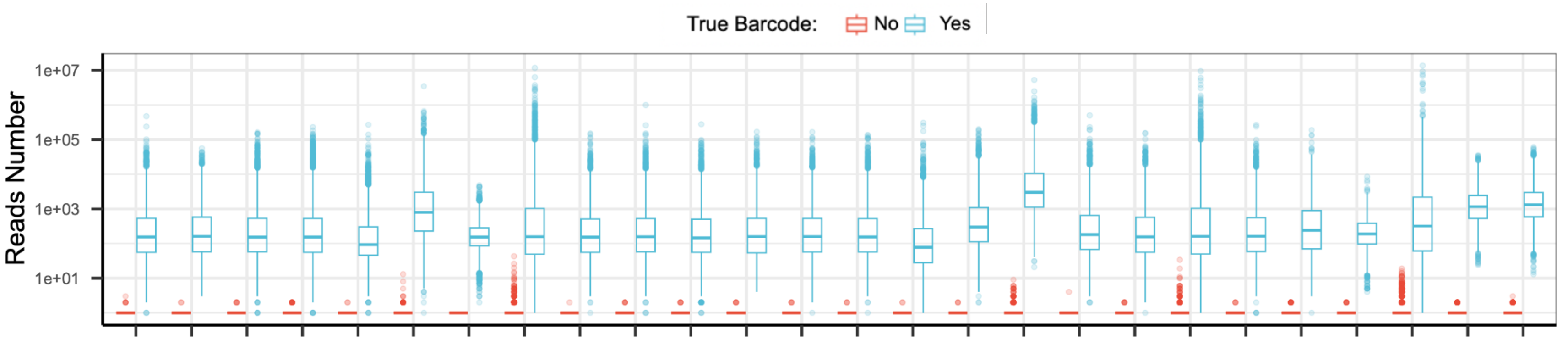

Barcode Type	Random	-	-	-	-	-	-	-	-	-	-	-	-	-	-	-	-	-	Hammin	Hammin	-	VDJ	VDJ	VDJ	VDJ	VDJ
Clone Number	300	150	600	1200	-	-	-	-	-	-	-	-	-	-	-	-	-	-	-	-	-	-	-	-	600	1200
Log clone size SD	2	-	-	-	-	-	1	3	-	-	-	-	-	-	-	-	-	-	-	3	-	-	1	3	1	1
Log clone size Mean	1.2	-	-	-	0.6	3	-	-	-	-	-	-	-	-	-	-	-	-	-	-	-	-	-	-	3	3
PCR cycle	30	-	-	-	-	-	-	-	20	40	-	-	-	-	-	-	-	-	-	-	-	-	-	-	-	-
PCR efficiency	0.705	-	-	-	-	-	-	-	-	-	0.5	0.9	-	-	-	-	-	-	-	-	-	-	-	-	-	-
PCR error	1.00E-06	-	-	-	-	-	-	-	-	-	-	-	1.00E-05	1.00E-07	-	-	-	-	-	-	-	-	-	-	-	-
Barcode length	14	-	-	-	-	-	-	-	-	-	-	-	-	-	-	-	-	-	-	-	10	0-70	0-70	0-70	0-70	0-70
Reads per cell	50	-	-	-	-	-	-	-	-	-	-	-	-	-	25	100	1000	-	-	-	-	-	-	-	-	-
NGS profile	HS20	-	-	-	-	-	-	-	-	-	-	-	-	-	-	-	-	MSv1	-	-	-	-	-	-	-	-

Random barcode | Hamming barcode | VDJ barcode

Supplementary Figure 14. Read numbers of spurious sequences and true barcodes after cluster filtering.

Boxplot represents the distribution of barcode read counts in simulated sequencing data across various scenarios using CellBarcodeSim after cluster filtering. Barcodes that had an edit distance below 2 from a more abundant barcode were removed. The barcode read counts were log10-transformed. True barcodes are represented by blue boxes, while spurious sequences are shown in red. For each scenario, the data from 30 independent simulations were pooled together to create the box plots in which the median, interquartile range (IQR, the difference between the Q3 75th and Q2 25th percentiles of the data) are shown, and the outliers (beyond the whiskers of Q3 + 1.5IQR or Q2 - 1.5IQR) are plotted as dots. The table beneath the x-axis details the specific parameters used in each simulation scenario. Each row in the table corresponds to a unique parameter. The default simulation parameters are outlined in the first scenario located on the far left of the table (also see Supplementary Table 2 for further details). Any variations from these default parameters in subsequent simulations are specifically noted and highlighted with a yellow background.

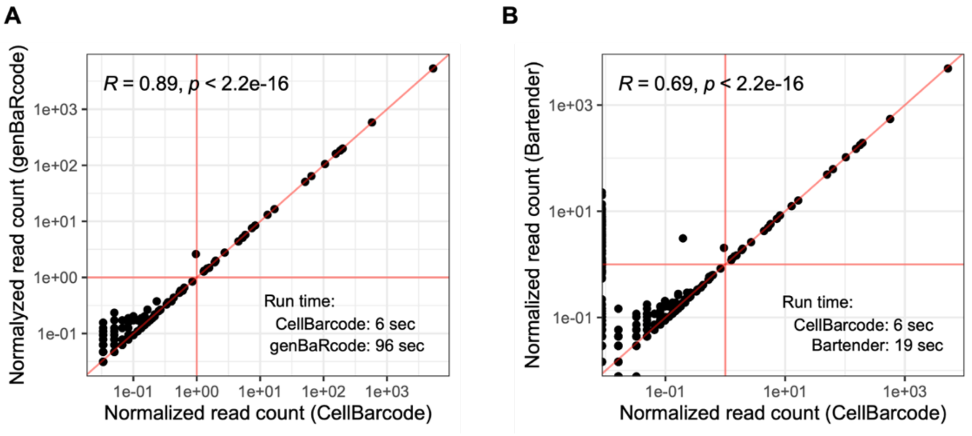

Supplementary Figure 15. Comparing CellBarcode with genBaRcode and Bartender using lentiviral barcoding data.

A, B. CellBarcode is compared to genBaRcode (A) and Bartender (B) on data from (Eisele et al.). Each dot represents a barcode. The read counts were normalized to cell counts, with the horizontal and vertical lines representing the 1 cell threshold. The spearman correlation and p value (two sided) are displayed in the top left corner, while the red diagonal line represents y=x. The runtime is displayed in the bottom right corner of each figure.

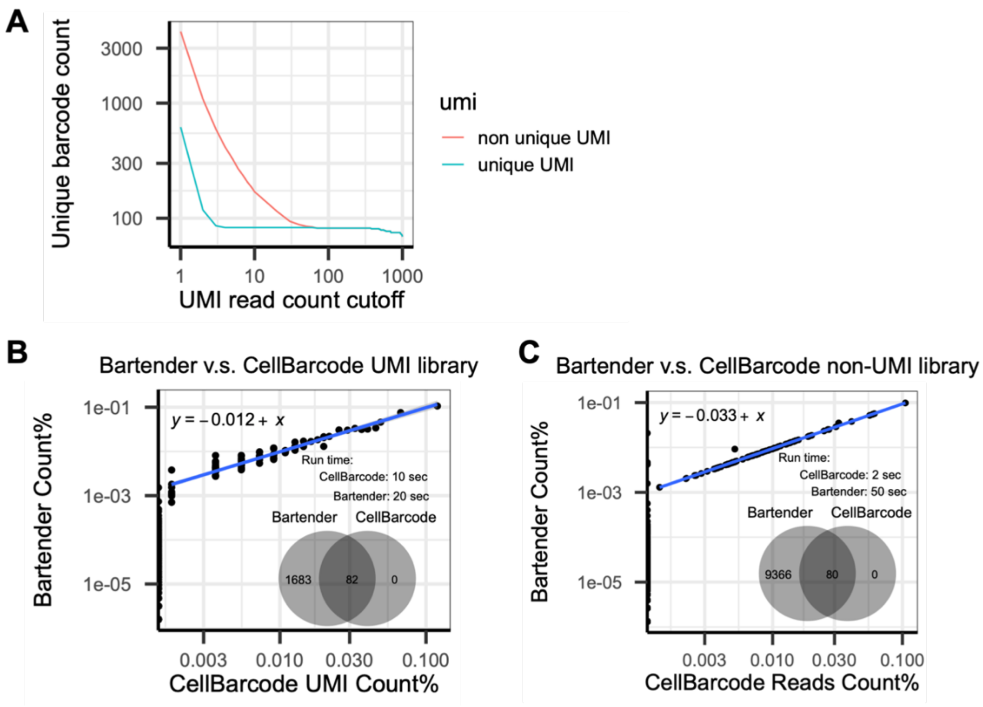

Supplementary Figure 16. UMI read count threshold optimization and comparing CellBarcode with Bartender using the in vivo VDJ barcode sequencing.

A. The threshold for UMI-reads was tested within a range of 1 to 1000 on the x-axis, while the corresponding count of retained unique barcode numbers was plotted on the y-axis, differentiated by whether UMIs were assumed non-unique (default, green) or unique (red). In the latter case, for each UMI, only the barcode with the highest abundance was retained.
B and C. CellBarcode is compared to Bartender using either UMI library sequencing (B) or non-UMI library (C) of VDJ barcodes. Each dot represents a barcode, with the UMI count or read counts normalized to read total counts. The fitted linear model (in blue) and p value are displayed in the top left corner, along with the blue line fitted by linear regression. The venn plot shows the shared barcode count, and above is the run time for each analysis.

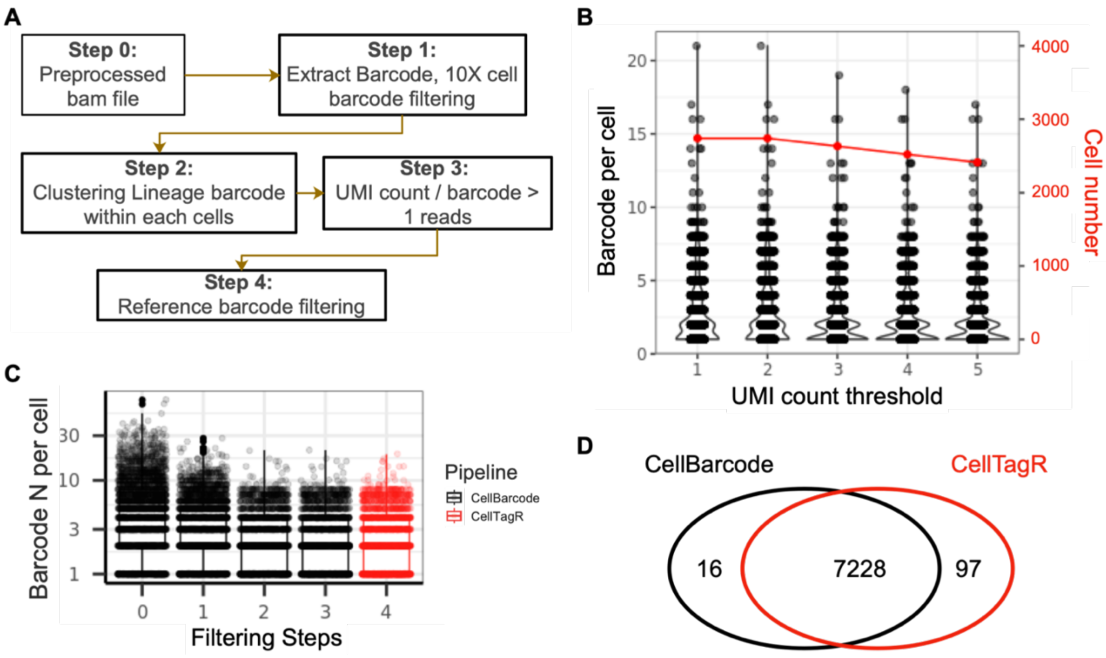

Supplementary Figure 17. Processing CellTag barcoded scRNA-seq data using CellBarcode.

A. The pipeline used to analyze CellTag data with CellBarcode. Data from Biddy et al (2018).
B. The number of lineage barcodes per cell (black) and the number of cells with a unique barcode (red) for different UMI-count-per-barcode thresholds. The data was first filtered until step 2 of A . Each dot is a cell.
C. Number of barcodes per cell after each CellBarcode filtering step (black) and the CellTagR pipeline results (red). The median and interquartile range (IQR, the difference between the 75th and 25th percentiles of the data) are shown in the boxplot with n>2000 cells for all the filtering steps, and the outliers (beyond the whiskers of Q3 + 1.5IQR or Q2 - 1.5IQR) are plotted as dots.
D. Comparison of barcodes processed with the CellBarcode and CellTagR pipelines.

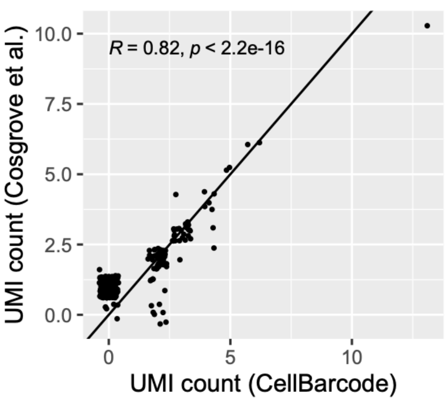

Supplementary Figure 18. Comparing scRNA-seq barcode extraction using CellBarcode with the original results in Cosgrove et al.

The UMI count for each barcode from CellBarcode versus the one from Cosgrove et al (2023). Spearman correlation and p value (two sided) is in the top left of the figure. For Cosgrove et al. (2023)'s methodology, only UMIs with three or more reads and a primary VDJ barcode (classified as having >=0.45 reads) were included. They then retrieved the leading barcode for each UMI. A VDJ barcode was linked to a 10x cell if there was a high consensus across UMIs, specified as >=0.75 agreement among the remaining UMIs. If only one UMI remained, it took an additional step to verify that the VDJ barcode for this UMI was the leading barcode across all reads for that cell, possessing >=0.45 of the reads. For the CellBarcode procedure, we first applied the leading barcode per UMI filter and set the UMI read threshold to >= 2. In the figure, each dot signifies a cell, with the distribution portrayed by the violin plot. The red line plot shows the number of uniquely barcoded cells left after applying varying UMI count filters, as described on the x-axis.

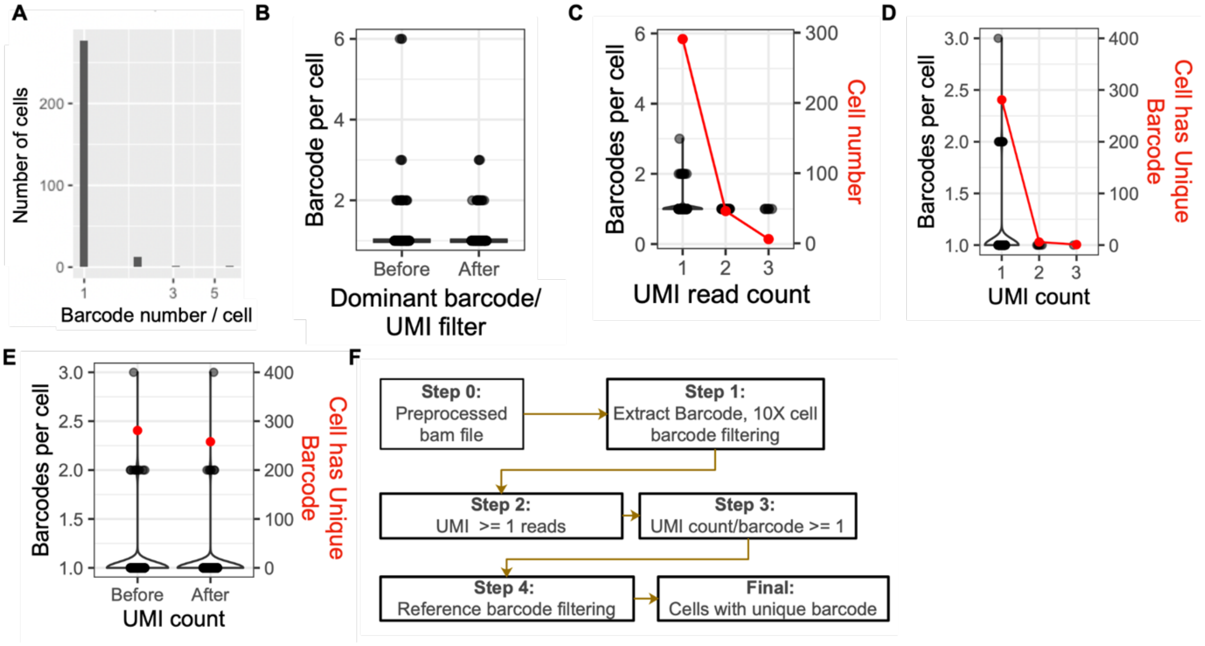

Figure Supplementary 19. Analysis of Marsolier et al 2022 barcoded scRNA-seq data using CellBarcode.

A. Number of cells with a given number of lineage barcodes in the Marsolier et al. (2022) dataset.
B. The number of lineage barcodes per cell before and after filtering based on the dominant barcode per UMI in the Marsolier et al. (2022) dataset. In the scatter plot the y-axis is the barcode number in a cell, each dot represents a cell, and the distribution is shown by the violin plot.
C. The number of lineage barcodes per cell (corresponding to the left y-axis in black) and the cell number (corresponding to the right y-axis in red) for different thresholds of read per UMI. The data was first processed with the dominant barcode per UMI filter. Each black dot represents a cell while the violin plot shows the distribution of the barcode number per cell.
D. The number of lineage barcodes per cell (corresponding to the left black y-axis in black) and the number of cells with a unique barcode (corresponding to the right y-axis in red) for different thresholds of UMI count per barcode. The data was first processed with the dominant barcode per UMI filter and the UMI read threshold >= 1. In the figure, each dot represents a cell, and the distribution is shown by the violin plot. The red line plot represents the numbwer of remaining unique barcoded cells after applying different UMI count filtering described on the x-axis.
E. The number of lineage barcodes found per cell (black) and the number of cells with a unique barcode (red) before and after reference library filtering provided in Marsolier et al. (2022) Each dot is a cell, the violin plot shows the distribution. The data was first processed with the dominant barcode per UMI filter, the UMI read threshold >=1 and the UMI count per barcode >=1.
F. Scheme of the CellBarcode analysis pipeline and corresponding thresholds in each step.

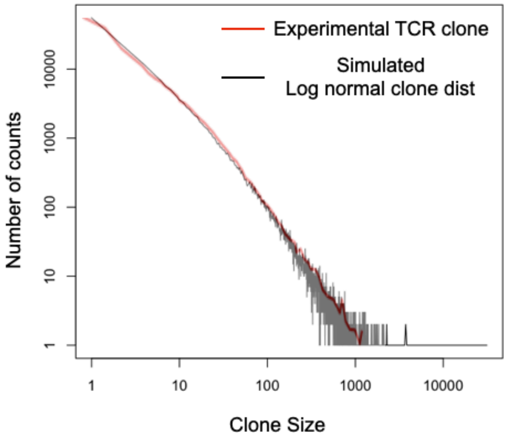

Figure Supplementary 20. Clone size distribution simulation.

Number of clones for different clone size frequency (ordered) for murine T cell beta chain sequencing data (plotted as presented in original paper of Desponds et al. 2016) and simulated barcode data with log-normal distribution with log mean of 1.2 and log SD of 2.

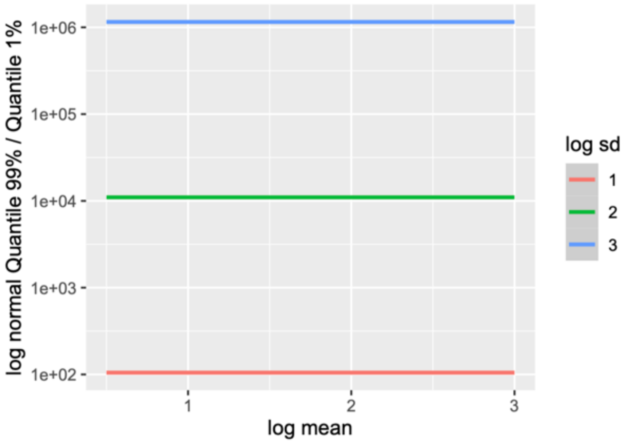

Supplementary Figure 21. Log clone size SD and clone size range.

Line plot depicting the ratio between the 99th quantile, Q(0.99), and the 1st quantile, Q(0.01), of the log-normally distributed clone size. The plot illustrates that, for a given log clone size SD, the ratio of Q(0.99)/Q(0.01) is constant across log-mean values, with the ratio depicted for the three log clone size SDs used in the simulation study. This is due to the mathematical form of the log-normal distribution, as described in the Methods.

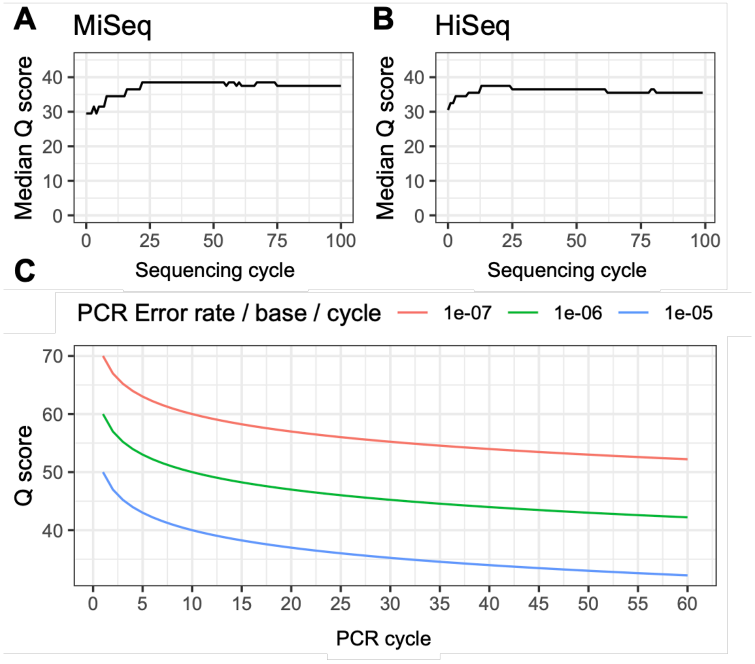

Supplementary Figure 22. Profiling NGS Sequencing error and PCR error.

A. MiSeq and B. HiSeq Sequencing quality score profiling from ART sequencing simulator. The y-axis shows the median sequencing quality in each sequencing cycle (x-axis).
C. The cumulative PCR error (y-axis) for different cycles (x-axis) and the error rate per base per cycle. The error rate per base per cycle is noted by colors with 1e-7, 1e-6 and 1e-5 representing the fidelity of Q5, Phusion and Taq PCR enzymes.
The quality score (Q score) is defined by Q = -10log10(error).

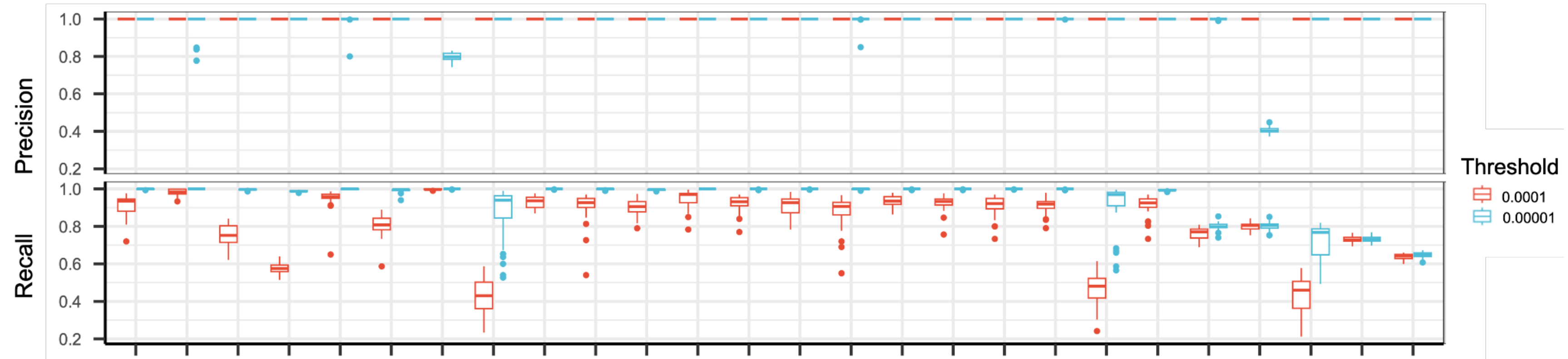

Barcode Type	Random	-	-	-	-	-	-	-	-	-	-	-	-	-	-	-	-	-	Hammin	Hammin	-	VDJ	VDJ	VDJ	VDJ	VDJ
Clone Number	300	150	600	1200	-	-	-	-	-	-	-	-	-	-	-	-	-	-	-	-	-	-	-	-	600	1200
Log clone size SD	2	-	-	-	-	-	1	3	-	-	-	-	-	-	-	-	-	-	-	3	-	-	1	3	1	1
Log clone size Mean	1.2	-	-	-	0.6	3	-	-	-	-	-	-	-	-	-	-	-	-	-	-	-	-	-	-	3	3
PCR cycle	30	-	-	-	-	-	-	-	20	40	-	-	-	-	-	-	-	-	-	-	-	-	-	-	-	-
PCR efficiency	0.705	-	-	-	-	-	-	-	-	-	0.5	0.9	-	-	-	-	-	-	-	-	-	-	-	-	-	-
PCR error	1.00E-06	-	-	-	-	-	-	-	-	-	-	-	1.00E-05	1.00E-07	-	-	-	-	-	-	-	-	-	-	-	-
Barcode length	14	-	-	-	-	-	-	-	-	-	-	-	-	-	-	-	-	-	-	-	10	0-70	0-70	0-70	0-70	0-70
Reads per cell	50	-	-	-	-	-	-	-	-	-	-	-	-	-	25	100	1000	-	-	-	-	-	-	-	-	-
NGS profile	HS20	-	-	-	-	-	-	-	-	-	-	-	-	-	-	-	-	MSv1	-	-	-	-	-	-	-	-

Random barcode Hamming barcode VDJ barcode

Supplementary Figure 23. Precision and recall for different threshold values after cluster filtering.

Boxplot shows precision and recall of different thresholds for various simulated scenarios after cluster filtering. Barcodes that had an edit distance below 2 from a more abundant barcode were removed, then a threshold based on read count was applied to remove spurious barcodes. The threshold used is color-coded with the automatic threshold based on k-means clustering in red and read proportion thresholds of 0.01, 0.001, and 0.0001 in different types of blue. The table beneath the x-axis details the specific parameters used in each simulation scenario. Each row in the table corresponds to a unique parameter. The default simulation parameters are outlined in the first scenario located on the far left of the table (also see Supplementary Table 2 for further details). Any variations from these default parameters in subsequent simulations are specifically noted and highlighted with a yellow background. For each scenario, the data from 30 independent simulations were pooled together to create the box plots in which the median, interquartile range (IQR, the difference between the Q3 75th and Q2 25th percentiles of the data) are shown, and the outliers (beyond the whiskers of Q3 + 1.5IQR or Q2 - 1.5IQR) are plotted as dots.

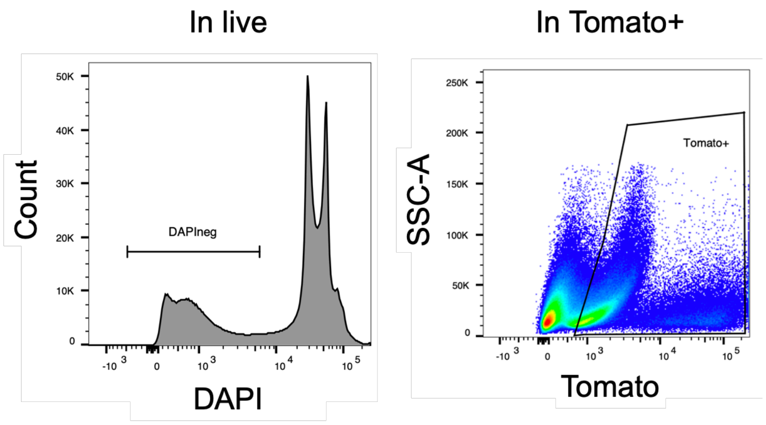

Supplementary 24. Cell sorting strategy used to obtain gRNA infected cells.

Live cells were selected using a DAPI staining. Subsequently, we enriched the population with gRNA-expression cells by sorting for Tomato-positive cells, which constituted 31% of the total live cells.

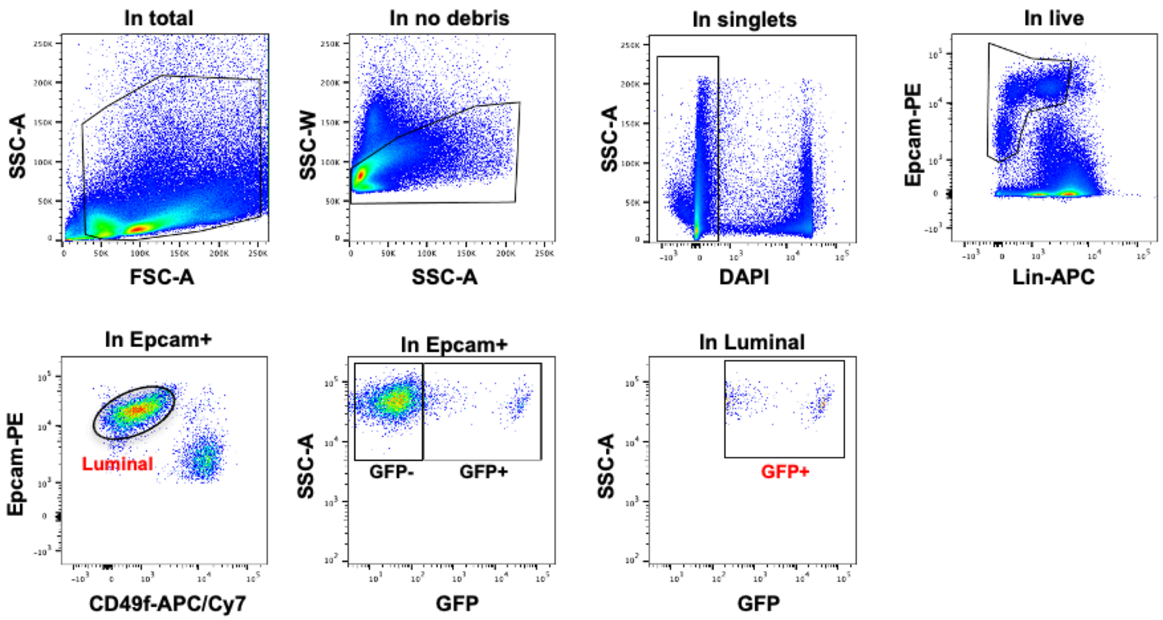

Supplementary 25. Cell sorting strategy used to obtain Luminal barcoded cells.

We selected GFP-positive cells within the luminal population gate, which accounted for approximately 6% of the total luminal cells, for analysis. These cells expressed the barcode and exhibited a purity level of about 95% post-sorting.

Supplementary vignette 1

```r
library(data.table)
library(ggplot2)
library(CellBarcode)
library(plyr)
```

Introduction

This document aims to demonstrate bulk cellular barcode sequencing analysis.

There are two part, the part 1 is focusing of using the CellBarcode to analysis bulk cellular barcode data.

The part 2 is about simulating cellular barcode sequencing data.

The instructions for processing scRNASeq barcodes can be found in the document titled "Barcode_in_scRNASeq.pdf".

Part1: CellBarcode

What's this package used for?

Cellular DNA barcoding (genetic lineage tracing) is a powerful tool for lineage tracing and clonal tracking studies. This package provides a toolbox for DNA barcode analysis, from extraction from fastq files to barcode error correction and quantification.

What types of barcodes can this package handle?

The package can handle all kinds of barcodes, as long as the barcodes have a pattern that can be matched by a regular expression, and each barcode is within a single sequencing read. It can handle barcodes with flexible length, and barcodes with UMI (unique molecular identifier).

This tool can also be used for the pre-processing part of amplicon data analysis such as CRISPR gRNA screening, immune repertoire sequencing, and meta genome data.

What can the package do?

The package provides functions for 1). Sequence quality control and filtering, 2). Barcode (and UMI) extraction from sequencing reads, 3). Sample and barcode management with metadata, 4). Barcode filtering.

The CPP codes improved the efficiency of the barcode extraction and filtering steps. Additionally, it is recommended to perform sequence quality control using a bash command tool for optimal efficiency.

About function naming

The functions in this package have names with `bc_` as initiation, except few exceptions. We hope it can facilitate the syntax auto-complement function of RStudio or IDE-like tools such as VS Code, VIM, and Emacs. By typing `bc_` you get function suggestions, and then you choose the function you need without exactly remembering them.

About the test data set

The test data set in this package can be accessed by

```
system.file("extdata", "mef_test_data", package="CellBarcode")
```

The data are from Jos et. al. There are 7 mouse embryo fibroblast (MEF) cell lines, each having a unique known VDJ barcode sequence. These MEF lines were mixed in a ratio of 1:2:4:8:16:32:64 to mimic multiple labeled clones who have different clone sizes.

sequence	clone size 2^x
AAGTCCAGTTCTACTATCGTAGCTACTA	1
AAGTCCAGTATCGTTACGCTACTA	2
AAGTCCAGTCTACTATCGTTACGACAGCTACTA	3
AAGTCCAGTTCTACTATCGTTACGAGCTACTA	4
AAGTCCATCGTAGCTACTA	5
AAGTCCAGTACTGTAGCTACTA	6
AAGTCCAGTACTATCGTACTA	7

Then 5 pools of 196 to 50000 cells were prepared from the MEF lines mixture. For each pool 2 technical replicates (NGS libraries) were prepared and sequenced, finally resulting in 10 samples.

sample name	cell number	replication
195_mixa	195	mixa
195_mixb	195	mixb
781_mixa	781	mixa
781_mixb	781	mixb
3125_mixa	3125	mixa
3125_mixb	3125	mixb

sample name	cell number	replication
12500_mixa	12500	mixa
12500_mixb	12500	mixb
50000_mixa	50000	mixa
50000_mixb	50000	mixb

The original FASTQ files are relatively large, so only 2000 reads for each sample have been randomly sampled as a test set here.

```
example_data <- system.file("extdata", "mef_test_data", package = "CellBarcode")
fq_files <- dir(example_data, "fastq.gz", full=TRUE)

# prepare metadata for the samples
metadata <- stringr::str_split_fixed(basename(fq_files), "_", 10)[, c(4, 6)]
metadata <- as.data.frame(metadata)
sample_name <- apply(metadata, 1, paste, collapse = "_")
colnames(metadata) = c("cell_number", "replication")
# metadata should has the row names consistent to the sample names
rownames(metadata) = sample_name
metadata
#>            cell_number replication
#> 50000_mixa       50000        mixa
#> 195_mixb           195        mixb
#> 50000_mixb       50000        mixb
#> 12500_mixa       12500        mixa
#> 12500_mixb       12500        mixb
#> 3125_mixa         3125        mixa
#> 3125_mixb         3125        mixb
#> 781_mixa           781        mixa
#> 781_mixb           781        mixb
#> 195_mixa           195        mixa
```

Installation

Install from Bioconductor.

```
if(!requireNamespace("BiocManager", quietly = TRUE))
    install.packages("BiocManager")
BiocManager::install("CellBarcode")
```

Install the development version from GitHub.

```
# install.packages("remotes")
remotes::install_github("wenjie1991/CellBarcode")
```

A basic workflow

Here is an example of a basic workflow:

```
# install.packages("stringr")
library(CellBarcode)
library(magrittr)

# The example data is the mix of MEF lines with known barcodes
```

```r
# 2000 reads for each file have been sampled for this test dataset
# extract UMI barcode with regular expression
bc_obj <- bc_extract(
  fq_files,
  pattern = "([ACGT]{12})CTCGAGGTCATCGAAGTATC([ACGT]+)CCGTAGCAAGCTCGAGAGTAGACCTACT",
  pattern_type = c("UMI" = 1, "barcode" = 2),
  sample_name = sample_name,
  metadata = metadata
)
bc_obj
#> Bonjour le monde, This is a BarcodeObj.
#> ----------
#> It contains:
#> ----------
#> @metadata: 4 field(s) available:
#> cell_number  replication  raw_read_count  barcode_read_count
#> ----------
#> @messyBc: 10 sample(s) for raw barcodes:
#>     In sample $50000_mixa there are: 1310 Tags
#>     In sample $195_mixb there are: 1318 Tags
#>     In sample $50000_mixb there are: 1385 Tags
#>     In sample $12500_mixa there are: 1321 Tags
#>     In sample $12500_mixb there are: 1361 Tags
#>     In sample $3125_mixa there are: 1287 Tags
#>     In sample $3125_mixb there are: 1297 Tags
#>     In sample $781_mixa there are: 1295 Tags
#>     In sample $781_mixb there are: 1303 Tags
#>     In sample $195_mixa there are: 1343 Tags

# sample subset operation, select technical repeats 'mixa'
bc_sub = bc_subset(bc_obj, sample=replication == "mixa")
bc_sub
#> Bonjour le monde, This is a BarcodeObj.
#> ----------
#> It contains:
#> ----------
#> @metadata: 4 field(s) available:
#> cell_number  replication  raw_read_count  barcode_read_count
#> ----------
#> @messyBc: 5 sample(s) for raw barcodes:
#>     In sample $50000_mixa there are: 1310 Tags
#>     In sample $12500_mixa there are: 1321 Tags
#>     In sample $3125_mixa there are: 1287 Tags
#>     In sample $781_mixa there are: 1295 Tags
#>     In sample $195_mixa there are: 1343 Tags

# filter the barcode, UMI barcode amplicon >= 2 & UMI counts >= 2
bc_sub <- bc_cure_umi(bc_sub, depth = 2) %>% bc_cure_depth(depth = 2)

# select barcodes with a white list
bc_2df(bc_sub)
#>    sample_name                      barcode_seq count
#> 1   50000_mixa         AAGTCCAGTATCGTTACGCTACTA    10
```

```
#> 2   50000_mixa           AAGTCCAGTACTGTAGCTACTA    11
#> 3   50000_mixa              AAGTCCATCGTAGCTACTA     3
#> 4   12500_mixa           AAGTCCAGTACTGTAGCTACTA    11
#> 5   12500_mixa         AAGTCCAGTATCGTTACGCTACTA    20
#> 6   12500_mixa              AAGTCCATCGTAGCTACTA     4
#> 7   12500_mixa AAGTCCAGTTCTACTATCGTTACGAGCTACTA     3
#> 8    3125_mixa              AAGTCCATCGTAGCTACTA     7
#> 9    3125_mixa         AAGTCCAGTATCGTTACGCTACTA    17
#> 10   3125_mixa AAGTCCAGTTCTACTATCGTTACGAGCTACTA     7
#> 11   3125_mixa           AAGTCCAGTACTGTAGCTACTA     9
#> 12    781_mixa         AAGTCCAGTATCGTTACGCTACTA     7
#> 13    781_mixa           AAGTCCAGTACTGTAGCTACTA     9
#> 14    781_mixa              AAGTCCATCGTAGCTACTA     2
#> 15    195_mixa              AAGTCCATCGTAGCTACTA     9
#> 16    195_mixa           AAGTCCAGTACTGTAGCTACTA    11
#> 17    195_mixa         AAGTCCAGTATCGTTACGCTACTA    12
#> 18    195_mixa AAGTCCAGTTCTACTATCGTTACGAGCTACTA     4
#> 19    195_mixa            AAGTCCAGTACTATCGTACTA     2
bc_sub[c("AAGTCCAGTACTATCGTACTA", "AAGTCCAGTACTGTAGCTACTA"), ]
#> Bonjour le monde, This is a BarcodeObj.
#> ----------
#> It contains:
#> ----------
#> @metadata: 5 field(s) available:
#> cell_number  replication  raw_read_count  barcode_read_count  depth_cutoff
#> ----------
#> @messyBc: 5 sample(s) for raw barcodes:
#>     In sample $50000_mixa there are: 434 Tags
#>     In sample $12500_mixa there are: 490 Tags
#>     In sample $3125_mixa there are: 455 Tags
#>     In sample $781_mixa there are: 524 Tags
#>     In sample $195_mixa there are: 484 Tags
#> ----------
#> @cleanBc: 5 samples for cleaned barcodes
#>     In sample $50000_mixa there are: 1 barcodes
#>     In sample $12500_mixa there are: 1 barcodes
#>     In sample $3125_mixa there are: 1 barcodes
#>     In sample $781_mixa there are: 1 barcodes
#>     In sample $195_mixa there are: 2 barcodes

# export the barcode counts to data.frame
head(bc_2df(bc_sub))
#>   sample_name              barcode_seq count
#> 1  50000_mixa AAGTCCAGTATCGTTACGCTACTA    10
#> 2  50000_mixa   AAGTCCAGTACTGTAGCTACTA    11
#> 3  50000_mixa      AAGTCCATCGTAGCTACTA     3
#> 4  12500_mixa   AAGTCCAGTACTGTAGCTACTA    11
#> 5  12500_mixa AAGTCCAGTATCGTTACGCTACTA    20
#> 6  12500_mixa      AAGTCCATCGTAGCTACTA     4

# export the barcode counts to matrix
head(bc_2matrix(bc_sub))
#>                                  X12500_mixa X195_mixa X3125_mixa X50000_mixa
↪  X781_mixa
```

```
#> AAGTCCAGTACTATCGTACTA                            0          2          0           0
↪  0
#> AAGTCCAGTACTGTAGCTACTA                          11         11          9          11
↪  9
#> AAGTCCAGTATCGTTACGCTACTA                        20         12         17          10
↪  7
#> AAGTCCAGTTCTACTATCGTTACGAGCTACTA                 3          4          7           0
↪  0
#> AAGTCCATCGTAGCTACTA                              4          9          7           3
↪  2
```

Sequence quality control

Evaluation

In a full analysis starting from fastq files, the first step is to check the sequencing quality and filter as required. The `bc_seq_qc` function is for checking the sequencing quality. If multiple samples are input the output is a `BarcodeQcSet` object, otherwise a `BarcodeQC` object will be returned. In addition, `bc_seq_qc` also can handle the `ShortReadQ`, `DNAStringSet,` and other data types.

```
qc_noFilter <- bc_seq_qc(fq_files)
qc_noFilter
#> The sequence QC set, use `[]` to select sample:
#>      5290_1_BCM_50000_mef_mixa_GTTCTCC_S2_R1_001.fastq.gz
#>      5290_10_BCM_195_mef_mixb_GTCATTG_S11_R1_001.fastq.gz
#>      5290_2_BCM_50000_mef_mixb_GATGTGT_S5_R1_001.fastq.gz
#>      5290_3_BCM_12500_mef_mixa_TGCCTTG_S4_R1_001.fastq.gz
#>      5290_4_BCM_12500_mef_mixb_TAACTGC_S8_R1_001.fastq.gz
#>      5290_5_BCM_3125_mef_mixa_GCTTCCA_S9_R1_001.fastq.gz
#>      5290_6_BCM_3125_mef_mixb_TGTGAGT_S7_R1_001.fastq.gz
#>      5290_7_BCM_781_mef_mixa_CCTTACC_S12_R1_001.fastq.gz
#>      5290_8_BCM_781_mef_mixb_CGTATCC_S13_R1_001.fastq.gz
#>      5290_9_BCM_195_mef_mixa_GTACTGT_S14_R1_001.fastq.gz
bc_names(qc_noFilter)
#>  [1] "5290_1_BCM_50000_mef_mixa_GTTCTCC_S2_R1_001.fastq.gz"
↪  "5290_10_BCM_195_mef_mixb_GTCATTG_S11_R1_001.fastq.gz"
↪  "5290_2_BCM_50000_mef_mixb_GATGTGT_S5_R1_001.fastq.gz"
↪  "5290_3_BCM_12500_mef_mixa_TGCCTTG_S4_R1_001.fastq.gz"
↪  "5290_4_BCM_12500_mef_mixb_TAACTGC_S8_R1_001.fastq.gz"
#>  [6] "5290_5_BCM_3125_mef_mixa_GCTTCCA_S9_R1_001.fastq.gz"
↪  "5290_6_BCM_3125_mef_mixb_TGTGAGT_S7_R1_001.fastq.gz"
↪  "5290_7_BCM_781_mef_mixa_CCTTACC_S12_R1_001.fastq.gz"
↪  "5290_8_BCM_781_mef_mixb_CGTATCC_S13_R1_001.fastq.gz"
↪  "5290_9_BCM_195_mef_mixa_GTACTGT_S14_R1_001.fastq.gz"
class(qc_noFilter)
#> [1] "BarcodeQcSet"
#> attr(,"package")
#> [1] "CellBarcode"
```

The `bc_plot_seqQc` function can be invoked with a `BarcodeQcSet` as an argument, and the output is a QC summary with two panels. The first shows the ratio of ATCG bases for each sequencing cycle with one sample per row; this allows the user to, for example, identify constant or random parts of the sequencing read. The second figure shows the average sequencing quality index of each cycle (base).

For the test set, the first 12 bases are UMI, which are random. This is followed by the constant region of

the barcode (the PCR primer selects reads with this sequence), and here we observe a specific base for each cycle across all the samples.

```
bc_plot_seqQc(qc_noFilter)
```

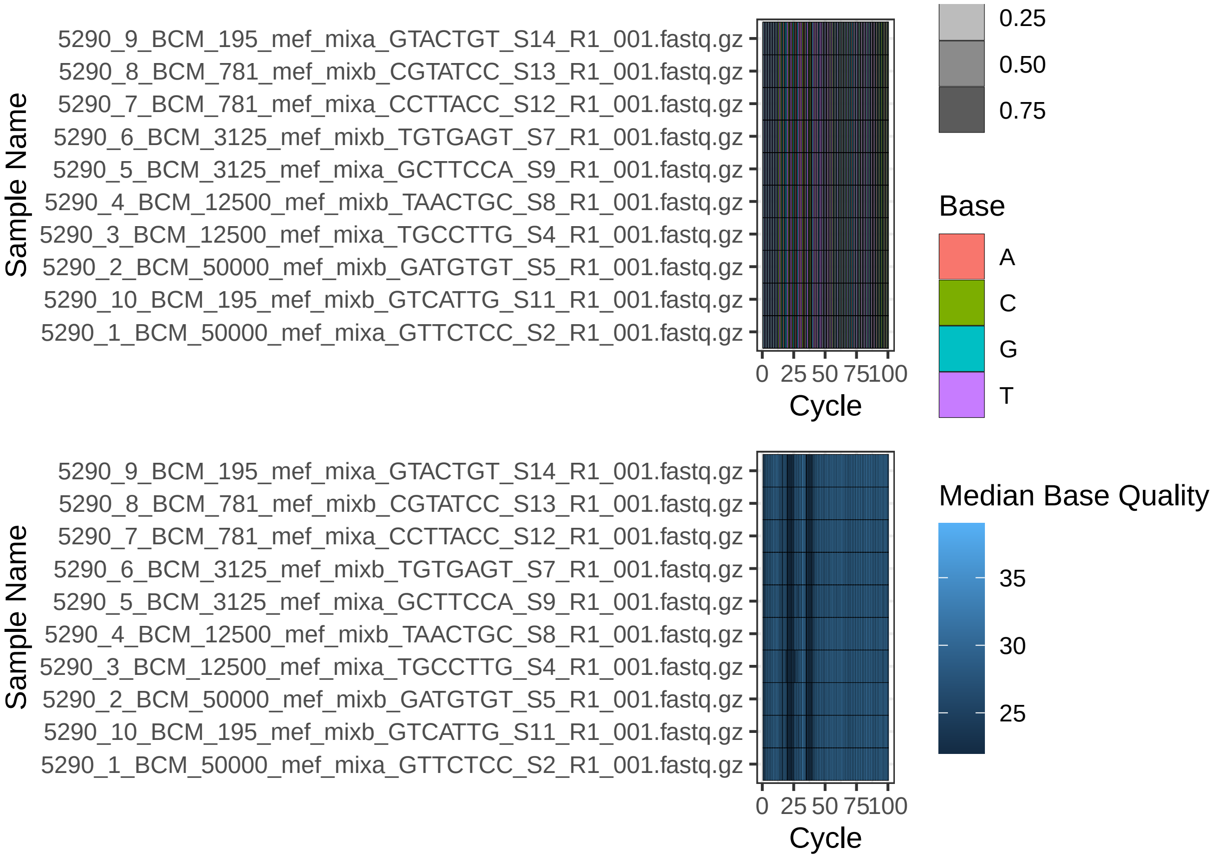

We can also plot one of the `BarcodeQc` in the `BarcodeQcSet` object. In the output, there are three panels. The top left one shows the reads depth distribution, the top right figure shows the "ATCG" base ratio by each sequencing cycle, and the last one shows the sequencing quality by sequencing cycle.

```
qc_noFilter[1]
#> Sequnece QC, summary:
#>      total_read: 2000
#>      p5_read_length: 100
#>      median_read_length: 100
#>      p95_read_length: 100
class(qc_noFilter[1])
#> [1] "BarcodeQc"
#> attr(,"package")
#> [1] "CellBarcode"
bc_plot_seqQc(qc_noFilter[1])
```

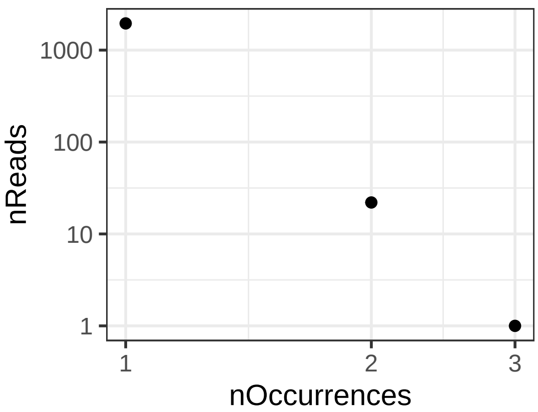

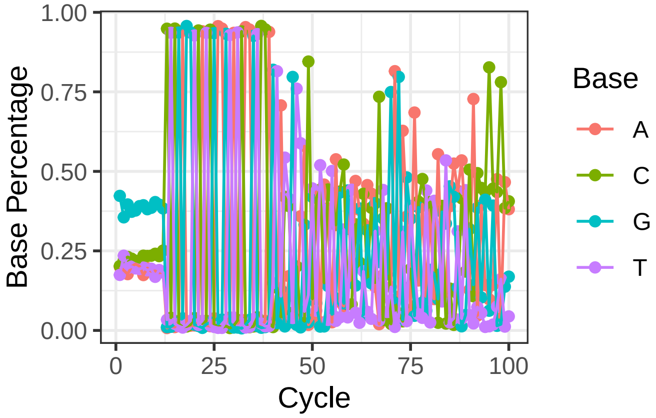

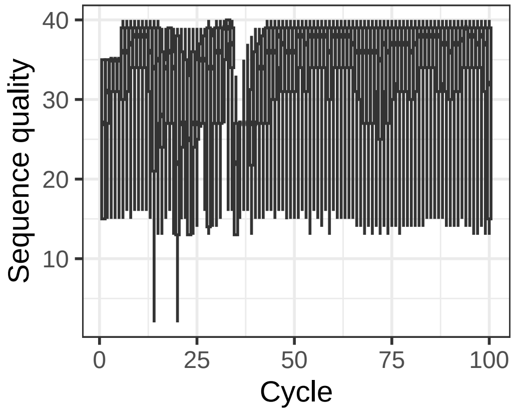

Filtering

`bc_seq_filter` reads in the sequence data and applies filters, then returns a `ShortReadQ` object that contains the filtered sequences.

The `bc_seq_filter` function can read fastq files, and it can also handle sequencing reads in `ShortReadQ`, `DNAStringSet`, and `data.frame`.

The currently available filter parameters are: - min_average_quality: average base sequencing quality across the read. - min_read_length: minimum number of bases per read. - N_threshold: maximum number of "N" bases in sequence.

```
fq_filter <- bc_seq_filter(
  fq_files,
  min_average_quality = 30,
  min_read_length = 60,
  sample_name = sample_name)

fq_filter
#> $`50000_mixa`
#> class: ShortReadQ
#> length: 1154 reads; width: 100 cycles
#>
#> $`195_mixb`
#> class: ShortReadQ
#> length: 1187 reads; width: 100 cycles
#>
#> $`50000_mixb`
```

```
#> class: ShortReadQ
#> length: 1234 reads; width: 100 cycles
#>
#> $`12500_mixa`
#> class: ShortReadQ
#> length: 1186 reads; width: 100 cycles
#>
#> $`12500_mixb`
#> class: ShortReadQ
#> length: 1238 reads; width: 100 cycles
#>
#> $`3125_mixa`
#> class: ShortReadQ
#> length: 1100 reads; width: 100 cycles
#>
#> $`3125_mixb`
#> class: ShortReadQ
#> length: 1146 reads; width: 100 cycles
#>
#> $`781_mixa`
#> class: ShortReadQ
#> length: 1154 reads; width: 100 cycles
#>
#> $`781_mixb`
#> class: ShortReadQ
#> length: 1164 reads; width: 100 cycles
#>
#> $`195_mixa`
#> class: ShortReadQ
#> length: 1205 reads; width: 100 cycles
bc_plot_seqQc(bc_seq_qc(fq_filter))
```

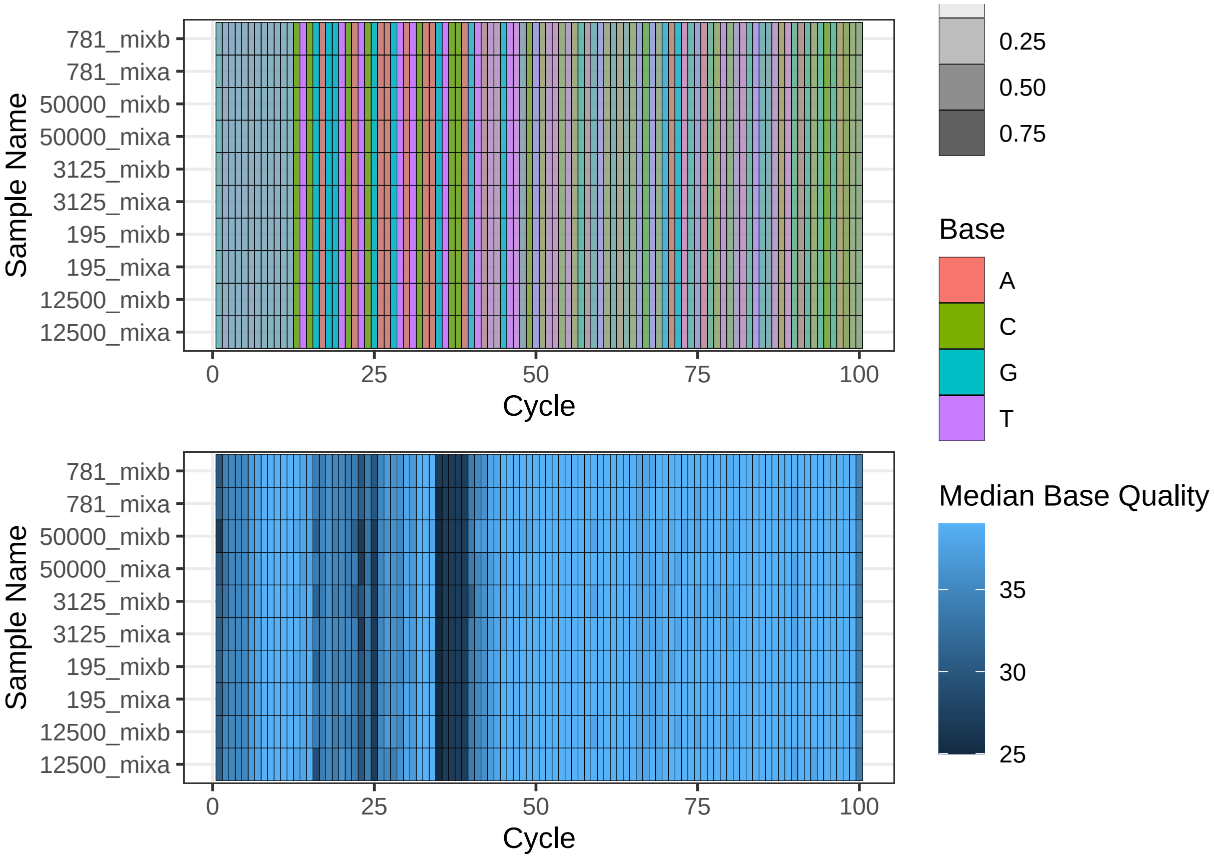

Parse reads

One of the core applications of this package is parsing the sequences to get the barcode (and UMI). Our package uses regular expressions to identify barcodes (and UMI) from sequencing reads. This is how we tell `bc_extract` the structure of the input sequences.

3 arguments are necessary for `bc_extract`, they are: - x: the sequence data, it can be in fastq, `ShortReadQ`, `DNAStringSet`, or `data.frame` format. - pattern: the sequence pattern regular expression. - pattern_type: pattern description.

The `pattern` argument is the regular expression, it tells the function where to find the barcode (or UMI). We capture the barcode (or UMI) by `()` in the backbone. For the sequence captured by `()`, the `pattern_type` argument tells which is the UMI or the barcode. In the example

```
pattern <- "([ACGA]{12})CTCGAGGTCATCGAAGTATC([ACGT]+)CCGTAGCAAGCTCGAGAGTAGACCTACT"
pattern_type <- c("UMI" = 1, "barcode" = 2)
```

1. The sequence starts with 12 base pairs of random sequences, which is UMI. It is the first barcode captured by `()` in the `pattern` argument and corresponds to `UMI = 1` in the `pattern_type` argument.
2. Then, there is a known constant sequence: `CTCGAGGTCATCGAAGTATC`.
3. Following the constant region, there is a flexible length random sequence. This is the barcode which is trapped by second `()`, and it is defined by `barcode = 2` in the `pattern_type` argument.
4. At the end of the sequence, there is another constant sequence `CCGTAGCAAGCTCGAGAGTAGACCTACT`.

In the regular expression, the UMI pattern is retrieved with `[ACGT]{12}`. The `[ACGT]` means to match "A", "C", "G" or "T", and the `{12}` means match 12 `[ACGT]`. In the barcode pattern `[ACGT]+`, again `[ACGT]` means match "A", "C", "G" or "T" and the `+` says to match at least one `[ACGT]`.

The `bc_extract` function is used to extract the barcode(s) from the sequences. It returns a `BarcodeObj`

object if the input is a list or a vector of Fastq files. The `BarcodeObj` created by `bc_extract` is a R S4 class with three slots: `messyBc`, `metadata`, and `cleanBc` (which is NULL in the `bc_extract` output). They can be accessed by `@` operator or corresponding accessors:

- `bc_messyBc`: return the `messyBc` slot.
- `bc_cleanBc`: return the `cleanBc` slot.
- `bc_meta`: return the `metadata` slot.

`messyBc` is a list, where each element is a `data.table` corresponding to the successive samples. Each `data.table` has 3 columns:

1. umi_seq (optional): UMI sequence, applicable when there is a UMI in `pattern` and `pattern_type` argument.
2. barcode_seq: barcode sequence.
3. count: the count of the full read sequence.

Attention: In the `data.table`, `barcode_seq` value may be not unique, as two different full read sequences can contain the same barcode sequence, due to the UMI or mutations in the constant region.

If the input to `bc_extract` is just a sample, the output is a single `data.frame` with the 3 columns 1). `umi_seq`, 2). `barcode_seq` and 3). `count`, as described above.

The sequence in `match_seq` is a contiguous segment of the full read given in `reads_seq`. The `umi_seq` and `barcode_seq` are contiguous segments of `match_seq`. Take note that, the `reads_seq` is the unique id for each row. The `match_seq`, `umi_seq`, or `barcode_seq` can be duplicated, due to the potential variation in the region outside of `match_seq`. Please keep this in mind when you use data in `$messyBc` to perform the analysis.

Sequencing without UMI

In the following example, only a barcode is extracted.

```
pattern <- "CTCGAGGTCATCGAAGTATC([ACGT]+)CCGTAGCAAGCTCGAGAGTAGACCTACT"
bc_obj <- bc_extract(
  fq_filter,
  sample_name = sample_name,
  pattern = pattern,
  pattern_type = c(barcode = 1))

bc_obj
#> Bonjour le monde, This is a BarcodeObj.
#> ----------
#> It contains:
#> ----------
#> @metadata: 2 field(s) available:
#> raw_read_count  barcode_read_count
#> ----------
#> @messyBc: 10 sample(s) for raw barcodes:
#>     In sample $50000_mixa there are: 39 Tags
#>     In sample $195_mixb there are: 31 Tags
#>     In sample $50000_mixb there are: 49 Tags
#>     In sample $12500_mixa there are: 37 Tags
#>     In sample $12500_mixb there are: 44 Tags
#>     In sample $3125_mixa there are: 36 Tags
#>     In sample $3125_mixb there are: 44 Tags
#>     In sample $781_mixa there are: 34 Tags
#>     In sample $781_mixb there are: 29 Tags
#>     In sample $195_mixa there are: 34 Tags
```

```r
names(bc_messyBc(bc_obj)[[1]])
#> [1] "barcode_seq" "count"
```

Here the regular expression matches a constant sequence at the beginning and the end and the barcode in `()` matches at least one of any character.

Sequencing with UMI

In the following example, both UMI and barcode are extracted. The regular expression is explained above.

```r
pattern <- "([ACGA]{12})CTCGAGGTCATCGAAGTATC([ACGT]+)CCGTAGCAAGCTCGAGAGTAGACCTACT"
bc_obj_umi <- bc_extract(
  fq_filter,
  sample_name = sample_name,
  pattern = pattern,
  maxLDist = 0,
  pattern_type = c(UMI = 1, barcode = 2))

class(bc_obj_umi)
#> [1] "BarcodeObj"
#> attr(,"package")
#> [1] "CellBarcode"
bc_obj_umi
#> Bonjour le monde, This is a BarcodeObj.
#> ----------
#> It contains:
#> ----------
#> @metadata: 2 field(s) available:
#> raw_read_count  barcode_read_count
#> ----------
#> @messyBc: 10 sample(s) for raw barcodes:
#>     In sample $50000_mixa there are: 142 Tags
#>     In sample $195_mixb there are: 127 Tags
#>     In sample $50000_mixb there are: 143 Tags
#>     In sample $12500_mixa there are: 106 Tags
#>     In sample $12500_mixb there are: 134 Tags
#>     In sample $3125_mixa there are: 106 Tags
#>     In sample $3125_mixb there are: 120 Tags
#>     In sample $781_mixa there are: 131 Tags
#>     In sample $781_mixb there are: 112 Tags
#>     In sample $195_mixa there are: 154 Tags
```

Metadata updated

`bc_extract` added two columns named "row_read_count" and "barcode_read_count" to the metadata slot of the returned BarcodeObj object.

row_read_count: Total raw reads number of each sample. **barcode_read_count**: The number of reads that contain the barcodes.

You can use the ratio of `barcode_read_count` versus `raw_read_count` to check the successfulness of the sequencing or correctness of the pattern given to the `bc_extract`.

```r
# select two samples from bc_obj_umi
bc_obj_umi_sub <- bc_obj_umi[, c("781_mixa", "781_mixb")]
```

```r
# get the metadata matrix
(d <- bc_meta(bc_obj_umi_sub))
#>          raw_read_count barcode_read_count
#> 781_mixa           1154                134
#> 781_mixb           1164                112
# use the row name of the metadata, which contains the sample names
d$sample_name <- rownames(d)

d$barcode_read_count / d$raw_read_count
#> [1] 0.11611785 0.09621993
# visualize
ggplot(d) +
    aes(x=sample_name, y=barcode_read_count / raw_read_count) +
    geom_bar(stat="identity")
```

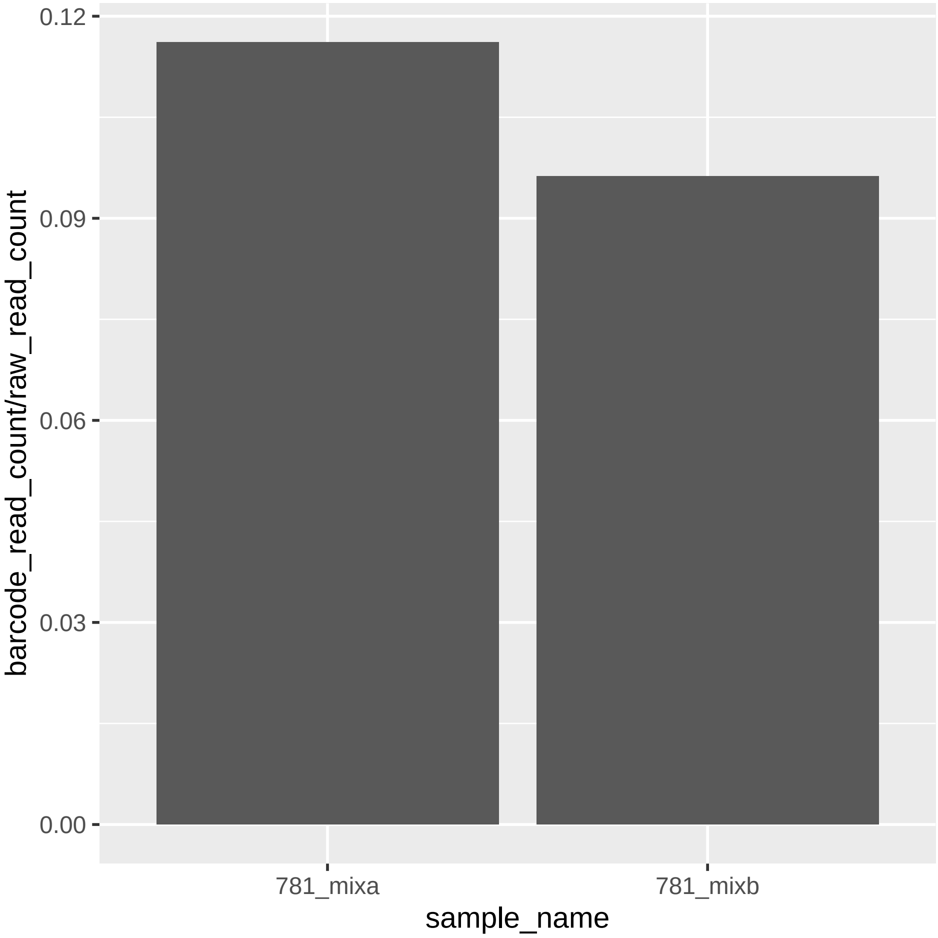

Data management

Besides, we provide operators to handle the barcodes and samples in `BarcodeObj` object. You can easily select one or several samples by their names, indices or metadata.

Select slot by accessors:

```r
# Access messyBc slot
head(bc_messyBc(bc_obj_umi)[[1]], n=2)
```

```
#>         umi_seq               barcode_seq count
#> 1 GGACAGAGCGGA AAGTCCAGTATCGTTACGCTACTA     2
#> 2 GGCCGGCGGGGC   AAGTCCAGTACTGTAGCTACTA     2
# return a data.frame
head(bc_messyBc(bc_obj_umi, isList=FALSE), n=2)
#>    sample_name      umi_seq              barcode_seq count
#> 1:  50000_mixa GGACAGAGCGGA AAGTCCAGTATCGTTACGCTACTA     2
#> 2:  50000_mixa GGCCGGCGGGGC   AAGTCCAGTACTGTAGCTACTA     2

# Access cleanBc slot
# return a data.frame
head(bc_cleanBc(bc_obj_umi, isList=FALSE), n=2)
#>    sample_nmae      umi_seq              barcode_seq count
#> 1:  50000_mixa GGACAGAGCGGA AAGTCCAGTATCGTTACGCTACTA     2
#> 2:  50000_mixa GGCCGGCGGGGC   AAGTCCAGTACTGTAGCTACTA     2
```

Select sample by sample names

```
bc_obj_umi_sub <- bc_obj_umi[, c("781_mixa", "781_mixb")]
bc_names(bc_obj_umi_sub)
#> [1] "781_mixa" "781_mixb"
```

Set metadata

```
bc_meta(bc_obj_umi_sub)$rep <- c("a", "b")
bc_meta(bc_obj_umi_sub)
#>          raw_read_count barcode_read_count rep
#> 781_mixa           1154                134   a
#> 781_mixb           1164                112   b
```

Select sample by metadata

```
bc_subset(bc_obj_umi_sub, sample = rep == "a")
#> Bonjour le monde, This is a BarcodeObj.
#> ----------
#> It contains:
#> ----------
#> @metadata: 3 field(s) available:
#> raw_read_count  barcode_read_count  rep
#> ----------
#> @messyBc: 1 sample(s) for raw barcodes:
#>     In sample $781_mixa there are: 131 Tags
```

Barcode filtering

Most of the time, it needs PCR and NGS to read out the cellular barcode sequences. `bc_extract` will output all barcodes found in the sequences. Some of the identified barcodes may contain PCR or sequencing errors.

The potential errors derived from PCR and NGS lead to spurious barcodes that do not exist in biological samples. The spurious barcodes are more likely to be less abundant compared to the corresponding "mother" barcodes they derived from.

As UMI can be used to label a DNA molecular, one UMI-labeled barcode molecular becomes multiple copies by PCR. Thus all the sequences derived from the template sequence, including the original template sequence and mutant ones, are marked by UMI for having the same UMI. The original template sequence is likely to

have more reads compared to the spurious one derived from PCR or sequencing mutation, as errors happen with low probability. Also, a barcode sequence is less likely to be spurious when it relates to several UMIs.

We created the `bc_cure_*` functions to perform filtering for removing the potential spurious barcodes. The `bc_cure_*` functions create or update the `cleanBc` slot in `BarcodeObj`. The `cleanBc` slot contains 2 columns - barcode_seq: barcode sequence. - counts: reads count, or UMI count in the case that the cleanBc was created by `bc_cure_umi`.

Important: In the `createBc` slot, the barcode_seq is not duplicated in each sample.

In the `bc_cure_*` function family, there are `bc_cure_depth`, `bc_cure_umi`, and `bc_cure_cluster`.

Filter UMI-barcode tag

In the case when the UMI is applied, the template sequence is marked by UMI, and we use "UMI-barcode tag" to denote a combination of a UMI and a barcode. The UMI-barcode tag with few reads is likely deriving from PCR or sequence errors. `bc_cure_umi` carries out the filtering based on the UMI-barcode tag read count from the `messyBc` slot in BarcodeObj object, and returns an updated BarcodeObj object with a `cleanBc` slot containing the barcodes passing the filtering.

```
# Filter the barcodes with UMI-barcode tag >= 1,
# and treat UMI as absolute unique and do "fish"
bc_obj_umi_sub <- bc_cure_umi(
    bc_obj_umi_sub, depth = 1,
    isUniqueUMI = TRUE,
    doFish = TRUE)
bc_obj_umi_sub
#> Bonjour le monde, This is a BarcodeObj.
#> ----------
#> It contains:
#> ----------
#> @metadata: 3 field(s) available:
#> raw_read_count  barcode_read_count  rep
#> ----------
#> @messyBc: 2 sample(s) for raw barcodes:
#>     In sample $781_mixa there are: 131 Tags
#>     In sample $781_mixb there are: 112 Tags
#> ----------
#> @cleanBc: 2 samples for cleaned barcodes
#>     In sample $781_mixa there are: 9 barcodes
#>     In sample $781_mixb there are: 8 barcodes
```

The available arguments of `bc_cure_umi` are:

- depth: minimum read count required for a UMI.
- doFish: if true, for barcodes with UMI read depth above the threshold, "fish" for identical barcodes with UMI read depth below the threshold. The consequence of "doFish" will not increase the number of identified barcodes, but the UMI counts will increase due to including the low-depth UMI barcodes.
- isUniqueUMI: One UMI sequence may be linked to several barcodes. Do you believe the UMI is absolutely unique? If yes, we treat the UMI as absolutely unique makers. Thus the most abundant barcode will be picked for a UMI, and less abundant barcodes with the same UMI are obsolete.

Filter by count

`bc_cure_depth` performs filtering by reads/UMI count. It can filter the raw barcodes in the `messyBc` and create a `cleanBc` slot, or update the `cleanBc` when the argument `isUpdate` is TRUE. You should set this

argument to `TRUE` when you want to apply the filtering on the UMI count with the `bc_cure_umi` output. In this case, `bc_cure_depth` will update the `cleanBc` slot created by `bc_cure_umi`.

The function has two arguments:

- depth: sequence/UMI count threshold, it can be a numeric number or a numeric vector, in the latter case, each number corresponds to a sample in the BarcodeObj object.
- isUpdate: if true (default) the bc_cure will preferentially perform filtering on the `cleanBc` slot and update it, otherwise the `messyBc` will be used as input.

```
# Apply the barcode sequence depth with depth >= 3
# With isUpdate = FLASE, the data in `messyBc` slot of bc_obj_umi_sub
#   will be used for depth filtering. The UMI information will be discarded,
#   the identical barcodes in different UMI-barcode tags are merged before
#   performing the sequence depth filtering.
bc_obj_umi_sub <- bc_cure_depth(bc_obj_umi_sub, depth = 3, isUpdate = FALSE)
bc_obj_umi_sub
#> Bonjour le monde, This is a BarcodeObj.
#> ----------
#> It contains:
#> ----------
#> @metadata: 4 field(s) available:
#> raw_read_count  barcode_read_count  rep  depth_cutoff
#> ----------
#> @messyBc: 2 sample(s) for raw barcodes:
#>     In sample $781_mixa there are: 131 Tags
#>     In sample $781_mixb there are: 112 Tags
#> ----------
#> @cleanBc: 2 samples for cleaned barcodes
#>     In sample $781_mixa there are: 5 barcodes
#>     In sample $781_mixb there are: 4 barcodes

# Apply the UMI count filter, keep barcode >= 3 UMI
# The `bc_cure_umi` function applies the filtering on the UMI-barcode tags,
#   and create a `cleanBc` slot in the return BarcodeObj object. Then,
#   the `bc_cure_depth` with `isUpdate` argument TRUE will apply the filtering
#   on the UMI counts in `cleanBc` and updated the `cleanBc`.
bc_obj_umi_sub <- bc_cure_umi(
    bc_obj_umi_sub, depth = 1,
    isUniqueUMI = TRUE,
    doFish = TRUE)
bc_obj_umi_sub
#> Bonjour le monde, This is a BarcodeObj.
#> ----------
#> It contains:
#> ----------
#> @metadata: 4 field(s) available:
#> raw_read_count  barcode_read_count  rep  depth_cutoff
#> ----------
#> @messyBc: 2 sample(s) for raw barcodes:
#>     In sample $781_mixa there are: 131 Tags
#>     In sample $781_mixb there are: 112 Tags
#> ----------
#> @cleanBc: 2 samples for cleaned barcodes
#>     In sample $781_mixa there are: 9 barcodes
```

```
#>     In sample $781_mixb there are: 8 barcodes
bc_obj_umi_sub <- bc_cure_depth(bc_obj_umi_sub, depth = 3, isUpdate = TRUE)
bc_obj_umi_sub
#> Bonjour le monde, This is a BarcodeObj.
#> ----------
#> It contains:
#> ----------
#> @metadata: 4 field(s) available:
#> raw_read_count  barcode_read_count  rep  depth_cutoff
#> ----------
#> @messyBc: 2 sample(s) for raw barcodes:
#>     In sample $781_mixa there are: 131 Tags
#>     In sample $781_mixb there are: 112 Tags
#> ----------
#> @cleanBc: 2 samples for cleaned barcodes
#>     In sample $781_mixa there are: 5 barcodes
#>     In sample $781_mixb there are: 4 barcodes
```

Cluster barcode by sequence similarity

The sequences with more reads have more chance to be the original templates. In contrast, the sequences with few reads are more likely derived from mutations of the most abundant sequence. Thus, the spurious sequence might be identified by comparing the most abundant sequence to the least one. If they are similar, the least abundant sequence will be removed.

`bc_cure_cluster` performs the clustering to remove the barcodes with insufficient depth (or UMI counts) compared to the most abundant ones with similarity, it needs the `cleanBc` slot and will update it.

To control the clustering methods and threshold for merging you need the following arguments:

- dist_thresh: a single integer or vector of integers with the length of sample number, specifying the editing distance threshold of merging two similar barcode sequences. If the input is a vector, each value in the vector is for one sample according to the sample order in BarcodeObj object.
- dist_method: A character string, specifying the distance algorithm for evaluating barcode similarity. It can be "hamm" for Hamming distance or "leven" for Levenshtein distance.
- cluster_method: A character string specifying the algorithm used to perform the clustering merging of barcodes. Currently only "greedy" is available, in this case, the least abundant barcode is removed.
- param count_threshold: An integer, read depth threshold to consider a barcode as a true barcode, when a barcode with a count higher than this threshold it will not be removed.
- dist_costs: A list, the cost of the events when calculating distance between two barcode sequences, applicable when Levenshtein distance is applied. The names of the vector have to be "insert", "delete" and "replace", specifying the weight of insertion, deletion, and replacement events respectively. The default cost for each event is 1.

```
# Do the clustering and merging the least abundant barcodes to the similar
# abundant ones
bc_cure_cluster(bc_obj_umi_sub)
#> Bonjour le monde, This is a BarcodeObj.
#> ----------
#> It contains:
#> ----------
#> @metadata: 4 field(s) available:
#> raw_read_count  barcode_read_count  rep  depth_cutoff
#> ----------
#> @messyBc: 2 sample(s) for raw barcodes:
```

```
#>     In sample $781_mixa there are: 131 Tags
#>     In sample $781_mixb there are: 112 Tags
#> ----------
#> @cleanBc: 2 samples for cleaned barcodes
#>     In sample $781_mixa there are: 5 barcodes
#>     In sample $781_mixb there are: 4 barcodes
```

Barcode count distribution

We provide `bc_plot_single`, `bc_plot_mutual`, and `bc_plot_pair` functions to help explore the barcode count distribution for a single sample or between two samples.

Single sample

`bc_plot_single` can be used for exploring barcode count distribution sample-wise. It uses the `cleanBc` slot in the BarcodeObj bc_obj_umi_sub.

```
bc_plot_single(bc_obj_umi_sub)
```

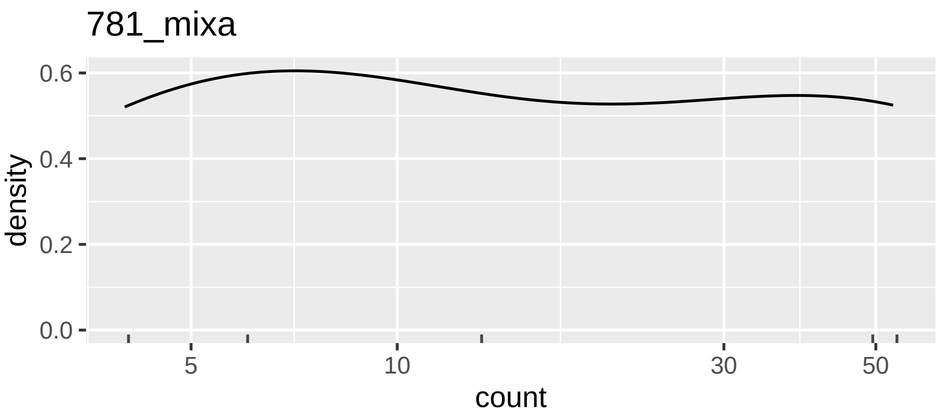

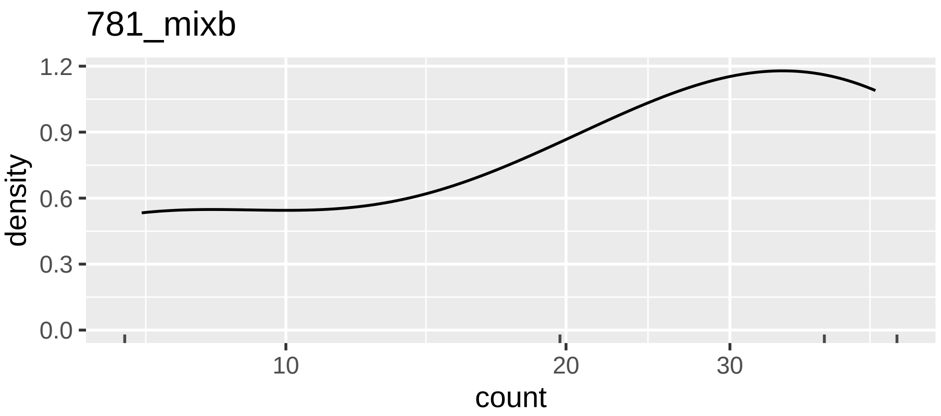

The `bc_plot_single` function provides arguments for showing the potential cutoff point and highlighting specific barcodes.

```
# re-do the filtering using depth threshold 0 to include all barcodes.
bc_obj_umi_sub_neo <- bc_cure_depth(bc_obj_umi_sub, depth=0, isUpdate=FALSE)

# you can use the count_marks argument to display the cutoff points in the figure
# and the highlight argument to highlight specific barcodes.
```

```r
bc_plot_single(bc_obj_umi_sub_neo, count_marks=10,
    highlight= c("AAGTCCAGTACTATCGTACTA", "AAGTCCAGTACTGTAGCTACTA"))
```

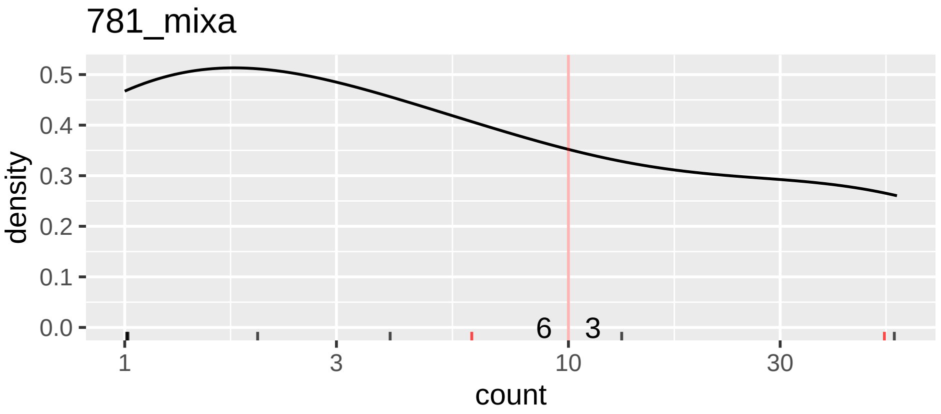

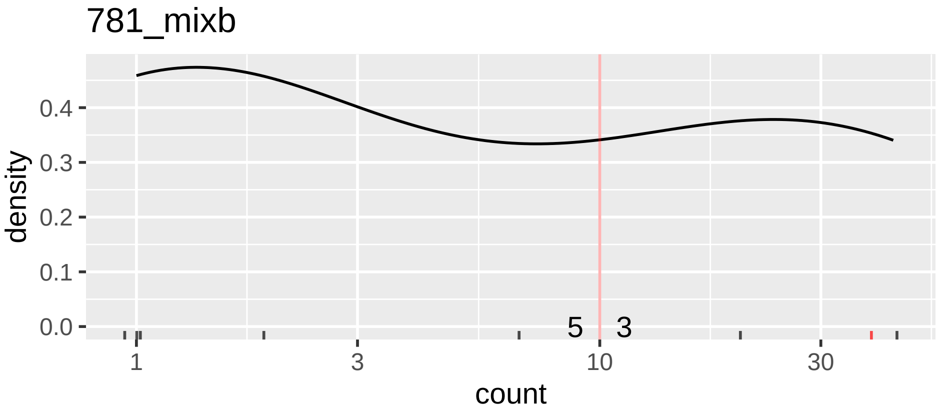

Pairwise

`bc_plot_mutual` and `bc_plot_pair` are designed for comparing the barcodes between two samples.

The `bc_plot_mutual` generates a scatter plot matrix that contains all the pairwise sample combinations in the provided BarcodeObj object.

```r
# create a new BarcodeObj for following visualization
# use depth as 0 to include all the barcodes.
bc_obj_umi_neo <- bc_cure_depth(bc_obj_umi[, 1:4], depth=0)
# you can set the count_marks to display the cutoff point
# and highlight specific barcodes dots by highlight
bc_plot_mutual(bc_obj_umi_neo, count_marks=c(10, 20, 30, 40),
    highlight= c("AAGTCCAGTACTATCGTACTA", "AAGTCCAGTACTGTAGCTACTA"))
```

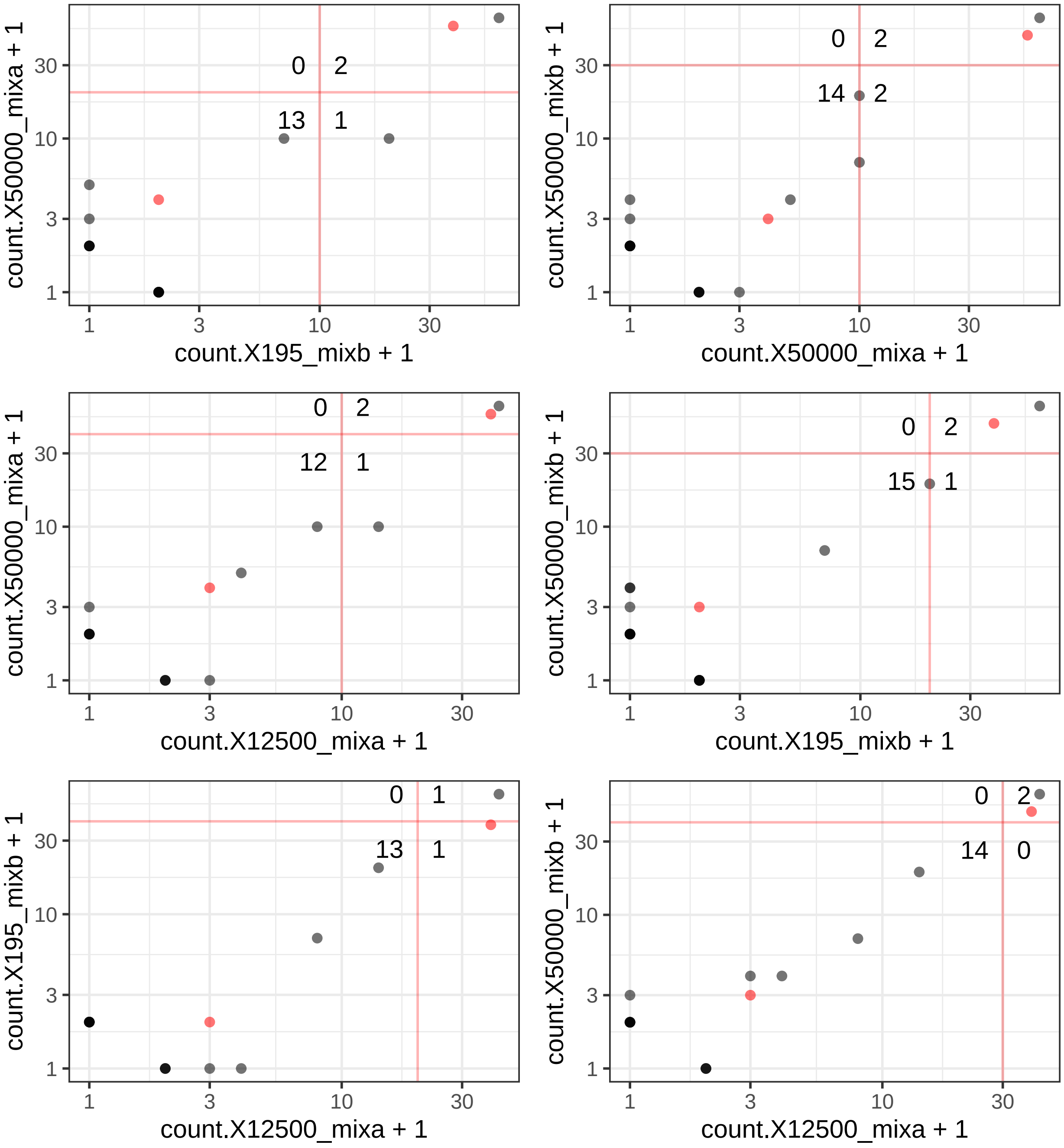

And the `bc_plot_pair` only draws the scatter plot for the given sample pairs.

```
# create a new BarcodeObj for following visualization
# use depth as 0 to include all the barcodes.
bc_obj_umi_neo <- bc_cure_depth(bc_obj_umi[, 1:4], depth=0)

# 2d scatters plot with x axis of sample_x and y axis of sample_y
# sample_x, and sample_y can be the sample name or sample index
bc_plot_pair(
    bc_obj_umi_neo,
    sample_x = c("50000_mixa"),
    sample_y = c("50000_mixb", "12500_mixa", "195_mixb"),
```

```
    count_marks_x = 10,
    count_marks_y = c(10, 20, 30),
    highlight= c("AAGTCCAGTACTATCGTACTA", "AAGTCCAGTACTGTAGCTACTA")
)
```

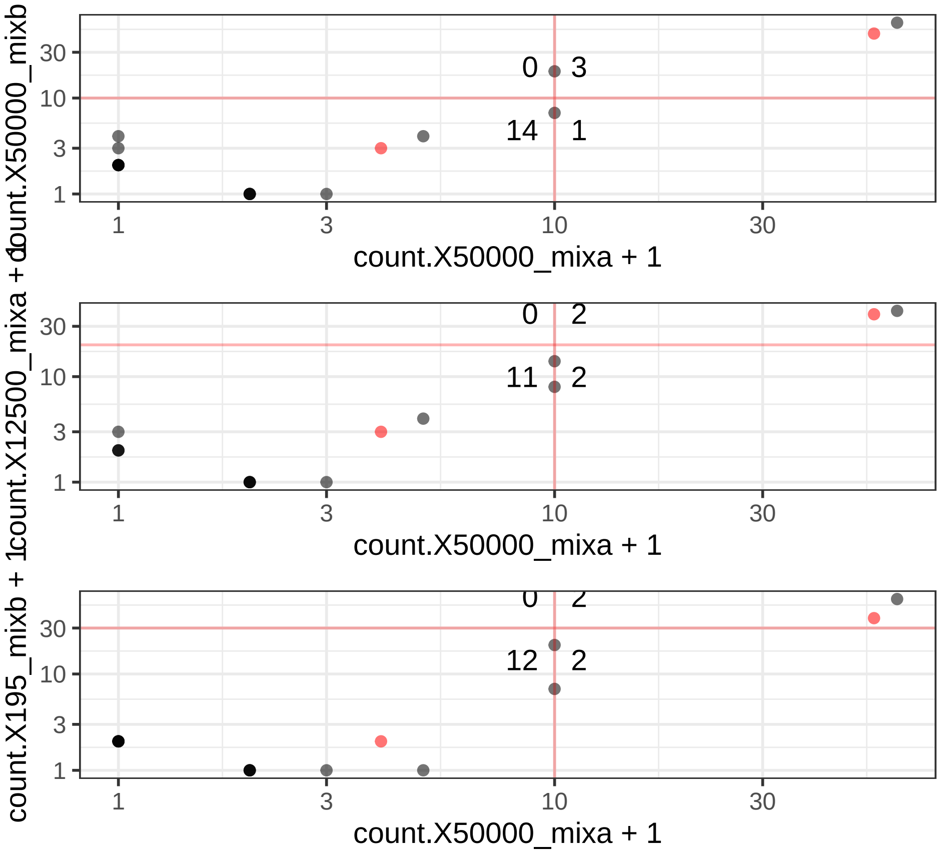

Miscellaneous

We provide functions to transform the barcode information in `BarcodeObj` to more general R data types.

Sample names

```
bc_names(bc_obj_umi_sub)
#> [1] "781_mixa" "781_mixb"
```

Output to data.frame

`bc_2df` function uses the barcode and count info in the `cleanBc` slot, and outputs a data.frame contains:

- barcode_seq: barcode sequence
- sample_name
- count: reads or UMI count

```
bc_2df(bc_obj_umi_sub)
#>   sample_name                barcode_seq count
#> 1    781_mixa      AAGTCCAGTATCGTTACGCTACTA    53
#> 2    781_mixa        AAGTCCAGTACTGTAGCTACTA    50
```

```
#> 3    781_mixa            AAGTCCAGTACTATCGTACTA     6
#> 4    781_mixa              AAGTCCATCGTAGCTACTA    13
#> 5    781_mixa AAGTCCAGTTCTACTATCGTTACGAGCTACTA     4
#> 6    781_mixb           AAGTCCAGTACTGTAGCTACTA    37
#> 7    781_mixb         AAGTCCAGTATCGTTACGCTACTA    43
#> 8    781_mixb              AAGTCCATCGTAGCTACTA    20
#> 9    781_mixb AAGTCCAGTTCTACTATCGTTACGAGCTACTA     7
```

Or if you prefer `data.table`

```
bc_2dt(bc_obj_umi_sub)
#>    sample_name                      barcode_seq count
#> 1:    781_mixa         AAGTCCAGTATCGTTACGCTACTA    53
#> 2:    781_mixa           AAGTCCAGTACTGTAGCTACTA    50
#> 3:    781_mixa            AAGTCCAGTACTATCGTACTA     6
#> 4:    781_mixa              AAGTCCATCGTAGCTACTA    13
#> 5:    781_mixa AAGTCCAGTTCTACTATCGTTACGAGCTACTA     4
#> 6:    781_mixb           AAGTCCAGTACTGTAGCTACTA    37
#> 7:    781_mixb         AAGTCCAGTATCGTTACGCTACTA    43
#> 8:    781_mixb              AAGTCCATCGTAGCTACTA    20
#> 9:    781_mixb AAGTCCAGTTCTACTATCGTTACGAGCTACTA     7
```

Output to matrix

`bc_2matrix` uses barcode and count information in `cleanBc` slot to create reads count or UMI count matrix, with barcodes in rows and samples in columns.

```
bc_2matrix(bc_obj_umi_sub)
#>                                  X781_mixa X781_mixb
#> AAGTCCAGTACTATCGTACTA                    6         0
#> AAGTCCAGTACTGTAGCTACTA                  50        37
#> AAGTCCAGTATCGTTACGCTACTA                53        43
#> AAGTCCAGTTCTACTATCGTTACGAGCTACTA         4         7
#> AAGTCCATCGTAGCTACTA                     13        20
```

More

You can use:

- `+`: to combine two BarcodeObj objects.
- `-`: to remove barcodes in a black list.
- `*`: Only include barcodes in a white list.

For examples:

```
data(bc_obj)

# Join two samples with different barcodes
bc_obj["AGAG", "test1"] + bc_obj["AAAG", "test1"]
#> Bonjour le monde, This is a BarcodeObj.
#> ----------
#> It contains:
#> ----------
#> @metadata: 6 field(s) available:
#> raw_read_count.bc_obj["AGAG", "test1"]  barcode_read_count.bc_obj["AGAG", "test1"]
↪  depth_cutoff.bc_obj["AGAG", "test1"]  raw_read_count.bc_obj["AAAG", "test1"]
↪  barcode_read_count.bc_obj["AAAG", "test1"]  depth_cutoff.bc_obj["AAAG", "test1"]
```

```
#> ----------
#> @messyBc: 1 sample(s) for raw barcodes:
#>     In sample $test1 there are: 6 Tags
#> ----------
#> @cleanBc: 1 samples for cleaned barcodes
#>     In sample $test1 there are: 2 barcodes

# Join two samples with shared barcodes
bc_obj_join <- bc_obj["AGAG", "test1"] + bc_obj["AGAG", "test1"]
#> Warning in merge.data.frame(metadata_x, metadata_y, by = 0, all = TRUE, : column names
↪  'raw_read_count.bc_obj["AGAG", "test1"]', 'barcode_read_count.bc_obj["AGAG",
↪  "test1"]', 'depth_cutoff.bc_obj["AGAG", "test1"]' are duplicated in the result
bc_obj_join
#> Bonjour le monde, This is a BarcodeObj.
#> ----------
#> It contains:
#> ----------
#> @metadata: 6 field(s) available:
#> raw_read_count.bc_obj["AGAG", "test1"]  barcode_read_count.bc_obj["AGAG", "test1"]
↪  depth_cutoff.bc_obj["AGAG", "test1"]  raw_read_count.bc_obj["AGAG", "test1"]
↪  barcode_read_count.bc_obj["AGAG", "test1"]  depth_cutoff.bc_obj["AGAG", "test1"]
#> ----------
#> @messyBc: 1 sample(s) for raw barcodes:
#>     In sample $test1 there are: 3 Tags
#> ----------
#> @cleanBc: 1 samples for cleaned barcodes
#>     In sample $test1 there are: 1 barcodes

# In this case, the shared barcodes are not merged.
# Applying bc_cure_depth() to merge them.
bc_cure_depth(bc_obj_join)
#> Bonjour le monde, This is a BarcodeObj.
#> ----------
#> It contains:
#> ----------
#> @metadata: 7 field(s) available:
#> raw_read_count.bc_obj["AGAG", "test1"]  barcode_read_count.bc_obj["AGAG", "test1"]
↪  depth_cutoff.bc_obj["AGAG", "test1"]  raw_read_count.bc_obj["AGAG", "test1"].1
↪  barcode_read_count.bc_obj["AGAG", "test1"].1  depth_cutoff.bc_obj["AGAG", "test1"].1
↪  depth_cutoff
#> ----------
#> @messyBc: 1 sample(s) for raw barcodes:
#>     In sample $test1 there are: 3 Tags
#> ----------
#> @cleanBc: 1 samples for cleaned barcodes
#>     In sample $test1 there are: 1 barcodes

# Remove barcodes
bc_obj - "AAAG"
#> Bonjour le monde, This is a BarcodeObj.
#> ----------
#> It contains:
#> ----------
```

```
#> @metadata: 3 field(s) available:
#> raw_read_count  barcode_read_count  depth_cutoff
#> ----------
#> @messyBc: 2 sample(s) for raw barcodes:
#>     In sample $test1 there are: 7 Tags
#>     In sample $test2 there are: 7 Tags
#> ----------
#> @cleanBc: 2 samples for cleaned barcodes
#>     In sample $test1 there are: 3 barcodes
#>     In sample $test2 there are: 4 barcodes

# Select barcodes in white list
bc_obj * "AAAG"
#> Bonjour le monde, This is a BarcodeObj.
#> ----------
#> It contains:
#> ----------
#> @metadata: 3 field(s) available:
#> raw_read_count  barcode_read_count  depth_cutoff
#> ----------
#> @messyBc: 2 sample(s) for raw barcodes:
#>     In sample $test1 there are: 3 Tags
#>     In sample $test2 there are: 2 Tags
#> ----------
#> @cleanBc: 2 samples for cleaned barcodes
#>     In sample $test1 there are: 1 barcodes
#>     In sample $test2 there are: 1 barcodes
```

What's more, by combining several functions, it is possible to accomplish more complex tasks. In the following example, a barcode from sample "781_mixa" is selected, then output the result in `data.frame` format.

```
bc_2df(bc_obj_umi_sub[bc_barcodes(bc_obj_umi_sub)[1], "781_mixa"])
#>   sample_name              barcode_seq count
#> 1    781_mixa AAGTCCAGTATCGTTACGCTACTA    53
## 1. Use `bc_barcodes` to pull out all the barcodes in two
##    samples, and choose the fist barcode.
## 2. Select the barcode got in step 1, and the sample named "781_mixa".
## 3. Convert the BarcodeObj object to a data.frame.
```

Part 2: Simulating bulk cellular barcode sequencing data

The `CellBarcodeSim` package includes functions to simulate cellular barcode sequencing data with 4 steps:

1. Barcode library simulation.
2. Barcode labeling, and cell growing.
3. PCR.
4. Sequencing.

Followings are simple examples to demostrate how it works, more configuration and updated documents please refer to the github page.

Install the package:

```r
install.packages(remotes)
remotes::install_github("TeamPerie/CellBarcodeSim")
```

Example 1: Simulate a Barcode sequencing experiment without UMI

```r
library(CellBarcodeSim)
```

Load barcode library

```r
barcode_library_file = system.file("data", "random_barcodes.tsv", package =
↪  "CellBarcodeSim")
```

Simulate a Barcode sequencing experiment

```r
## Simulate a Barcode sequencing experiment
simulate_main(
    ## Define the barcode library
    barcode_library_file = barcode_library_file,
    ## Define the number of clones
    clone_n              = 20,
    ## Define the clone size distribution
    clone_size_dist      = "uniform",
    ## Define the parameters of the clone size distribution
    clone_size_dist_par  = list(size_max = 1000, size_min = 1),
    ## Define the number of cell cycles
    cycle                = 30,
    ## Define the efficiency of the cell cycle
    efficiency           = 0.705,
    ## Define the error rate of the PCR, mutation per base per cycle
    error                = 1e-6,
    ## Define the number of PCR reads per cell (clone_n)
    pcr_read_per_cell    = 50,
    ## Define the output prefix
    output_prefix        = "./tmp/barcode_sim" ,
    ## Define the NGS profile (refer to ART sequencing simulator manual)
    ngs_profile          = "MSv1",
    ## Define the length of the reads
    reads_length         = 35,
    ## Define the fixed sequence at the 5' end of the reads to be added
    top_seq              = "AAAAAAAAAAGGGGG",
    ## Define the fixed sequence at the 3' end of the reads to be added
    bottom_seq           = "TTTTTTTTTT",
    ## Define the length of the fixed sequence to be added
    sequence_trunk       = 10,
    ## Use the default ART binary file
    art_bin              = NULL
)
#> PCR cycle 0
#> PCR cycle 1
#> PCR cycle 2
#> PCR cycle 3
#> PCR cycle 4
#> PCR cycle 5
```

```
#> PCR cycle 6
#> PCR cycle 7
#> PCR cycle 8
#> PCR cycle 9
#> PCR cycle 10
#> PCR cycle 11
#> PCR cycle 12
#> PCR cycle 13
#> PCR cycle 14
#> PCR cycle 15
#> PCR cycle 16
#> PCR cycle 17
#> PCR cycle 18
#> PCR cycle 19
#> PCR cycle 20
#> PCR cycle 21
#> PCR cycle 22
#> PCR cycle 23
#> PCR cycle 24
#> PCR cycle 25
#> PCR cycle 26
#> PCR cycle 27
#> PCR cycle 28
#> PCR cycle 29
#> $library_fasta
#> ./tmp/barcode_sim_library.fasta
#>
#> $sequencing_result
#> [1] "./tmp/barcode_sim"
```

Example 2: Simulating a Barcode sequencing experiment with UMI

Here we generated 20 clones with uniform size distribution from 1 to 200. The barcode library is a random barcode library 10 bp long.

After 5 cycle of pre-amplification, 8 bp UMI is added to the 5 end of the barcode. The UMI tagging efficiency is 0.05. Then 15 (20 - 5) PCR cycles are performed.

The sequencing is simulated by ART sequencing simulator, with MSv1 sequencing profile.

```
barcode_library = system.file("data", "random_barcodes.tsv", package = "CellBarcodeSim")

## Simulate a barcode sequencing experiment
simulate_main_umi(
    ## Define the barcode library
    barcode_library_file = barcode_library,
    ## Define the number of clones
    clone_n              = 20,
    ## Define the clone size distribution
    clone_size_dist      = "uniform",
    ## Define the parameters of the clone size distribution
    clone_size_dist_par  = list(size_max = 200, size_min = 1),
    ## Define the number of cell cycles
    cycle                = 20,
```

```r
    ## Define the efficiency of the cell cycle
    efficiency            = 0.705,
    ## Define the error rate of the PCR, mutation per base per cycle
    error                 = 1e-6,
    ## Define the number of PCR reads per cell (clone_n)
    pcr_read_per_umi      = 50,
    ## Define the output prefix
    output_prefix         = "./tmp/simu_seq",
    ## Define the NGS profile (refer to ART sequencing simulator manual)
    ngs_profile           = "MSv1",
    ## Define the length of the reads
    reads_length          = 35,
    ## Define the fixed sequence at the 5' end of the reads to be added
    top_seq               = "AAAAAAAAAAGGGGG",
    ## Define the fixed sequence at the 3' end of the reads to be added
    bottom_seq            = "TTTTTTTTTT",
    ## Define the length of the fixed sequence to be added
    sequence_trunk        = 10,
    ## Define the number of pre-amplification cycles
    preamp_n              = 5,
    ## Define the length of the UMI
    umi_length            = 8,
    ## Define the UMI tagging efficiency
    umi_tagging_efficiency = 0.05,
    ## Use the default ART binary file
    art_bin               = NULL
)
#> PCR cycle 0
#> PCR cycle 1
#> PCR cycle 2
#> PCR cycle 3
#> PCR cycle 4
#> PCR cycle 0
#> PCR cycle 1
#> PCR cycle 2
#> PCR cycle 3
#> PCR cycle 4
#> PCR cycle 5
#> PCR cycle 6
#> PCR cycle 7
#> PCR cycle 8
#> PCR cycle 9
#> PCR cycle 10
#> PCR cycle 11
#> PCR cycle 12
#> PCR cycle 13
#> PCR cycle 14
#> $library_fasta
#> ./tmp/simu_seq_library.fasta
#>
#> $sequencing_result
#> [1] "./tmp/simu_seq"
```

Example 3: Simulating a barcode library with constant 5 end region

For the non-UMI sequencing simulation, the constant region is added after PCR amplification in the simulating function.

If user wants to simulate a barcode library with constant region added before PCR amplification, the constant region should be constructed during the library simulating stage.

Following is an example of simulating a barcode library with constant 5 end region.

```
## Simulating a random barcode with log-normal distribution
## The mean of the lognormal distribution is 0.5 and the standard deviation is 0.1
temp_dir = tempdir()
library = simu_barcode_random_lnorm(
    length = 15,    ## the length of the barcode sequences
    n = 1e5,        ## the number of barcode sequences
    log_mean= 0.5,  ## the mean of the lognormal distribution
    log_sd= 1,    ## the standard deviation of the lognormal distribution
    top_fix = "AAAAAAATTTTTTT", ## Just for exmaple, the 5 end constant region
    bottom_fix = "AAAAAAATTTTTTT" ## Just for exmaple, the 3 end constant region
)

head(library[order(library$freq, decreasing = T), ])
#>                     seq freq                                            x
#> 34090 CCCTACCGCACCTCG   82 AAAAAAATTTTTTTCCCTACCGCACCTCGAAAAAAATTTTTTT
#> 65931 GGGCAAGATTTCTGC   75 AAAAAAATTTTTTTGGGCAAGATTTCTGCAAAAAAATTTTTTT
#> 10533 ACGGGGGTTTTCCAG   74 AAAAAAATTTTTTTACGGGGGTTTTCCAGAAAAAAATTTTTTT
#> 18426 AGTTAAACATACGGA   74 AAAAAAATTTTTTTAGTTAAACATACGGAAAAAAAATTTTTTT
#> 34563 CCGACAACTGTCCAG   73 AAAAAAATTTTTTTCCGACAACTGTCCAGAAAAAAATTTTTTT
#> 92404 TGTACTTAGTTGAAC   73 AAAAAAATTTTTTTTGTACTTAGTTGAACAAAAAAATTTTTTT
```

Using the above barcode library in the seqeucning simulation, the constant region will be affected by PCR errors.

Session Info

```
sessionInfo()
#> R version 4.3.1 (2023-06-16)
#> Platform: x86_64-apple-darwin20 (64-bit)
#> Running under: macOS Monterey 12.7.2
#>
#> Matrix products: default
#> BLAS:
↪  /Library/Frameworks/R.framework/Versions/4.3-x86_64/Resources/lib/libRblas.0.dylib
#> LAPACK:
↪  /Library/Frameworks/R.framework/Versions/4.3-x86_64/Resources/lib/libRlapack.dylib;
↪  LAPACK version 3.11.0
#>
#> locale:
#> [1] en_US.UTF-8/UTF-8/en_US.UTF-8/C/en_US.UTF-8/en_US.UTF-8
#>
#> time zone: Europe/Paris
#> tzcode source: internal
#>
#> attached base packages:
```

```
#> [1] stats     graphics  grDevices utils     datasets  methods   base
#>
#> other attached packages:
#> [1] CellBarcodeSim_0.0.0.9000 magrittr_2.0.3            plyr_1.8.9
↪  CellBarcode_1.7.1         ggplot2_3.4.4             data.table_1.14.8
↪  rmarkdown_2.25            nvimcom_0.9-159
#>
#> loaded via a namespace (and not attached):
#>  [1] tidyselect_1.2.0            dplyr_1.1.3                 farver_2.1.1
↪  Biostrings_2.68.1           bitops_1.0-7                fastmap_1.1.1
↪  RCurl_1.98-1.12             GenomicAlignments_1.36.0    egg_0.4.5
↪  digest_0.6.33
#> [11] lifecycle_1.0.3             compiler_4.3.1              rlang_1.1.1
↪  sass_0.4.7                  tools_4.3.1                 utf8_1.2.4
↪  yaml_2.3.7                  knitr_1.45                  S4Arrays_1.0.6
↪  labeling_0.4.3
#> [21] bit_4.0.5                   interp_1.1-4                DelayedArray_0.26.7
↪  RColorBrewer_1.1-3          abind_1.4-5                 ShortRead_1.58.0
↪  BiocParallel_1.34.2         withr_2.5.2                 hwriter_1.3.2.1
↪  BiocGenerics_0.46.0
#> [31] grid_4.3.1                  stats4_4.3.1                fansi_1.0.5
↪  latticeExtra_0.6-30         Ckmeans.1d.dp_4.3.5         colorspace_2.1-0
↪  scales_1.2.1                tinytex_0.48                SummarizedExperiment_1.30.2
↪  cli_3.6.1
#> [41] crayon_1.5.2                generics_0.1.3              tzdb_0.4.0
↪  cachem_1.0.8                stringr_1.5.0               zlibbioc_1.46.0
↪  parallel_4.3.1              formatR_1.14                XVector_0.40.0
↪  matrixStats_1.0.0
#> [51] vctrs_0.6.4                 Matrix_1.6-1.1              jsonlite_1.8.7
↪  IRanges_2.34.1              hms_1.1.3                   S4Vectors_0.38.2
↪  bit64_4.0.5                 jpeg_0.1-10                 jquerylib_0.1.4
↪  glue_1.6.2
#> [61] codetools_0.2-19            stringi_1.7.12              gtable_0.3.4
↪  GenomeInfoDb_1.36.4         deldir_1.0-9                GenomicRanges_1.52.1
↪  munsell_0.5.0               tibble_3.2.1                pillar_1.9.0
↪  htmltools_0.5.6.1
#> [71] GenomeInfoDbData_1.2.10     R6_2.5.1                    Rdpack_2.5
↪  vroom_1.6.4                 evaluate_0.23               lattice_0.22-5
↪  Biobase_2.60.0              readr_2.1.4                 highr_0.10
↪  rbibutils_2.2.16
#> [81] png_0.1-8                   Rsamtools_2.16.0            bslib_0.5.1
↪  Rcpp_1.0.11                 gridExtra_2.3               xfun_0.41
↪  MatrixGenerics_1.12.3       pkgconfig_2.0.3
```

Supplementary vignette 2

```r
library(data.table)
library(ggplot2)
library(CellBarcode)
```

Introduction

Lineage barcode labeling is usually done by adding the lineage barcode sequence to DNA. The sequence can be transcripted and thus detected by scRNASeq. There is another vignette, which is about bulk sample barcode analysis, will be very helpful, highly recommend reading that in addition to this one.

This vignette is a tutorial for how to extract lineage barcodes from scRNASeq data and do the cleaning of them. It also can be applied to analyze other single-cell sequencing results such as scATACSeq, scDNASeq, as long as the barcodes are sequenced. For the analysis, the input is `fastq` files or the `bam` file.

Fastq Files

> The `fastq` files are raw reads. To use it, you need the design of the lineage barcode, cell barcode, and (or) UMI sequence. Given the structure, the CellBarcode will search them. To facilitate detection, the target amplification can be carried out. The target amplification is a PCR process, which uses the barcode-specific primer to amplify the barcode sequence using scRNASeq library. The targeted amplified and normal scRNASeq Data both can be used as input of CellBarcode.

Bam Files

> The `bam` file contains the reads with alignment information, in which the cell barcode and UMI sequences have been digested. The CellBarcode search barcode in `bam` file using a regular expression, and find the cell barcodes and(or) UMI sequences by the `bam` tag. In most cases, in

the `bam` file, the barcode sequence is not aligned to the reference genome as they are not part of the genome, thus we can do a quick filtering to get the un-mapped reads, as we will demonstrate later, to reduce the running time of barcode searching.

Extract barcodes from fastq file

Extract lineage barcode

We use the targeted amplified fastq file derived from Cosgrove et al. 2023 as an example.

```
s1_file <- c(

    ↪  "../run_scRNASeq_example_cosgrove/data/KDI_2015730_2023-01-31_14-58-15/L438T02/L438T02.R1.fastq
    ↪

    ↪  "../run_scRNASeq_example_cosgrove/data/KDI_2015730_2023-01-31_14-58-15/L438T02/L438T02.R2.fastq

bc_qc <- bc_seq_qc(s1_file)

bc_plot_seqQc(bc_qc)
```

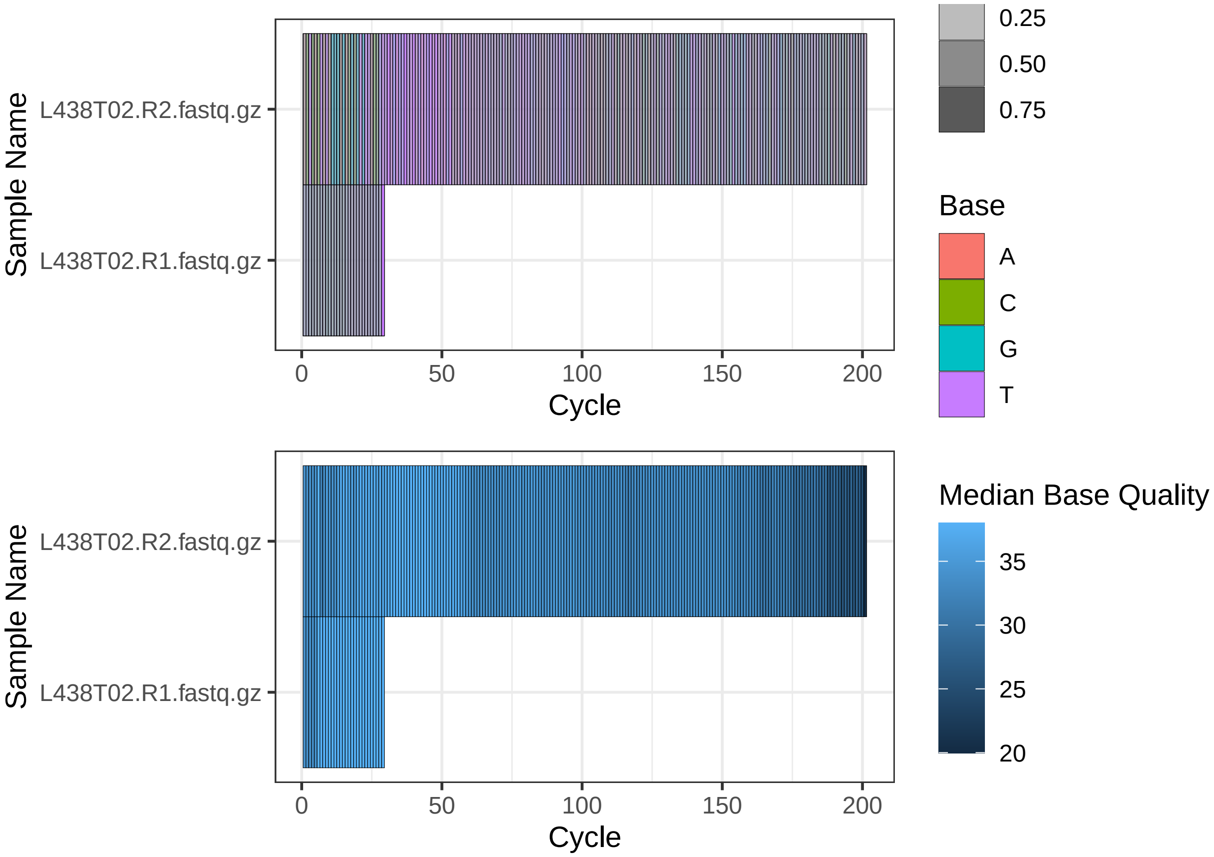

It is a 10X Genomics scRNASeq data with a paired-end sequencing.

Read 1 has 28bp, containing 16bp cellular barcode followed by 12bp UMI. In the code, regular expression `(.{16)` defines the 16bp cellular barcode pattern; `.{16}(.{12})` is for the UMI. In the regular expression, `.` means any character, and the number, followed by 16 surrounded by `{}` which tells the repeat number; `()` defines the sequences interested and returned as results, outside sequence serves as context for defining the

target.

Read 2 contains the lineage barcode, which can be identified by the pattern `CGAAGTATCAAG(.+)CCGTAGCAAG`. The `CGAAGTATCAAG` and `CCGTAGCAAG` are constant sequences around barcodes that are defined by `(.+)`. The `+` is quantification similar to `{16}`, indicating the barcode sequence is flexible length at least 1 bp.

The output `bc_obj1` is a `BarcodeObj` object, using which we have provided functions to check quality, filter, and export results. The `BarcodeObj` has the same data structure as the one generated in bulk analysis. The difference is that in the scRNASeq barcode analysis, the sample name is the cell barcode. Consequently, the `BarcodeObj` contains only one scRNASeq sample.

```
bc_obj1 = bc_extract_sc_fastq(
    fq1 = s1_file[1],                                   ## fastq file 1
    fq2 = s1_file[2],                                   ## fastq file 2
    patternCellBarcode = "(.{16})",                     ## Cell barcode pattern
    patternUMI = ".{16}(.{12})",                        ## UMI pattern
    patternBarcode = "CGAAGTATCAAG(.+)CCGTAGCAAG"    ## Lineage barcode pattern
)
```

More about the pattern

The pattern is a regular expression, followed by which CellBarcode finds the lineage barcode, cell barcode, and (or) UMI. The following are brief descriptions of syntax, just to help you work with simple cases without leaving this document.

In the pattern, we define the barcode backbone and label the barcode sequence by bracket `()`.

For example, the pattern `ATCG(.{21})TCGG` tells the barcode is surrounded by a constant sequence of `ATCG`, and `TCGG`. Following are some examples to define the constant region and barcode sequence.

Example 1

`ATCG(.{21})`

21 bases barcode after a constant sequence of "ATCG".

Example 2

`(.{15})TCGA`

15 bases barcode before a constant sequence of "TCGA".

Example 3

`ATCG(.*)TCGA`

A barcode with a flexible length located between the constant regions "ATCG" and "TCGA".

Need more help: For a more complex barcode pattern, please ask the package author.

Check the quality of extracted barcodes

The `bc_plot_count` tells you some numbers of extracted barcodes in the `bc_obj1`.

1. reads count (with barcode) versus the total reads
2. reads count per UMI
3. UMI count per barcode
4. barcode count per sample

```r
bc_plot_count(bc_obj1, useClean=F)
#> Warning: Transformation introduced infinite values in continuous y-axis
#> Warning: Removed 3 rows containing missing values (`geom_bar()`).
```

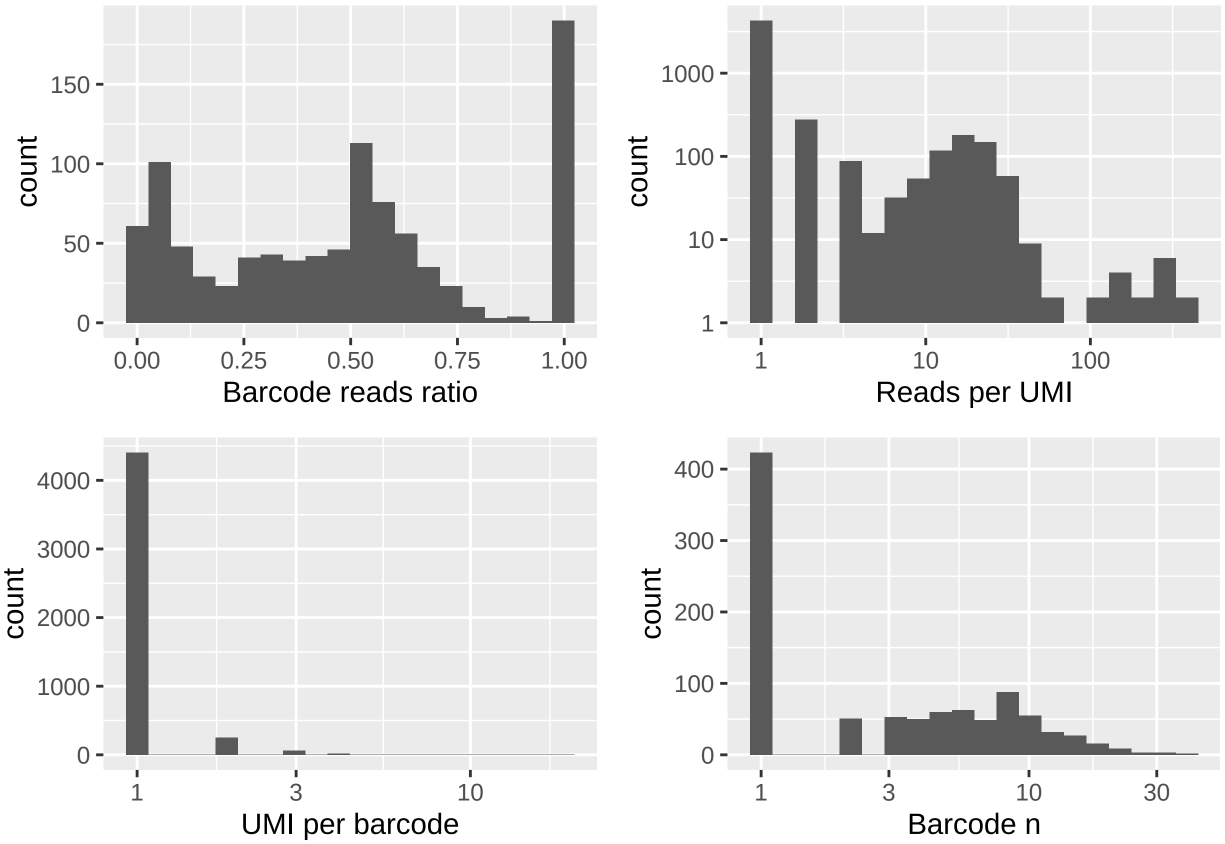

You also can use `bc_meta` to check the metadata useful for quality control in the `bc_obj1`.

```r
head(bc_meta(bc_obj1))
#>                  raw_read_count barcode_read_count
#> AAACGCCTCGACGATT              1                  1
#> AAAGAACAGTATTCCG             35                  1
#> AAAGGGCAGTTGGCTT             67                 18
#> AAAGGGCTCCATTCGC             17                  1
#> AAAGGTAAGGAATGTT             10                  1
#> AAAGTCCAGAACCGCA            370                258
```

Filter barcode

We filter the barcode by UMI reads count, which is a reads number with the same UMI-barcode sequence. A higher UMI reads count shows more confidence in a barcode sequence.

For previous barcode number distribution, we can see a UMI can link to multiple barcodes. Ideally, each UMI should link to only one barcode sequence when UMI diversity is far higher than the barcode. In the unique UMI case, the `isUniqueUMI` parameter should be set to keep the most frequent barcode for each UMI.

```r
## Count the UMI with at least 3 reads
bc_obj = bc_cure_umi(bc_obj1, depth = 3, isUniqueUMI = T)
```

Next, we filter the barcode by the UMI count which is the UMI number a barcode sequence has.

```
## Keep the barcodes with at least 2 UMIs
bc_obj = bc_cure_depth(bc_obj, depth = 2)
```

We can export the barcode sequence to other R data types such as `data.frame`, `data.table` or `data.matrix`.

```
## export data.frame
d <- bc_2df(bc_obj)
head(d)
#>        sample_name                  barcode_seq count
#> 1 AAAGTCCAGAACCGCA TCCAGATCTACTATCGTTACGGCTACTA     3
#> 2 AACAAAGTCTTCTGGC     TCCAGTATCGTTACGACGCTACTA     3
#> 3 AACCATGAGCTCTTCC    TCCAGTCGTTACGACGTAGCTACTA     3
#> 4 AAGTACCCATAATGCC     TCCAGTATCGTTACGACGCTACTA     3
#> 5 AATTCCTTCTATTGTC     TCCAGTATCGTTACGACGCTACTA     2
#> 6 AATTTCCTCACGATAC     TCCCTTATCGTTACGACGCTACTA     2

## export data.table
d <- bc_2df(bc_obj)
head(d)
#>        sample_name                  barcode_seq count
#> 1 AAAGTCCAGAACCGCA TCCAGATCTACTATCGTTACGGCTACTA     3
#> 2 AACAAAGTCTTCTGGC     TCCAGTATCGTTACGACGCTACTA     3
#> 3 AACCATGAGCTCTTCC    TCCAGTCGTTACGACGTAGCTACTA     3
#> 4 AAGTACCCATAATGCC     TCCAGTATCGTTACGACGCTACTA     3
#> 5 AATTCCTTCTATTGTC     TCCAGTATCGTTACGACGCTACTA     2
#> 6 AATTTCCTCACGATAC     TCCCTTATCGTTACGACGCTACTA     2

## export data.matrix
d <- bc_2matrix(bc_obj)
d[1:5, 1:3]
#>                               AAAGTCCAGAACCGCA AACAAAGTCTTCTGGC AACCATGAGCTCTTCC
#> TCCAATTACTATCGTTACGGTAGCTACTA                0                0                0
#> TCCACTACTATCGTTACGTACTA                      0                0                0
#> TCCACTATCGTTACGACGCTACTA                     0                0                0
#> TCCACTATCGTTACGACGTAGCTACTA                  0                0                0
#> TCCACTATCGTTACGACTACTA                       0                0                0
```

Explore the threshold

We explore how the UMI read count threshold and UMI count threshold affect the result by trying a range of values.

UMI read count threshold

Assuming the UMI should be unique, the result shows that the UMI read count threshold of 2 is enough good, which keeps most of the UMI with a unique barcode. The far more stringent threshold leads to little improvement and may cause the loss of some barcodes.

```
## Try the UMI read count threshold from 1 to 7
d_plot = lapply(1:7, function(i) {
    bc_obj = bc_cure_umi(bc_obj1, depth = i, isUniqueUMI = T)
    d = bc_2dt(bc_obj)
    d = d[, .(barcode_seq = unique(barcode_seq)), by = sample_name][, .N, by =
↪   sample_name]
```

```
    d$filter = i
    d
})
d_plot <- rbindlist(d_plot)

d_plot_plus = d_plot[, .N, by = filter]
## Scale the left and right y-axis
a = 200
b = 150

## Plot the result
ggplot(d_plot) + aes(x = factor(filter), y = N) +
    geom_violin() +
    geom_point(position = position_jitter(w = 0.3, h = 0), alpha = 0.2) +
    geom_line(data = d_plot_plus, aes(x = filter, y = (N - a) / b), color = "red") +
    geom_point(data = d_plot_plus, aes(x = filter, y = (N - a) / b), color = "red") +
    scale_y_continuous(
        name = 'Barcodes per cell',
        sec.axis = sec_axis(~ (. * b + a),name = "Cell number")) +
    labs(x = "UMI read count") + theme_bw()
```

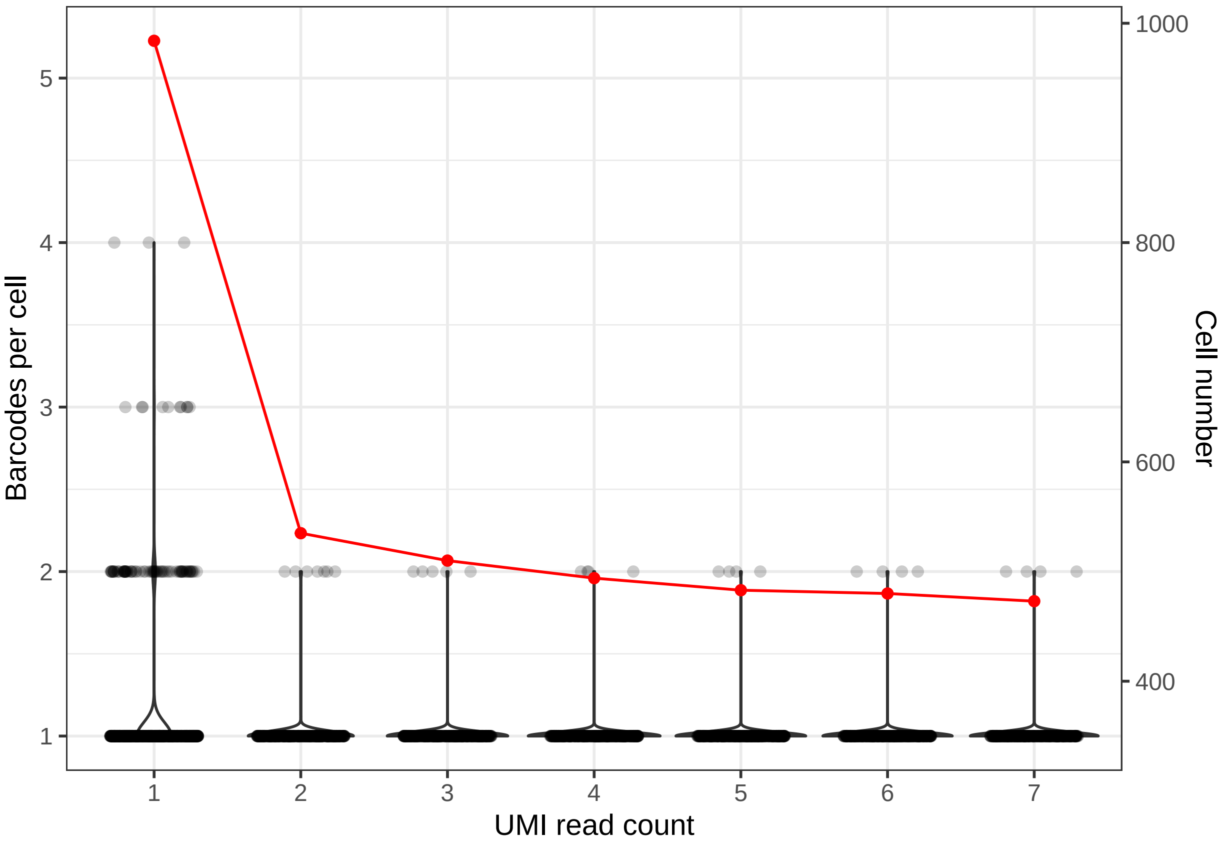

UMI count threshold

By fixing the UMI read count threshold to 2, we explore the effect of the UMI count threshold.

For the dataset, we demonstrated here, each cell only has one copy of the unedited VDJ sequence, which guarantees a unique barcode per cell. Using this criterion we test the UMI count threshold.

We can see that a UMI count threshold of 2 ensures that the majority of cells possess a unique barcode. Increasing the threshold further provides minimal benefits and may result in the loss of certain barcodes.

```
## Fix the UMI read count threshold to 2
bc_obj = bc_cure_umi(bc_obj1, depth = 2, isUniqueUMI = T)

## Try the UMI count threshold from 1 to 5
d_plot = lapply(1:5, function(i) {
    bc_obj = bc_cure_depth(bc_obj, depth = i)
    d = bc_2dt(bc_obj)
    d = d[, .(barcode_seq = unique(barcode_seq)), by = sample_name][, .N, by =
↪   sample_name]
    d$filter = i
    d
})

d_plot <- rbindlist(d_plot)

d_plot_plus = d_plot[N == 1, .N, by = filter]
d_plot_plus = d_plot[, .N, by = filter]
## Scale the left and right y-axis
a = -200
b = 200

## Plot the result
ggplot(d_plot) + aes(x = factor(filter), y = N) +
    geom_violin() +
    geom_point(position = position_jitter(w = 0.3, h = 0), alpha = 0.2) +
    geom_line(data = d_plot_plus, aes(x = filter, y = ((N - a) / b)), color = "red") +
    geom_point(data = d_plot_plus, aes(x = filter, y = ((N - a) / b)), color = "red") +
    scale_y_continuous(
        name = 'Barcodes per cell',
        sec.axis = sec_axis(~ (. * b + a),
        name = "Cell has \n Unique barcode")) +
    labs(x = "UMI count") + theme_bw()
```

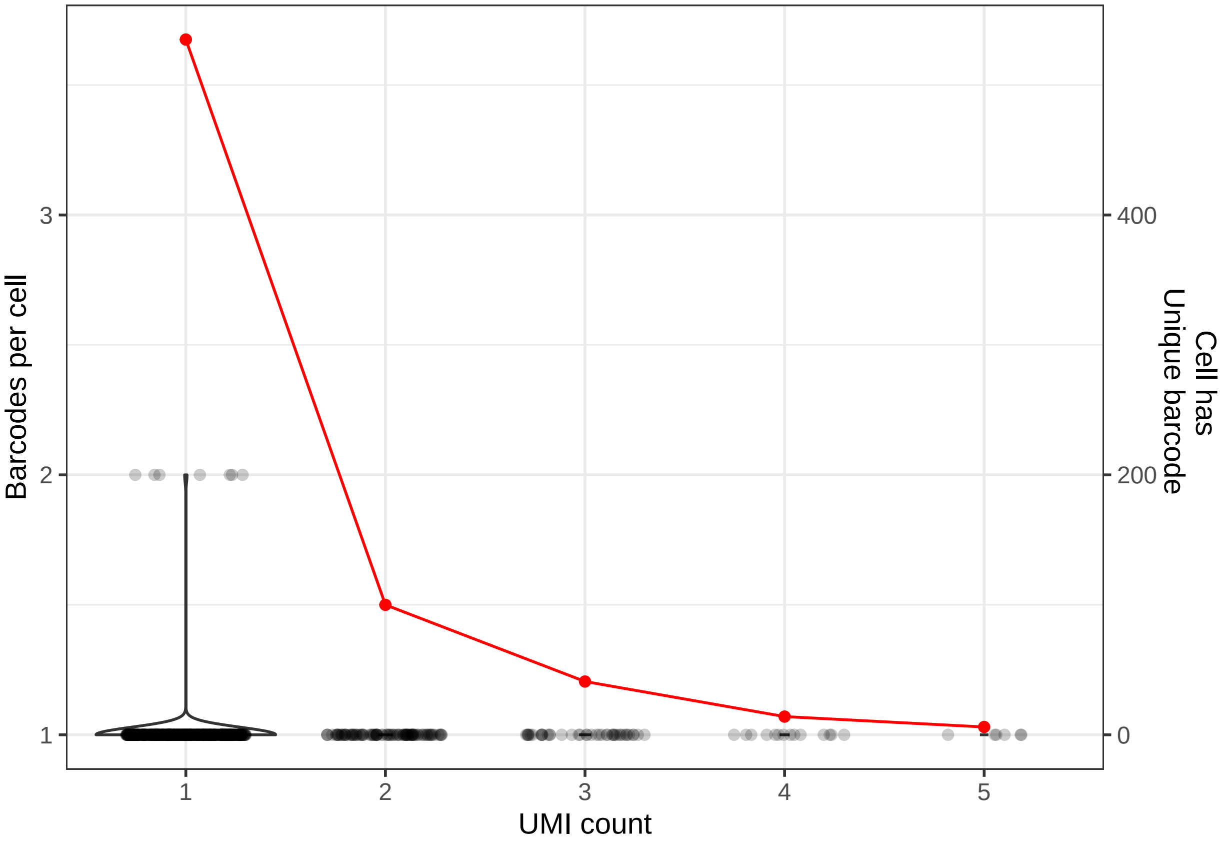

Extract lineage barcodes from a sam/bam file

Preprocess sam/bam file

A sam/bam file has RNA sequences, cell barcode, and UMI. We need to get the barcode in the RNA sequence together with the Cell barcode and UMI by tags.

For 10X scRNASeq usually, you can find the bam file in in following location of the CellRanger output:

`CellRanger Output fold/outs/possorted_genome_bam.bam`

If you use a customized pipeline, you need to know the answer or who to ask for assistance.

Why preprocess. We do filtering with simple criteria, which make the input file smaller, to reduce the running time.

Example: get the sam/bam file only containing un-mapped reads

Using unmapped reads can significantly reduce the running time of the barcode extraction procedure, especially when barcodes do not map to the reference genome sequence. By running the following code, we obtain a `scRNASeq_10X.sam` file that contains only the unmapped reads.

```
samtools view -f 4 possorted_genome_bam.bam > scRNASeq_10X.sam
```

Extract lineage barcode

The following example extracts the lineage barcode together with the cellular barcode and UMI information.

The parameters:

- `sam`: The location of the sam/bam file.

- `pattern`: A regular expression describing lineage barcode.
- `cell_barcode_tag`: The cell barcode tag in the sam/bam file, the default is "CR" in the Cell Ranger output.
- `umi_tag`: The UMI field tag in the sam/bam file, the default is "UR" in the Cell Ranger output.

```
sam_file <- system.file("extdata", "scRNASeq_10X.sam", package = "CellBarcode")

d = CellBarcode::bc_extract_sc_sam(
   sam = sam_file,
   pattern = "AGATCAG(.*)TGTGGTA",
   cell_barcode_tag = "CR",
   umi_tag = "UR"
)
```

The output is a `BarcodeObj`, which is the same as the object generated in the previous case.

Cleaning lineage barcode

This section will give you a very short example of cleaning the lineage barcodes because we did that in the previous example.

Please attention that the dataset used in this section is a subset of a dataset with the read count lower than a real one. So the filtering applied here is for demonstration purposes.

```
## Filter UMI reads count, keep the UMI with at least 2 reads
d_sub <- bc_cure_umi(d, depth = 1)

## Filter the UMI count, here no UMI count filter is applied
d_sub <- bc_cure_depth(d_sub, depth = 1)

head(bc_2df(d_sub))
#>         sample_name           barcode_seq count
#> 1 AAACGAAAGTTCATGC ACACATTGTTATACGTAATAA     1
#> 2 AAACGCTAGCTGACCC TACCTAAGGTCGAATTTGCTA     1
#> 3 AAAGGATAGTGTGTTC CGTTAATCGGTCTAGTGATTA     1
#> 4 AAAGGGCAGCCTGAGA CGTCGATTGTGCAAACGTAGA     1
#> 5 AAAGGTAAGGCCCACT          ATGGTTGTAGATG     1
#> 6 AAAGTCCCACTGCGAC AGACAACTTGGATTTTGTGTA     1
```

Session Info

```
sessionInfo()
#> R version 4.3.1 (2023-06-16)
#> Platform: x86_64-apple-darwin20 (64-bit)
#> Running under: macOS Monterey 12.7.2
#>
#> Matrix products: default
#> BLAS:
↪  /Library/Frameworks/R.framework/Versions/4.3-x86_64/Resources/lib/libRblas.0.dylib
#> LAPACK:
↪  /Library/Frameworks/R.framework/Versions/4.3-x86_64/Resources/lib/libRlapack.dylib;
↪  LAPACK version 3.11.0
#>
```

```
#> locale:
#> [1] en_US.UTF-8/UTF-8/en_US.UTF-8/C/en_US.UTF-8/en_US.UTF-8
#>
#> time zone: Europe/Paris
#> tzcode source: internal
#>
#> attached base packages:
#> [1] stats     graphics  grDevices utils     datasets  methods   base
#>
#> other attached packages:
#> [1] CellBarcode_1.7.1 ggplot2_3.4.4     data.table_1.14.8 rmarkdown_2.25
↪  nvimcom_0.9-159
#>
#> loaded via a namespace (and not attached):
#>  [1] SummarizedExperiment_1.30.2 gtable_0.3.4                xfun_0.41
↪  bslib_0.5.1                 hwriter_1.3.2.1             latticeExtra_0.6-30
↪  Biobase_2.60.0              lattice_0.22-5              Rdpack_2.5
↪  vctrs_0.6.4
#> [11] tools_4.3.1                 bitops_1.0-7                generics_0.1.3
↪  stats4_4.3.1                parallel_4.3.1              tibble_3.2.1
↪  fansi_1.0.5                 highr_0.10                  pkgconfig_2.0.3
↪  Matrix_1.6-1.1
#> [21] RColorBrewer_1.1-3          S4Vectors_0.38.2            lifecycle_1.0.3
↪  GenomeInfoDbData_1.2.10     farver_2.1.1                stringr_1.5.0
↪  deldir_1.0-9                compiler_4.3.1              egg_0.4.5
↪  Rsamtools_2.16.0
#> [31] Biostrings_2.68.1           Ckmeans.1d.dp_4.3.5         munsell_0.5.0
↪  codetools_0.2-19            GenomeInfoDb_1.36.4         htmltools_0.5.6.1
↪  sass_0.4.7                  RCurl_1.98-1.12             yaml_2.3.7
↪  pillar_1.9.0
#> [41] crayon_1.5.2                jquerylib_0.1.4             BiocParallel_1.34.2
↪  cachem_1.0.8                DelayedArray_0.26.7         ShortRead_1.58.0
↪  abind_1.4-5                 tidyselect_1.2.0            digest_0.6.33
↪  stringi_1.7.12
#> [51] dplyr_1.1.3                 labeling_0.4.3              fastmap_1.1.1
↪  grid_4.3.1                  colorspace_2.1-0            cli_3.6.1
↪  magrittr_2.0.3              S4Arrays_1.0.6              utf8_1.2.4
↪  withr_2.5.2
#> [61] scales_1.2.1                XVector_0.40.0              jpeg_0.1-10
↪  matrixStats_1.0.0           interp_1.1-4                gridExtra_2.3
↪  png_0.1-8                   evaluate_0.23               knitr_1.45
↪  rbibutils_2.2.16
#> [71] GenomicRanges_1.52.1        IRanges_2.34.1              rlang_1.1.1
↪  Rcpp_1.0.11                 glue_1.6.2                  BiocGenerics_0.46.0
↪  jsonlite_1.8.7              plyr_1.8.9                  R6_2.5.1
↪  MatrixGenerics_1.12.3
#> [81] GenomicAlignments_1.36.0    zlibbioc_1.46.0
```

Supplementary Algorithm 1

```
For (the most abundant barcode -> b_m) do:
     Count reads of most abundant barcode r_m
     For (each other less abundant barcode -> b_l)
        Count reads of less abundant barcode r_l
        delta_reads = r_m / r_l
        distance = edit distance between b_m and b_l
        if (
          (distance < threshold) and
          (depth < threshold) and
          (delta_reads > threshold)
           ):
              Remove (the less abundant barcode b_l)
        else:
              Keep (the less abundant barcode b_l)
     output (the most abundant barcode b_m)
Time complexity: O(n^2)
```
