# Supplementary material for: Extracting, filtering and simulating cellular barcodes using CellBarcode tools
Source: Nat Comput Sci. 2024 Feb 19;4(2):128–43. doi: 10.1038/s43588-024-00595-7 (PMC10899113; doi:10.1038/s43588-024-00595-7)
Supplement: Supplementary file 2 — Reporting Summary [file 43588_2024_595_MOESM2_ESM.pdf]

nature portfolio

Corresponding author(s): Wenjie Sun
Leila Perié
Anne-Marie Lyne

Last updated by author(s): Jan 12, 2024

Reporting Summary

Nature Portfolio wishes to improve the reproducibility of the work that we publish. This form provides structure for consistency and transparency in reporting. For further information on Nature Portfolio policies, see our Editorial Policies and the Editorial Policy Checklist.

Statistics

For all statistical analyses, confirm that the following items are present in the figure legend, table legend, main text, or Methods section.

n/a	Confirmed	
☐	☒	The exact sample size (*n*) for each experimental group/condition, given as a discrete number and unit of measurement
☐	☒	A statement on whether measurements were taken from distinct samples or whether the same sample was measured repeatedly
☐	☒	The statistical test(s) used AND whether they are one- or two-sided<br>*Only common tests should be described solely by name; describe more complex techniques in the Methods section.*
☒	☐	A description of all covariates tested
☒	☐	A description of any assumptions or corrections, such as tests of normality and adjustment for multiple comparisons
☐	☒	A full description of the statistical parameters including central tendency (e.g. means) or other basic estimates (e.g. regression coefficient) AND variation (e.g. standard deviation) or associated estimates of uncertainty (e.g. confidence intervals)
☐	☒	For null hypothesis testing, the test statistic (e.g. *F*, *t*, *r*) with confidence intervals, effect sizes, degrees of freedom and *P* value noted<br>*Give P values as exact values whenever suitable.*
☒	☐	For Bayesian analysis, information on the choice of priors and Markov chain Monte Carlo settings
☒	☐	For hierarchical and complex designs, identification of the appropriate level for tests and full reporting of outcomes
☐	☒	Estimates of effect sizes (e.g. Cohen's *d*, Pearson's *r*), indicating how they were calculated

Our web collection on statistics for biologists contains articles on many of the points above.

Software and code

Policy information about availability of computer code

Data collection

IGoR version 1.4.0 (https://github.com/qmarcou/IGoR)
ART ART-MountRainier-2016-06-05 (https://www.niehs.nih.gov/research/resources/software/biostatistics/art)
bartender-1.1 (https://github.com/LaoZZZZZ/bartender-1.1)
R packages:
DNABarcodes: version 1.30.0
stringi: version 1.7.12
ROCR: version 1.0-11
CellTagR: Version: 0.0.0.9000
genBaRcode: version 1.2.6

Data analysis

The barcode simulation toolkit is available at https://github.com/TeamPerie/CellBarcodeSim
CellBarcode is available at https://bioconductor.org/packages/devel/bioc/html/CellBarcode.html
Analysis scripts are available at https://zenodo.org/records/10492821

For manuscripts utilizing custom algorithms or software that are central to the research but not yet described in published literature, software must be made available to editors and reviewers. We strongly encourage code deposition in a community repository (e.g. GitHub). See the Nature Portfolio guidelines for submitting code & software for further information.

Data

Policy information about availability of data

All manuscripts must include a data availability statement. This statement should provide the following information, where applicable:

- Accession codes, unique identifiers, or web links for publicly available datasets
- A description of any restrictions on data availability
- For clinical datasets or third party data, please ensure that the statement adheres to our policy

The lentiviral barcodes from Eisele et al (2022) are available from: https://doi.org/10.5281/zenodo.5645045; the corresponding pre-analysed data is available at: https://github.com/TeamPerie/Eisele-et-al. The CellTag barcode sequencing data from Biddy et al (2018) is on GEO with dataset ID GSE99915. The Marsolier et al (2022) barcoded scRNASeq dataset is on GEO with dataset ID GSE164716. The mammary gland VDJ barcode dataset is available at https://doi.org/10.5281/zenodo.8124949. The MEF cell line mixes VDJ barcode dataset is available at: https://doi.org/10.5281/zenodo.10027002. The VDJ-barcoded scRNA-seq data from Cosgrove et al (2023) belongs to the authors of that paper and was given to us for the purposes of this paper; to obtain this data please contact Leïla Perié (leila.perie@curie.fr). Source data for Figures 2-6 are provided with this paper.

Human research participants

Policy information about studies involving human research participants and Sex and Gender in Research.

Reporting on sex and gender	No human participants
Population characteristics	No human participants
Recruitment	No human participants
Ethics oversight	No human participants

Note that full information on the approval of the study protocol must also be provided in the manuscript.

Field-specific reporting

Please select the one below that is the best fit for your research. If you are not sure, read the appropriate sections before making your selection.

☒ Life sciences ☐ Behavioural & social sciences ☐ Ecological, evolutionary & environmental sciences

For a reference copy of the document with all sections, see nature.com/documents/nr-reporting-summary-flat.pdf

Life sciences study design

All studies must disclose on these points even when the disclosure is negative.

Sample size	No statistical method was used to predetermine sample size. Simulations were repeated 30 times for each scenario, this was based on a trade-off between what was feasible computationally and having enough repeats to characterise the mean and variability.
Data exclusions	No data were excluded from the analyses.
Replication	The sequencing library construction was repeated twice and both attempts were successful.<br>CRISPR gRNA data: three technical replicates of the experiment are available, but we present analysis of one replicate as we use it to validate simulation findings.<br>VDJ-barcoded mammary gland data: only one mouse was used as the purpose was experimental protocol development. We use the data to validate our simulation findings.
Randomization	Not applicable
Blinding	Not applicable

Reporting for specific materials, systems and methods

We require information from authors about some types of materials, experimental systems and methods used in many studies. Here, indicate whether each material, system or method listed is relevant to your study. If you are not sure if a list item applies to your research, read the appropriate section before selecting a response.

Materials & experimental systems

n/a	Involved in the study
☐	☒ Antibodies
☒	☐ Eukaryotic cell lines
☒	☐ Palaeontology and archaeology
☐	☒ Animals and other organisms
☒	☐ Clinical data
☒	☐ Dual use research of concern

Methods

n/a	Involved in the study
☒	☐ ChIP-seq
☐	☒ Flow cytometry
☒	☐ MRI-based neuroimaging

Antibodies

Antibodies used

Biolegend antibodies:
APC anti-mouse CD31 (102510) Vecchi et al 1994 Eur. J. Cell Biol.
APC anti-mouse Ter119 (116212) Maraskovsky E, et al. 1996. J. Exp. Med.
APC anti-mouse CD45 (103112) Seaman WE. 1983. J. Immunol. 130:1713.
APC/Cy7 anti-mouse CD49f (313628) Sonnenberg A, et al. 1987 J. Biol. Chem.
PE anti-mouse EpCAM (118206) Farr A, et al. 1991. J. Histochem. Cytochem.

Validation

All antibodies have been used in the Fre lab for over 10 years, published in our previous studies and specificity has been validated in numerous ways including lack of expression in specific tissues.

Animals and other research organisms

Policy information about studies involving animals; ARRIVE guidelines recommended for reporting animal research, and Sex and Gender in Research

Laboratory animals

All mouse lines in this study have been described:
1. Notch1CreERT2: Fre, S. et al. Notch Lineages and Activity in Intestinal Stem Cells Determined by a New Set of Knock-In Mice. PLOS ONE 6, e25785 (2011).
2. DRAG mice: Urbanus, J. et al. DRAG in situ barcoding reveals an increased number of HSPCs contributing to myelopoiesis with age. Nat. Commun. 14, 2184 (2023).
3. Apc1638N: Fodde, R. et al. A targeted chain-termination mutation in the mouse Apc gene results in multiple intestinal tumors. Proc. Natl. Acad. Sci. 91, 8969–8973 (1994). Organoids deriving from this strain used in this study.
4. DRAG+/– Notch1CreERT2+/– cross: described in this study. Mammary gland tissue collected at 6 weeks of age.

Wild animals

No wild animals were used.

Reporting on sex

All mice used are female.

Field-collected samples

No field-collected samples were used.

Ethics oversight

All studies and procedures involving animals were in accordance with the recommendations of the European Community (2010/63/UE) for the Protection of Vertebrate Animals used for Experimental and other Scientific Purposes. Approval was provided by the ethics committee of the French Ministry of Research (reference APAFIS #34364-202112151422480). We comply with internationally established principles of replacement, reduction, and refinement in accordance with the Guide for the Care and Use of Laboratory Animals (NRC 2011). Husbandry, supply of animals, as well as maintenance and care in the Animal Facility of Institut Curie (facility license #C75–05–18) before and during experiments fully satisfied the animal’s needs and welfare.

Note that full information on the approval of the study protocol must also be provided in the manuscript.

Flow Cytometry

Plots

Confirm that:

☒ The axis labels state the marker and fluorochrome used (e.g. CD4-FITC).

☒ The axis scales are clearly visible. Include numbers along axes only for bottom left plot of group (a 'group' is an analysis of identical markers).

☒ All plots are contour plots with outliers or pseudocolor plots.

☒ A numerical value for number of cells or percentage (with statistics) is provided.

Methodology

Sample preparation

The VDJ barcode mouse was crossed with Notch1CreERT2 mouse54. Lactating mothers were injected with tamoxifen (0.1mg per g of mouse body mice, MP Biomedicals, 156738) as described 55 in order to induce Cre recombination in the progeny at

stage P0. Mammary tissue of a DRAG+/– Notch1CreERT2+/– female was then collected at 6 weeks of age and mammary single cell dissociation was performed as previously described56. Briefly, mammary fat pads were mechanically minced with scissors and scalpel and digested for 90 min at 37C in CO2-independent medium (Invitrogen, 18045-054) supplemented with 5% fetal bovine serum, 3 mg/ml collagenase A (Roche, 10103586001) and 100 U/ml hyaluronidase (Sigma, H3884). The resulting suspension was sequentially resuspended in 0.25% trypsin–EDTA for 1 min, and then 5 min in 5 mg/ml dispase (Roche, 04942078001) with 0.1 mg/ml DNase I (Sigma, D4527) followed by filtration through a 40-µm mesh. Red blood cells were lysed in NH4Cl. The obtained single cell suspension was then stained with the following Biolegend antibodies, at a 1/100 dilution: APC anti-mouse CD31 (102510), APC anti-mouse Ter119 (116212), APC anti-mouse CD45 (103112), APC/Cy7 anti-mouse CD49f (313628), and PE anti-mouse EpCAM (118206). Dead cells (DAPI+), and CD45+/CD31+/Ter119+ (Lin+) non-epithelial cells were excluded before analysis

Instrument	BD FACSAriaIIu version 09
Software	FACS Collection Software: BD FACSDiva 8.0.1. Analysis Software: FlowJo 10.8.1
Cell population abundance	In the luminal population gate, we selected GFP-positive cells for analysis, representing about 6% of the total luminal cell population. The purity of sorted populations was about 95%
Gating strategy	See Supplementary Fig. 24-25

☒ Tick this box to confirm that a figure exemplifying the gating strategy is provided in the Supplementary Information.
